# Feline Infectious Peritonitis: European Advisory Board on Cat Diseases Guidelines

**DOI:** 10.3390/v15091847

**Published:** 2023-08-31

**Authors:** Séverine Tasker, Diane D. Addie, Herman Egberink, Regina Hofmann-Lehmann, Margaret J. Hosie, Uwe Truyen, Sándor Belák, Corine Boucraut-Baralon, Tadeusz Frymus, Albert Lloret, Fulvio Marsilio, Maria Grazia Pennisi, Etienne Thiry, Karin Möstl, Katrin Hartmann

**Affiliations:** 1 Bristol Veterinary School, University of Bristol, Bristol BS40 5DU, UK; 2 Linnaeus Veterinary Limited, Shirley, Solihull B90 4BN, UK; 3 Independent Researcher, 64000 Pyrénées Aquitaine, France; draddie@catvirus.com; 4 Department of Biomolecular Health Sciences, Faculty of Veterinary Medicine, University of Utrecht, 3584 CL Utrecht, The Netherlands; h.f.egberink@uu.nl; 5 Clinical Laboratory, Department of Clinical Diagnostics and Services, Vetsuisse Faculty, University of Zurich, 8057 Zurich, Switzerland; rhofmann@vetclinics.uzh.ch; 6 MRC-University of Glasgow Centre for Virus Research, Garscube Estate, Glasgow G61 1QH, UK; margaret.hosie@glasgow.ac.uk; 7 Institute of Animal Hygiene and Veterinary Public Health, University of Leipzig, 04103 Leipzig, Germany; truyen@vetmed.uni-leipzig.de; 8 Department of Biomedical Sciences and Veterinary Public Health (BVF), Swedish University of Agricultural Sciences (SLU), P.O. Box 7036, 750 07 Uppsala, Sweden; sandor.belak@slu.se; 9 Scanelis Veterinary Test Laboratory, 31770 Colomiers, France; corine.boucraut@scanelis.com; 10 Department of Small Animal Diseases with Clinic, Institute of Veterinary Medicine, Warsaw University of Life Sciences-SGGW, 02-787 Warsaw, Poland; tadeusz_frymus@sggw.edu.pl; 11 Fundació Hospital Clínic Veterinari, Universitat Autònoma de Barcelona, Bellaterra, 08193 Barcelona, Spain; albert.lloret@uab.es; 12 Faculty of Veterinary Medicine, Università Degli Studi di Teramo, 64100 Teramo, Italy; fmarsilio@unite.it; 13 Dipartimento di Scienze Veterinarie, Università di Messina, 98168 Messina, Italy; mariagrazia.pennisi@unime.it; 14 Veterinary Virology and Animal Viral Diseases, Department of Infectious and Parasitic Diseases, FARAH Research Centre, Faculty of Veterinary Medicine, Liège University, B-4000 Liège, Belgium; etienne.thiry@ulg.ac.be; 15 Institute of Virology, Department for Pathobiology, University of Veterinary Medicine, 1210 Vienna, Austria; karinmoestl@gmail.com; 16 LMU Small Animal Clinic, Centre for Clinical Veterinary Medicine, LMU Munich, 80539 Munich, Germany; hartmann@lmu.de

**Keywords:** feline coronavirus, FIP, FCoV, mutation, diagnosis, treatment, antiviral

## Abstract

Feline coronavirus (FCoV) is a ubiquitous RNA virus of cats, which is transmitted faeco-orally. In these guidelines, the European Advisory Board on Cat Diseases (ABCD) presents a comprehensive review of feline infectious peritonitis (FIP). FCoV is primarily an enteric virus and most infections do not cause clinical signs, or result in only enteritis, but a small proportion of FCoV-infected cats develop FIP. The pathology in FIP comprises a perivascular phlebitis that can affect any organ. Cats under two years old are most frequently affected by FIP. Most cats present with fever, anorexia, and weight loss; many have effusions, and some have ocular and/or neurological signs. Making a diagnosis is complex and ABCD FIP Diagnostic Approach Tools are available to aid veterinarians. Sampling an effusion, when present, for cytology, biochemistry, and FCoV RNA or FCoV antigen detection is very useful diagnostically. In the absence of an effusion, fine-needle aspirates from affected organs for cytology and FCoV RNA or FCoV antigen detection are helpful. Definitive diagnosis usually requires histopathology with FCoV antigen detection. Antiviral treatments now enable recovery in many cases from this previously fatal disease; nucleoside analogues (e.g., oral GS-441524) are very effective, although they are not available in all countries.

## 1. Introduction

Feline coronavirus (FCoV) is a ubiquitous RNA virus present in many cat populations around the world. FCoV is primarily an enteric virus, and infection does not usually result in clinical signs or causes only enteritis [1,2,3,4,5] or failure to gain weight normally [3]. However, a small proportion of FCoV-infected cats go on to develop a serious disease mediated by a vasculitis [6], called feline infectious peritonitis (FIP). Coronaviral genomes possess a high level of genetic variation due to the error rate of RNA polymerase, leading to multiple mutations. Although FCoV infections can undergo a systemic phase within monocytes in healthy cats [7,8], mutations occurring in an individual cat are believed to allow a switch of cell tropism from enterocytes to monocytes to enable the subsequent development of highly pathogenic FIP-inducing FCoV [9], as discussed later in this review. However, an individual critical mutation has not been identified and likely does not exist [10].

FIP disproportionately affects pedigree cats [11,12,13,14,15,16,17] and those under two years old [11,13,15,18,19,20]. Most cases present with effusions (typically abdominal and/or pleural, occasionally pericardial, or scrotal) alongside fever, anorexia, and weight loss [17,18,20,21,22,23,24,25,26,27,28]. Abdominal lymphadenopathy is also reported [17,20,29,30], especially in cats without effusions [31]. Ocular (e.g., uveitis) [31,32] and neurological (e.g., ataxia) [31,33,34,35] signs can also occur.

Sampling an effusion, when present, for cytology, biochemistry, and FCoV antigen or FCoV RNA analysis is the most useful diagnostic step for FIP, while fine-needle aspirates (FNAs) from affected organs for cytology and FCoV RNA analysis are helpful if effusions are absent [36]. However, definitive diagnosis usually requires consistent histopathological changes in affected tissues with positive FCoV antigen immunostaining [22].

If prompt treatment with antivirals, typically the nucleoside analogue GS-441524, is not given, FIP has a very poor prognosis with a short survival time [27,37]. The recent development and availability of curative antiviral treatments [17,19,20,24,31,38,39,40,41,42,43,44,45] have revolutionised the approach to, and outcome of, FIP, although these treatments are often expensive and not legally available in all countries. Clinicians are now in need of diagnostic tools to help determine the likelihood of a diagnosis of FIP quickly [36] so that effective antivirals can be used as soon as possible in countries in which antivirals are available.

This extensive review is written by the European Advisory Board on Cat Diseases (ABCD), a scientifically independent board of experts from 11 European countries and gives a comprehensive update on the current state of knowledge on FIP and associated FCoV infection. The current guidelines are a major revision of the previous ABCD FIP guidelines, published in 2009 [46], and review the large body of research published in the field of FIP over the past 14 years. A little repetition is present in the sections of these guidelines as they have been designed to be readable in isolation, without needing to refer to other sections. However, the resulting guidelines are very long, and thus non-referenced boxed summaries are included at the end of each section (and subsections when needed) to provide an overview of essential facts in that area to allow access to information quickly.
**Summary of Section 1: Introduction:** This extensive review is written by the **European Advisory Board on Cat Diseases (ABCD)**, giving a **comprehensive update on feline infectious peritonitis (FIP) and feline coronavirus (FCoV) infection**. The guidelines have been written so that the different **sections are readable in isolation**, and these **non-referenced boxed summaries are included** to provide an easy-to-read overview of essential facts in that section.

## 2. Agent Properties

### 2.1. Virus Classification

Feline coronavirus (FCoV) [47] is a large, pleomorphic spherical, enveloped virus particle classified in the order *Nidovirales*; family *Coronaviridae*; subfamily *Coronavirinae*; genus *Alphacoronavirus*; species *Alphacoronavirus* 1, which also includes the enteritis-causing canine coronavirus (CCoV), transmissible gastroenteritis virus (TGEV) and porcine respiratory coronavirus (PRCoV) [48,49]. The newly emerged severe acute respiratory syndrome coronavirus 2 (SARS-CoV-2) is very distinct and different from FCoV, belonging to a different genus: the genus *Betacoronavirus* [50]. Separate guidelines on SARS-CoV-2 in cats are available [51].

### 2.2. Virus Genome and Structure

**Figure 1:**
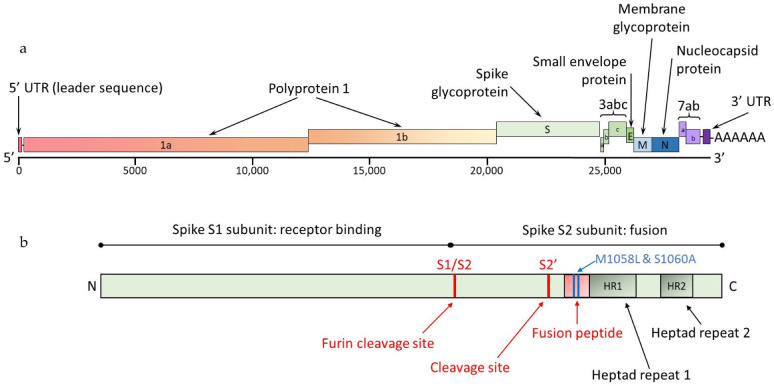
Schematic diagrams of type I FCoV, not drawn to scale. (**a**) Schematic FCoV genome. FCoV is a positive-sense single-stranded RNA virus. The FCoV genome of 27–32 kilobases encodes a replicase polyprotein, four structural proteins (spike [S], membrane [M], nucleocapsid [N] and envelope [E]) and non-structural accessory proteins 3a, 3b and 3c and 7a and 7b. UTR indicates an untranslated region. Image Emi Barker, Langford Vets, University of Bristol, UK [54]. (**b**) Schematic FCoV spike protein (based on [55,56,57]) sequence showing the division into the S1 and S2 subunits representing the receptor-binding and fusion domains, respectively, with N- and C-terminals shown. The S1/S2 and S2′ sites represent cleavage sites (in red), and the fusion peptide domain is also shaded in red. The positions of the M1058* and S1060* amino acid residues (blue lines) are shown because these correspond to the FCoV nucleotide sequences in specific spike gene mutations that are evaluated in some commercially available molecular assays. * Convention is to label amino acid substitutions by initials surrounding the numbered amino acid residue location (e.g., M1058L indicates that methionine is replaced by leucine at position 1058; similarly, S1060A indicates that serine is replaced by alanine at position 1060). Image Séverine Tasker, University of Bristol, UK.

Being an enveloped virus, FCoV is readily inactivated by most disinfectants, steam, and washing at 60 °C [52]. It has been suggested it might preserve its infectivity for days to a few weeks [53], depending on environmental conditions and protection by faecal matter. A schematic diagram of the FCoV genome is shown in Figure 1.

**Figure 2:**
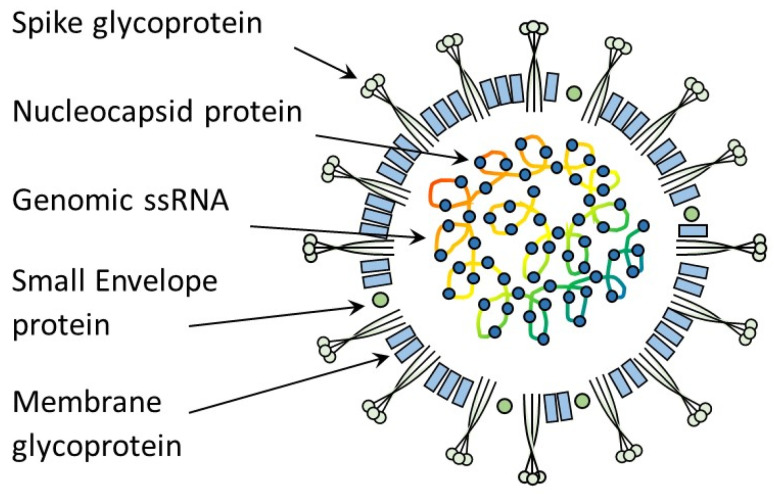
Schematic diagram of FCoV structure showing single-stranded (ss) RNA and the structural proteins: spike, envelope, membrane and nucleocapsid proteins. The spike protein is the part of the virus particle that interacts with the host-cell receptor. The spikes on the surface present a coronal (i.e., crown-like) appearance under electron microscopy [60]. Image Emi Barker, Langford Vets, University of Bristol, UK [54].

The 5′ two-thirds of the positive-sense coronavirus (CoV) genome consist of two overlapping open reading frames (ORFs), 1a and 1b, that encode non-structural polyprotein (pp) 1 (pp1a and pp1b) (Figure 1a). The polyproteins are cleaved into individual non-structural proteins (nsps), including RNA-dependent RNA polymerase that plays a role in viral replication. ORF 1a also encodes for viral proteases, including the viral 3C-like protease, which is a target for antiviral therapy (see Section 10 on Treatment of FIP). The other third of the genome consists of ORFs encoding structural proteins, a spike [S] (a protein found on the FCoV surface—see Figure 1b), a membrane (in the FCoV membrane), a nucleocapsid (the protein wrapped around the FCoV genome), an envelope (also in the FCoV membrane) (see Figure 2) and non-structural accessory proteins 3a, 3b, 3c, 7a and 7b (see Figure 1a) [58,59]. Non-structural proteins are involved in the replication of the virus and modification of the host immune response but are not incorporated into the mature virus particle. More information on the function of the structural proteins is found in Section 2.4 on FCoV pathotypes and genome mutations.

### 2.3. FCoV Types I and II and Replication

FCoV is divided into types I and II, based on growth in vitro, genomic properties, and antigenicity [61]. The biology of the two FCoV types (in particular with regard to receptor usage and cell culture adaptation) differs greatly with type II FCoV, although it is less common in the field, being the most easily isolated and grown in cell cultures in vitro [49].

**Figure 3:**
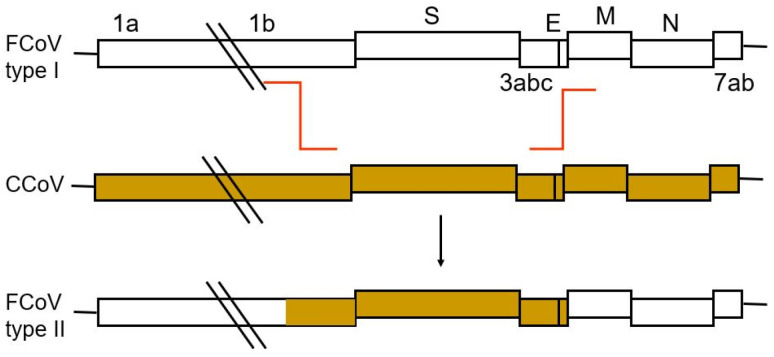
Origin of feline coronavirus (FCoV) type II; Image Peter Rottier, University of Utrecht, The Netherlands. Schematic diagram showing how type II FCoV arises from recombination of FCoV type I (shown in white) with CCoV (shown in brown).

Type II FCoV strains arise from recombination between type I FCoV and CCoV (Figure 3), usually including the spike of CCoV, varying amounts of adjacent 3a, 3b and 3c genes, and envelope genes, but not the nucleocapsid gene, which remains of FCoV origin [58,62,63]. Both type I and type II FCoV can occur as less-virulent FCoV and as FIP-associated FCoV [9]. Type I FCoV is more prevalent in most parts of the world [16,58,64,65,66,67,68,69,70,71,72,73,74]; a prevalence of type I of 80–95% has been reported [75,76].

Most laboratory research has focused on type II FCoV strains since they, unlike type I FCoV, can be readily propagated in vitro [77] (facilitating experimental studies), despite most field infections being type I FCoV. Experimental studies have tried to develop culture methods for type I FCoV using both permanent feline intestinal epithelial-cell cultures of ileocyte and colonocyte origin [78] and colonic organoid preparations [79], but neither are currently routinely available for use.

The RNA-dependent RNA polymerase makes a full-length negative-strand RNA copy of the genome as well as a nested set of smaller subgenomic RNAs with a common 3′ end [80]. These negative-strand RNAs serve as templates for new positive-sense genomes and positive-sense subgenomic mRNAs. The subgenomic mRNAs have a nested-set structure with sequences starting at the 3′ terminus and extending to various distances toward the 5′ end. If a real-time reverse-transcriptase polymerase chain reaction (RT-PCR) assay is designed to amplify 3′ subgenomic mRNAs, this can influence the quantitative results for apparent FCoV load [54] (see Section 7.5.2 on Detection of FCoV RNA by RT-PCR). In general, only the 5′ most ORF of each subgenomic mRNA is used for encoding the proteins, even though the subgenomic mRNAs have more than one coding sequence (except the smallest one).

### 2.4. FCoV Pathotypes and Genome Mutations

FCoVs have been assigned to two pathotypes (biotypes) [81,82], which can be referred to as feline enteric coronavirus (FECV), which mainly replicates in the enteric epithelial cells, and feline infectious peritonitis virus (FIPV), which results in a mostly lethal infection with efficient systemic replication in monocytes or macrophages [54,82]. Both types I and II can exist as each pathotype [82]. However, it is not only FIPVs that can replicate systemically, as FECVs have also been shown to replicate systemically in healthy cats and those without FIP [83,84,85]. In this review, we use the taxonomic term FCoV (as defined in virus nomenclature [47]), but distinguish viruses as a ‘less-virulent FCoV’ and an ‘FIP-associated FCoV’ when needed, to stipulate differences in biological behaviour between the two FCoV pathotypes.

FCoV genomes, like all coronaviral genomes, possess a high level of genetic variation due to the high error rate of RNA polymerase leading to different types of mutations, including point mutations, deletions, introduction of stop codons and recombinations [62,86,87,88,89].

The widely accepted hypothesis is that genetic variation and subsequent selection facilitate the switching of cell tropism from enterocytes to systemic monocytes/macrophages within an FCoV-infected cat that develops FIP [9]. This occurs when a less-virulent FCoV converts to an FIP-associated FCoV [90,91,92] via the so-called ‘internal mutation’ theory. This ‘internal mutation’ theory is supported by several studies showing a close genetic relationship between the FIP-associated FCoV and FCoV from faecal samples of cats without FIP living in the same environment [67,90,93,94], which is much closer than their relationship to FCoV collected from cats of other environments. However, the theory was questioned based on the results of a study that indicated that ‘FECV’ and ‘FIPV’ (the terms used in the study) were two distinct types of FCoV circulating independently in the cat population [95], leading to the ‘circulating virulent and avirulent FCoV’ theory. However, in that study, samples were derived from a population of shelter cats, a population in which the introduction of different genetically unrelated FCoV can be expected because of their different geographic origin [9]. One other study has provided some support for the ‘circulating virulent and avirulent FCoV’ theory in a small outbreak of FIP in shelter cats [55]. This ‘circulating virulent and avirulent FCoV’ theory may better explain the occasional FIP outbreaks reported in multi-cat environments [55,58,90,96,97,98].

Although the genes involved in the FCoV virulence genetic shift are still unknown, mutations in different genes have been postulated to be associated with the switch of the less-virulent (primarily intestinal) FCoV into the virulent (primarily systemic) FIP-associated FCoV, including the spike gene and accessory genes 3c and 7b [82,99] (see Figure 1b and Figure 2).

The spike protein comprises two subunits, S1 and S2 (Figure 1b); feline host-receptor recognition is mediated by S1 and membrane fusion by S2 [10,100,101]. The main receptor for type II FCoV is feline aminopeptidase N (fAPN) [102,103], but the main receptor for type I FCoV remains unknown [102,104]. Following receptor recognition [105], the spike protein is activated by feline host-cell proteases, such as furin [56]. Type I FCoV has two cleavage sites, called S1/S2 and S2′; the S1/S2 is cleaved by furin and referred to as the furin cleavage site [56]. Type II FCoV contains only the S2′ site [56]. Viral-cell-membrane fusion then occurs via the S2 subunit fusion domain [49]. As well the fusion domain, the S2 subunit (Figure 1b) contains two heptad repeats (HR1 and HR2) areas, also involved in viral membrane fusion [101].

Two alternative amino acid differences in the S2 fusion domain of the S protein, namely M1058L and S1060A (nomenclature based on position and nature of the amino acid change, i.e., methionine [M] to leucine [L] at position 1058 and serine [S] to alanine [A] at position 1060) (Figure 1b), have been detected. Together they distinguished FIP-associated FCoV in tissues from less-virulent FCoV in faeces [67,91,94], suggesting they were likely to be associated with the development of FIP. However, other studies [106,107], evaluating both faecal and tissue samples from cats with and without FIP, found these mutations in the viral genomes detected in the tissues of cats without FIP, suggesting their association with systemic FCoV infection, rather than FIP *per se*. A novel mutation (M1058F, where F represents phenylalanine) in this region has also been reported in association with FIP [94]. Clearly, the situation is complex, and it is likely, as has been suggested [10], that multiple mutations are involved in the development of FIP. More information on spike gene mutations is found in Section 7.5.3 on Molecular Techniques Characterising FCoV Spike (S) Gene Mutations following Positive RT-PCR for FCoV RNA.

The furin cleavage site (S1/S2) at the junction of the receptor binding (S1) and the fusion (S2) subunits of the spike protein, is another genomic region associated with FIP [108]. While all less-virulent FCoV had a conserved furin cleavage site, in most FIP-associated FCoV at least one substitution was found [108]. Other mutations in the S1/S2 cleavage site have been reported [55,56,109,110].

Mutations in the HR1 region of the S gene [87,111] have been said to be associated with FIP.

The ORF 3 gene encodes for a protein for which the exact function is still unknown. Interestingly, mutations leading to a truncated protein were detected in (up to approximately two-thirds of) the 3c genes of FCoV found in tissues of cats with FIP [86,92,112,113], while the ORF 3 gene was intact in all FCoV detected in faecal samples. This suggests that an intact 3c is an absolute requirement for the infection of the gut epithelial cells [99,112], but is not necessary for replication in monocytes. No association between 3c sequences and FIP was found in one extensive study [10].

Research has also evaluated the non-structural glycoprotein 7b, encoded by ORF 7b, for an association with FCoV virulence. Some suggested an association was present [114], whilst others disputed this [86,115].

There is no evidence that specific mutations in the 3a, 3b and 7a genes mediate the development of FIP [87]. It has been shown that ORF7b deletions occur readily in vitro, correlating with loss of virulence [116]

One novel study [10] evaluated natural selection differences between less-virulent FCoV and FIP-associated FCoV using molecular evolutionary genetic statistical techniques, focusing on the S, ORF3abc and ORF7ab genes. It found that there were two sites that showed differences in natural selection pressure between less-virulent FCoV and FIP-associated FCoV,—one within the S1/S2 furin cleavage site and the other within the fusion domain of S. The authors deduced that a combination of mutations in non-pathogenic FCoV likely contributes to FIP development and that it was unlikely to be one singular ‘switch’ mutational event [10].
**Summary of Section 2: Agent Properties:** Feline coronavirus (FCoV) is the causative agent of the serious disease of feline infectious peritonitis (FIP). FCoV is a large, pleomorphic spherical, **enveloped virus** particle with a single-stranded **RNA genome**. It is **readily inactivated** by most disinfectants. Being an RNA virus, FCoV has a high level of genetic variation due to frequent errors **(mutations) during RNA replication**. The hypothesis is that genetic variation and subsequent selection facilitate the **switching of cell tropism from a mostly mild enteric** (less-virulent) **FCoV pathotype** to an **FIP-associated FCoV pathotype**. This switch occurs in an infected cat and FIP-associated **FCoV systemically replicates efficiently within monocytes/macrophages** and can lead to the serious disease of FIP. **However, systemic (non-enteric) FCoV infection can also occur in cats without FIP**. The FCoV genome comprises many genes, including those encoding the spike [S], matrix, nucleocapsid, envelope proteins and non-structural accessory proteins 3a, 3b, 3c, 7a and 7b. **Mutations in different genes**, including the **S gene**, have been postulated to be associated with the **switch to a more virulent FCoV pathotype**. The S protein is a particular focus of attention as it mediates entry into feline host cells and has both receptor-binding and fusion functions. Specific mutations in the S gene have been postulated to be associated with FIP-associated FCoV but the definitive genes and mutations involved in the FCoV virulence genetic shift are still unknown. **Type I and type II FCoV are recognised to differ based on antigenic and genomic properties, with type I FCoV being more prevalent**. However, type I FCoVs, unlike type II FCoVs, are difficult to grow in cell cultures and thus many in vitro studies are based on the less-common type II FCoV. Type I and II FCoV can both exist as less-virulent FCoV and FIP-associated FCoV.

## 3. Epidemiology

### 3.1. Transmission of FCoV

FCoV is a contagious virus. Faeces are the main source of FCoV infection, with litter trays representing the principal source of infection in groups of cats. Cats are most likely to be infected orally, with transmission being mainly indirect following contact with objects contaminated with faeces (e.g., via litter trays, cat litter fomites and scoops, brushes, vacuum cleaners, shoes) and by grooming paws contaminated during litter tray use. Thus, the major route of transmission is faecal-oral.

A case report [117], documenting FIP-associated rhinitis, suggested that the respiratory tract might be a place of entry for the transmission of FCoV. Since the virus is found only rarely in the saliva of healthy cats, close contact or sharing feeding bowls are not major routes of infection [5].

Transplacental transmission has been described from a queen that developed FIP during pregnancy [118], but this phenomenon is extremely rare [119]. A study [120] evaluated testicular tissue and semen for FCoV RNA by RT-PCR in male cats to evaluate the risk of venereal transmission of FCoV. FCoV RNA was amplified from around 15% (6 of 39) of testicles in the study and none of the 17 semen samples tested, suggesting venereal transmission of FCoV would be unlikely.

The transmission of FCoV via blood transfusion has not been reported.

The possibility of mechanical vectors being involved in the transmission of a highly virulent strain of FCoV has been suggested during the early investigation of a large outbreak of FIP in Cyprus [98], similar to discussions around the transmission of SARS-CoV-2 by cat fleas [121]. However, further research is required to confirm this, and vector transmission for FCoV has not yet been confirmed.

In FCoV-infected breeding catteries, kittens commonly become infected when young, within a few weeks of age [122] (see also Section 4 on Pathogenesis and Section 5 on Immunity). Results from multivariable analysis suggested that young age (less than one year) was significantly associated with FCoV shedding in one study of German breeders [123], but in another study, evaluating a similar group of cats from these breeders, age was not significantly associated with FCoV shedding [124].

After natural infection, cats begin to shed the virus in the faeces in as early as two days [85,125] and continue to shed for days, weeks, months, and a few even for life (persistent infection) [5,59,64,85,125,126,127]. Shedding typically lasts a few weeks to months, then stops, or occurs intermittently, and can recur due to re-infection in an endemic environment, as immunity is short-lived [5,7,64,85,94,126,128,129,130,131]. In breeding catteries in one study [124], 19% of cats were categorised as being intermittent shedders, with the variable detection of FCoV RNA in four faecal samples collected at intervals of between 5 and 28 days. In another study of pet cats, 31% were deemed to be either intermittent shedders or to have recovered and then been re-infected [5]; this study was unique in terms of the very long (up to five years) follow-up of the cats.

However, it has been suggested that the true intermittent shedding of FCoV does not occur, but a cat can appear to intermittently shed due to the following [132]:

- Cycles of re-infection.
- Faecal FCoV RNA levels around the limit of detection of the RT-PCR assay being used such that positive and negative results occur interchangeably.
- The presence of faecal or cat litter RT-PCR inhibitors affecting RT-PCR sensitivity, giving false-negative results.

A few cats (3–9%) never shed FCoV following infection [5,128]; these cats may be resistant to FCoV infection. In the study of breeding catteries [124], 24% of cats were negative for FCoV RNA in all four faecal samples collected at intervals of between 5 and 28 days; it is not known if these cats were resistant to infection, as they were only followed for around four months and could have shed FCoV before testing started or after it was stopped.

Some cats develop persistent virus shedding; around 13% in natural infection (with positive FCoV shedding identified for at least eight consecutive months) [5] and 22% in experimental infection [126]. However, a standard definition of a persistent FCoV shedder cat does not exist. One method involves testing four faecal samples, each one week apart, as this resulted in the same identification of FCoV shedders as samples collected weekly for 24 weeks [59,133]. In a study of breeding catteries [124], cats were regarded as being persistent shedders if they gave positive results for FCoV RT-PCR on at least three of the four faecal samples collected from each cat in the study; using this definition, 56% were deemed to be persistent shedders, with the majority (89%) of these cats giving positive results on all four faecal samples. Such cats are likely to play a major role in the transmission of FCoV within households.

Persistent virus shedding may be influenced by the dose of virus received at inoculation [131], although in one study of naturally infected cats, the virus was remarkably conserved over a period of years, suggesting that it had found an antigenic niche not detectable by the host’s immune system [64].

Faecal excretion reaches high levels, especially in kittens [5,126,131]. The higher the FCoV antibody titre, the greater the chance of the cat shedding FCoV [5,122,126,134,135], as well as the greater the frequency of faecal FCoV shedding, and the higher the FCoV virus load present [135]. The identification of patterns of faecal shedding based on RT-PCR will rely on, in part, the sensitivity of the RT-PCR being used to detect the FCoV RNA, as mentioned above, and the frequency of faecal RT-PCR testing. Due to the suspected short duration of any immunity following infection, failure to separate out cats that are FCoV shedders could favour the spread and persistence of FCoV in a household [59], which could account for the high antibody prevalence in the multi-cat environment.

The horizontal transmission of FIP-associated FCoV, in contrast to less-virulent FCoV, is believed to occur only rarely (see Section 4 on Pathogenesis, Section 5 on Immunity, and Section 8.1 on Does a Cat with FIP Pose a Threat to Other Cats in its Household?), such as in shelters, as proposed in the ‘circulating virulent and avirulent FCoV’ theory. Indeed, horizontal transmission has been described as ‘the exception rather than the rule’ [59]. FIP can be induced experimentally by the inoculation of an FIP-associated FCoV intraperitoneally [136]—a route that bypasses the natural faecal-oral transmission pathway.

Although FCoV and CCoV are closely related, contact with dogs does not appear to be a major predisposing factor for CoV infection in cats [63]. However, one study [137] found feline/canine CoV recombinant viruses in cats of a rescue shelter that housed both cats and dogs. In the M protein gene, these strains were more closely related to FCoV-like CCoV than to FCoV, suggesting that infection with CCoV and subsequent recombinations with FCoV had occurred within this environment.

### 3.2. Prevalence of FCoV

With the exception of a few islands of isolated feline populations (e.g., the Falkland Islands) [138,139,140], FCoV infection has been reported worldwide. FCoV, and therefore FIP, is particularly common where conditions are crowded [135,141] and less common in individually housed, stray or feral cats [3,142,143,144,145,146,147,148]. In one study using multivariable analysis [148], cats adopted as strays were more likely to be FCoV antibody-positive, as were cats that had contact with other cats. Wild felids, especially those in zoos, can also be FCoV-infected [149]. FCoV-infected cheetahs are even predisposed to develop FIP [150].

FCoV is highly contagious, and in households where it is present, the prevalence of serum FCoV antibodies indicating exposure is often high (see Section 5 on Immunity).

Cats who spent over 60 days in UK shelters were five times more likely to have FCoV antibodies than the same population on the day of entry to the shelter [143]. This may be due to increased transmission and exposure within shelters, but the stress of admittance to a shelter may also play a role in the increased FCoV antibody prevalence.

In an Italian study [147] using multivariable analysis, domestic shorthair cats were less likely to be FCoV antibody-positive compared to some pedigree breeds.

In a Japanese study including 17,392 cats, the FCoV antibody prevalence was 67% in purebred cats and 31% in non-pedigrees [146]. In purebred cats, seroprevalence increased rapidly in early life, reaching around 80% by three months of age, and remaining at this level until around two years of age. Seroprevalence thereafter progressively decreased, reaching around 30% in cats aged 14 years or more. In contrast, in the non-pedigree cats, there was little fluctuation in seroprevalence, with levels remaining at around 30% at all ages. The authors suggested that this could be due to the multi-cat environments that the pedigree cats were likely to be kept in, leading to the high seroprevalences in younger cats [146]. Among the purebred cats in this study in Japan, the American shorthair, Himalayan, Oriental, Persian, and Siamese breeds showed low antibody prevalence, while the American curl, Maine coon, Norwegian Forest cat, Ragdoll and Scottish fold breeds had high antibody prevalence [146].

In a German study of breeding catteries [124], the Persian breed was associated with persistent high FCoV shedding (i.e., FCoV RNA detection in faeces), whereas the Birman and Norwegian Forest breeds were more likely to be non-FCoV shedders. It is not known if these results were due to genetic susceptibility or resistance, or whether they were related to husbandry factors within those breeds’ households.

It has been found that the feline AB blood group phenotype is not associated with FCoV antibody-positive status, i.e., there is no association between blood types A, B or AB and FCoV antibody presence [147]. Other feline blood groups, such as Mik, have not been investigated.

The prevalence of FCoV, determined by the detection of FCoV antibodies and/or FCoV RNA in faeces, in studies from various countries, is given in Table 1.

### 3.3. Prevalence and Risk Factors for FIP

The prevalence of FIP within a cat population as a whole was 0.5% (60 of 11,535) of all the cats examined at the North Carolina State University College of Veterinary Medicine (1986–2002), a tertiary referral centre [11]. A retrospective database study of 24 American veterinary teaching hospitals revealed a diagnosis of FIP in 1420 cats from 397,182 (0.4%) feline consultations over a 10-year period [13]. The percentage of FCoV-infected cats that develop FIP is small (usually less than 10%), but it is variable in different studies and populations [36]. In one study, FIP mortality in 282 FCoV antibody-positive kittens was 8% [167]. The incidence of FIP in a household or cattery increased with the number of cats present in one study [168] but was not associated with mean cat number in another [169]. A seasonal variation has been noticed, with the lowest number of recorded FIP diagnoses in July and increased diagnoses in January to April (winter) in one study [13], and an increased number in autumn and winter in another [169]; both of these studies were derived from data collected from the Northern Hemisphere.

FIP disproportionately affects pedigree cats [11,12,13,14,15,16,17] and those under two years of age [11,13,15,18,19,20]. In some studies, cats less than one year of age were particularly represented [13,14,16,17,20,27]. In Germany, 39% of 222 cats with FIP were under 1 year old, and thereafter, the age of cats with FIP was evenly distributed except between 7 and 11 years of age, when the incidence was about half that of other age groups [18]. 

In a North Carolina study [11], pedigree cats were also over-represented for FIP; FIP was present in nearly 1.3% of the pedigree cats compared to 0.35% in mixed breed cats, and breed predisposition was statistically significant in the Abyssinian, Bengal, Birman, Himalayan, Ragdoll and Rex breeds. In Australia [14], 71% of cats with FIP were purebred [15], and in a different Australian study, domestic crossbreeds and Persian and Himalayan cats were significantly under-represented in the FIP cohort, while several other breeds were over-represented, including British shorthair, Devon Rex and Abyssinian.

The percentage of effusions that were found to be positive by FCoV RT-PCR varied with the cat’s breed and age in a Japanese study [16] and with age in a study in China [68]. In Japan, 210 (56%) of 377 FCoV RNA RT-PCR-positive ascitic samples were from cats of one year of age or less [16], and in the Chinese study, only 1 of 127 cats with suspected FIP was over 7 years old [20]. In the Japanese study, in cats up to one year of age, 95% of effusions of pedigree cats were RT-PCR-positive compared to 79% of the effusions of non-pedigree cats [16]. However, in these studies, FIP was not confirmed as a diagnosis; the study used a positive FCoV RT-PCR result on an effusion to indicate that a diagnosis of FIP was likely.

Some authors have noted a predisposition for FIP in male over female cats [13,14,15,16,17,18,76], while others found no sex predisposition [20,170,171], although neutered status was associated with FIP in one study [20]. Pedigrees of cats that die of FIP can often be traced back to the stud cat, rather than the queen [172], but, unexpectedly, breeding intentionally for FIP resistance led to more, rather than less, FIP occurring in the offspring [173], which is of note. In one study of multi-cat households [169], the number of cats in the household was not associated with the development of FIP, but the number of cats shedding FCoV, as well as the proportion of cats that were chronically shedding FCoV, were associated with FIP. Occasionally, there are reports of several littermates all developing FIP, possibly suggesting a genetic predisposition in those siblings [174].
**Summary of Section 3: Epidemiology:** FCoV is a contagious virus. Faeces are the main source of FCoV infection and most **transmission is faecal-oral in nature**. **Kittens are often infected at a young age** and **shed FCoV in faeces as early as two days post-infection**. After infection, **shedding continues for days, weeks or months**, and a few may be persistently infected. Shedding then stops, or is detected intermittently, and **can recur due to re-infection in an endemic environment**. Immunity is short-lived, which is why cats, in the face of infection, can undergo multiple cycles of infections. FCoV infection occurs worldwide (see Table 1) and is very common, particularly in multi-cat households, **but FIP arises in only a small percentage of FCoV-infected cats**. Cats of any breed or age can develop FIP. It is particularly seen in **pedigree cats** (especially in certain breeds in some studies) and those **under 2 years of age**. In some studies, males were more likely to develop FIP than females.

## 4. Pathogenesis

As noted above, the major route of FCoV infection is faecal-oral. Following ingestion, the virus first enters and replicates within the epithelial cells of the small intestinal villi [1]. Type II FCoV uses the fAPN present on the intestinal villi and the monocytes [102,103], whilst the receptor for type I FCoV remains unknown [102,104].

FCoV shedding occurs in the faeces from as early as two days post-infection [7,85,125]. This infection is not usually associated with clinical signs but sometimes can be accompanied by enteritis [1,2,3,5,175] and/or upper respiratory tract signs [119]. Occasionally, very severe, indeed fatal, coronavirus enteritis has been reported [4]. As described earlier, the virus shedding of type I FCoV in faeces can follow different patterns [130]. Most cats infected with type I FCoV shed the virus for two to three months [5], either continuously or possibly intermittently [132], and then stop; immunity is short-lived because these cats can be re-infected by the same or different strain of FCoV within a few weeks [64]. Conversely, around 13% of cats infected with type I FCoV become persistently infected carriers [5] and shed FCoV in their faeces persistently. However, cats experimentally infected with type II FCoV shed the virus for around two weeks [125], and no type II carrier cat has been reported yet.

Fortunately, only a small proportion of FCoV-infected cats go on to develop FIP [6,167,176].

From two weeks post-infection, the virus is found in the colon [7]. In persistently infected carrier cats without clinical signs, the ileum, and especially the colon, are the main sites of persistent viral replication [7,127].

The mesenteric lymph nodes (MLNs), as the most likely first site of FCoV spread from the intestine regardless of subsequent viraemia, have been evaluated for mediators of the innate immune response, and evidence of toll-like receptor involvement has been found in the response to FCoV infection [177].

Efficient FCoV replication in activated monocytes and macrophages is a key event in FIP pathogenesis [178], governing:

- Whether or not the cat will go on to mount a successful immune response and eliminate the virus.
- Whether the cat will mount a semi-successful immune response, remaining clinically well, but shedding FCoV in the faeces for months to years.
- Whether the cat will mount a deleterious immune response (sometimes the pathology is described as being immune-mediated in nature [59]), resulting in a widespread pyogranulomatous vasculitis and ultimately premature death without effective antiviral treatment.

The outcome of infection of the monocytes and macrophages is partially dependent on the host cell; however, virulent strains do replicate more efficiently within permissive monocytes and macrophages [179]. Monocytes from an outbred population of cats varied in their ability to sustain FCoV replication regardless of whether the strain of FCoV was deemed very virulent or relatively avirulent, with the monocytes of some cats not sustaining replication of either FIP-associated FCoV or less-virulent non-FIP-associated laboratory strains of FCoV [179]. What happens in monocytes and macrophages following FCoV infection in FIP is quite extraordinary: usually, an infected cell will display viral antigens in association with a feline leucocyte antigen (the feline version of the major histocompatibility complex) on its surface to enable antibody-mediated, or cell-mediated, lysis, but in cats with FIP, infected macrophages lack the surface expression of viral antigens, helping escape cell lysis [180].

FCoV viraemia, when it occurs, is short-lived, peaking about 7 to 14 days post-infection and declining thereafter [7,8]; thus, by the time clinical signs of FIP appear, viraemia cannot always be detected, and RT-PCR tests on blood samples to detect FCoV RNA have been negative. However, this pattern of negative RT-PCR results on blood samples in FIP has not been observed in recent studies, which have found that a high percentage of cats with FIP have detectable FCoV in their blood by RT-PCR at diagnosis [19,24,31] (see Section: FCoV RT-PCR on Blood Samples). This suggests that FCoV viraemia might last longer than previously thought.

The virulence of the virus, the viral load and the cat’s immune response determine whether or not FIP will develop. Thus, both viral genetics and host immunity are likely to play a role in the development of FIP [8,171,179,181,182,183,184]. Resistance, in terms of the ability of the host to ‘fight off’ FCoV infection, likely increases between 6 and 12 months of age [9], although FIP can occur at any age [18].

In those cats in which FCoV is able to replicate freely within the monocytes, the monocytes attach to the walls of small- and medium-sized veins, releasing matrix metalloproteinase-9, which destroys the collagen of the basal lamina of affected vessels [6]. This event permits the extravasation of the monocytes, where they differentiate into macrophages. The breakdown of the endothelial tight junctions allows plasma to leak out of the vessels [6]. It is believed that the death of virus-laden macrophages (apoptosis) plays a key role in FCoV dissemination [185]. In more acute forms of FIP, many blood vessels are affected, and this plasma leakage becomes apparent clinically as an effusion in the abdominal, thoracic and/or pericardial cavities. Within this process, the deposition of immune complexes and subsequent complement activation is thought to cause an intense inflammatory response that may extend across blood vessel walls, rendering them more permeable [59]. In more chronic forms of FIP, fewer blood vessels are affected, but the perivascular pyogranulomata on affected organs can become quite large and is even easy to mistake for a tumour upon gross examination, exploratory laparotomy or post-mortem examination. FCoV-infected macrophages release cytokines, such as tumour necrosis factor-alpha (TNF-α) [186], which upregulates fAPN [186], causes lymphopenia [187] and inhibits neutrophil apoptosis [188]. The role of TNF-α is important in the development of FIP, such that anti-TNF-α antibodies have been used as a possible therapy [189,190].

As described above, FIP arises only in a small percentage of FCoV-infected cats following FCoV infection, and the horizontal transmission of FIP via an FIP-associated FCoV strain is believed to be a very unlikely occurrence. Indeed, several experimental and field observations support the assumption that cats do not become infected with FIP-associated FCoV orally. FIP-associated FCoV strains from different cats of the same household show mostly unique genetic characteristics, suggesting that these viruses developed independently in individual cats [90,91,108]. Additionally, only a very small percentage of cats with FIP shed FIP-associated FCoV, most likely because the mutated viruses cannot replicate in enterocytes due to deletions in the 3c gene [92,96,99,107]. Furthermore, faecal samples of cats with FIP do not cause disease after oral inoculation [99]. Also, in multi-cat households, FIP cases are often limited to a single cat (or occasionally, at most, a few cats) and additional cases might not occur for several years.

However, a few reports exist in which a higher number of cats (greater than 10%) developed FIP in multi-cat environments [55,58,90,96,97]. Although such outbreaks (referred to as epizootics) are rare, they certainly occur. Several factors might contribute to these outbreaks, such as increased population stress (usually due to overcrowding or high kitten production), unintentional use of genetically predisposed cats, introduction of a new FCoV strain (such as one that has a high chance of becoming an FIP-associated FCoV) [98], or possible horizontal transmission [21], in line with the previously mentioned ‘circulating virulent and avirulent FCoV’ theory.

**Summary of Section 4: Pathogenesis:** **FCoV infection occurs following the ingestion of the virus**, which then replicates in the **epithelial cells of the small intestinal villi,** resulting in **faecal shedding**. This **enteric FCoV infection is often subclinical but can result in enteritis**. FCoV is then found in the **colon**, which is the main site of viral replication alongside the **ileum**. Thereafter, FCoV infection is thought to spread to the **mesenteric lymph nodes** before sometimes resulting in **viraemia**. Whilst low-level FCoV viraemia in monocytes can occur in cats that do not go on to develop FIP, **efficient and high-level FCoV replication in activated monocytes and macrophages** (which may well be mediated by viral mutations) is believed to be a **key event in FIP pathogenesis**, alongside the nature of the immune response mounted by the cat in response to FCoV infection. When FIP develops, there is a reaction between replicating FCoV in monocytes and blood vessel walls, allowing the extravasation of the monocytes, where they differentiate into macrophages. **The breakdown of the endothelial tight junctions allows plasma to leak out of the vessels**; this can appear clinically as an **effusion in the abdominal, thoracic and/or pericardial cavities**. In more chronic forms of FIP, fewer blood vessels are affected, but **larger perivascular pyogranulomata result on affected organs**. **The horizontal transmission of FIP, via an FIP-associated FCoV strain, is believed to be a very unlikely occurrence.**


## 5. Immunity

The development of FIP is associated with the severe suppression of natural killer cells and regulatory T cells—central players in the innate and adaptive cell-mediated immunity (CMI), respectively [191]. Until the study on FCoV replication in monocytes was conducted by Dewerchin et al. [179], the outcome of FCoV infection had been mainly attributed to virulence factors (mutations, deletions) in the virus [9], although host factors obviously played a role in pathogenesis. An effective early T cell response is believed to critically determine the outcome of infection with FCoV [192].

One of the most investigated cytokines important in FCoV infection has been interferon (IFN) gamma (IFN-γ), which is an important modulator of CMI. The expression of IFN-γ mRNA by leucocytes in the circulation or in tissues has been investigated in many studies using RT-PCR and immunohistochemistry (IHC) [193,194,195,196,197]. Some studies [195,196,197] found high IFN-γ mRNA expression in the peripheral blood leucocytes of clinically normal cats with FCoV infection, but low expression in cats with FIP. In contrast, IFN-γ mRNA is abundant within FIP lesions [193]. Giordano et al. [198] concluded in their study that although cats resistant to FIP have strong CMI, which can be measured by high serum IFN-γ production, CMI is also likely to be involved in the pathogenesis of FIP, albeit at a tissue level, as evidenced by high IFN-γ concentration in FIP effusions. These findings could be the basis of further studies into the mechanisms through which IFN-γ production could prevent the onset of FIP. The importance of CMI in the resistance to FIP was further investigated in an experimental study [8] in which the antiviral T cell responses were measured during primary and secondary exposure to FIP-associated FCoV. Definitive adaptive T cell responses, predictive of disease outcome, were not detected during the early phase of primary infection with FIP-associated FCoV, but recovery antiviral T cell responses were seen later in primary infection for a subset of cats showing slow progression to FIP or resistance to FIP compared to those showing a fast progression of FIP. The emergence of antiviral T cell responses after secondary exposure (re-challenge) to FIP-associated FCoV in cats that were resistant to FIP after primary infection also suggested a role of CMI in the later control of infection with FIP-associated FCoV and disease progression.

Hsieh et al. [183] investigated whether single-nucleotide polymorphisms (SNPs) in the feline IFN-γ gene were associated with the outcome of FCoV infection. Some ‘FIP-resistant’ and ‘FIP-susceptible’ alleles were suggested, and a subsequent study found an increased frequency of documented feline IFN-γ SNPs in pedigree cats, but small numbers limited statistical analysis [199]. A larger study [200] published on the prevalence of feline IFN-γ SNPs in non-pedigree cats did find a statistical association between the presence or absence of FIP and genotype; however, the strength of this association (presence of the ‘protective’ genotype in 16% of the cats with FIP and its absence in 66% of the cats without FIP) limits its use in individual cats or to guide breeding. Another study found associations between FIP and SNPs in the TNF-α and the dendritic cell-specific intercellular adhesion molecule-grabbing non-integrin (DC-SIGN) genes [201], although no such associations were found in a subsequent study [199].

The role of humoral immunity in protecting against FIP is ambiguous. Maternally derived antibodies have been suggested to provide protection until about five to six weeks of age [3] until they decline and become undetectable by six to eight weeks of age. However, infection at two weeks of age has also been detected rarely [122], questioning protection by maternally derived antibodies. On the other hand, cats with active enteric FCoV infections have strong systemic IgG and mucosal secretory IgA responses that wane after FCoV clearance, with no evidence of a mucosal IFN-γ T cell response, suggesting that humoral responses can control infection [202].

Seroconversion (i.e., antibody production) to FCoV takes 7 to 28 days post-infection [59,85,131,203]. Following natural infection, antibody titres can decline to zero over a period of several months to years, as demonstrated by more than half the serum antibody-positive cats in 24 of 73 households with endemic FCoV infection becoming serum antibody-negative [167]. In other longitudinal studies of multi-cat households [64], FCoV antibody titres were variable (i.e., increased and decreased), believed to be due to cycles of infection and re-infection, but they can decrease when maintaining closed households [204] or with the segregation of serum antibody-positive and -negative cats [5].

The clearance of natural infections has been associated with antibodies directed against the FCoV S protein [205]. Conversely, in experimental infections, antibodies directed against the S protein can be detrimental [206]. In cats with pre-existing antibodies, ‘antibody-dependent enhancement’ (ADE) has been observed experimentally, resulting in a more rapid disease course and earlier death [59]. This enhancement was observed irrespective of whether cats had acquired antibodies through passive or active immunisation using experimental vaccines [206,207,208]. However, in field studies, cats developed FIP on first exposure to FCoV (and thus did not have pre-existing antibodies), and some cats experienced repeated FCoV infections without developing FIP, leading to the conclusion that ADE is likely an experimental phenomenon, which is not believed to be important in natural infection [64,167,209]. Additionally, an experimental study [8] documented that 9 of 10 cats that had not developed FIP following primary infection with an FIP-associated FCoV strain resisted the development of the disease following re-challenge. However, the phenomenon of ADE still remains a major concern in vaccine development for FIP.

**Summary of Section 5: Immunity** Cats **resistant to FIP** are known to have **strong cell-mediated immunity (CMI)**, which can be measured by high levels of the cytokine interferon gamma (IFN-γ) in the serum. However, CMI is also likely to be involved in the pathogenesis of FIP, albeit at a tissue level, as evidenced by high IFN-γ concentration in FIP effusions. Single-nucleotide polymorphisms (SNPs) in the feline IFN-γ gene have been found to be associated with the outcome of FCoV infection, but these associations are not discriminatory enough to be beneficial to deduce susceptibility in individual cats, nor to guide breeding. The **role of humoral immunity in protecting against FIP is ambiguous**. Maternally derived antibodies are thought to provide protection until kittens are about five to six weeks old, until they decline by six to eight weeks of age. **Antibody development to FCoV takes 7 to 28 days post-infection**. Following natural infection, antibody titres can decline to zero over a period of several months to years. In cats with pre-existing antibodies, **‘antibody-dependent enhancement’ (ADE)** has been observed experimentally, resulting in a more rapid FIP progression and earlier death. However, in field studies, cats developed FIP on first exposure to FCoV (and thus did not have pre-existing antibodies), and some cats experienced repeated infections by FCoV and did not develop FIP, leading to the conclusion that ADE is likely to be an experimental phenomenon, but it still remains a concern for vaccine development.

## 6. Clinical Signs

### 6.1. Clinical Signs Associated with FCoV Infection

FCoV infection does not often cause clinical signs sufficient for a cat owner to seek veterinary attention following infection, although FCoV-infected littermates tend to have poorly grown kittens amongst them and a more frequent history of diarrhoea and upper respiratory signs than uninfected kittens [119]. Occasionally, FCoV infection causes enteritis [1,2,3,5,132,175] with clinical signs of diarrhoea and/or vomiting. FCoV infection was significantly associated with diarrhoea in a study of 234 cats from 37 breeding catteries in Germany, although faecal FCoV load was not correlated with faecal consistency scoring [154]. Although co-infections with potential enteropathogens were also common in this study, their presence in cats with FCoV infection was not associated with diarrhoea [154]. FCoV infection was also significantly associated with diarrhoea in cats from home-based foster care, but not in cats from shelters, sanctuaries, or trap-neuter-return programs in the USA [210]. Occasionally, very severe, even fatal, coronavirus enteritis has been reported [4], and chronic diarrhoea was reported in FCoV carrier cats [5,132].

### 6.2. Clinical Signs Associated with FIP

#### 6.2.1. General Clinical Signs of FIP

The clinical picture of FIP varies considerably, reflecting the variability in the distribution of the vasculitis and (pyo)granulomatous lesions. The vasculopathy can result in effusions (‘wet FIP’), whilst granuloma formation alone results in ‘dry’ or ‘non-effusive FIP’ mass lesions. The clinical presentation that includes the development of effusions is regarded as being most common [18,20,21,22,23,24,25,26,27,45]: 78% of 224 cases of FIP had effusions [18] in one study. The distinction between so-called ‘effusive’ and ‘non-effusive’ forms of FIP is important for diagnostic purposes because the analysis of an effusion is so useful. However, there is a considerable overlap between the two forms and, indeed, FIP cases with effusions also have pyogranulomatous lesions visible at post-mortem examination or can evolve to a more non-effusive disease, and, similarly, cats without effusions can develop effusions [43]. Clinical signs of FIP can also change over time, and therefore repeated physical examinations are important to detect newly apparent clinical signs; for example, an effusion can develop, or ocular changes can become visible on ophthalmoscopic examination. ABCD FIP Diagnostic Approach Tools [211] are available to help the veterinarian assess clinical signs for FIP.

Non-specific clinical signs can occur in both cats with effusions or without effusions and include fever, lethargy, anorexia and weight loss [45,212] (or failure to gain weight/stunted growth in kittens), although occasionally some cats remain bright and retain good body condition. Fever is commonly present, and it can fluctuate and is refractory to many drugs and non-responsive to antibiotics. One study describing referral cats with a history of fever found that FIP was the most common diagnosis made, highlighting its importance as a differential diagnosis for fever even at referral level [213]. Another study [18], which described the clinical features of FIP, documented fever in 56% of FIP cases. Fever was shown to be more common in cats with effusion than in cats without effusion [18].

**Figure 4:**
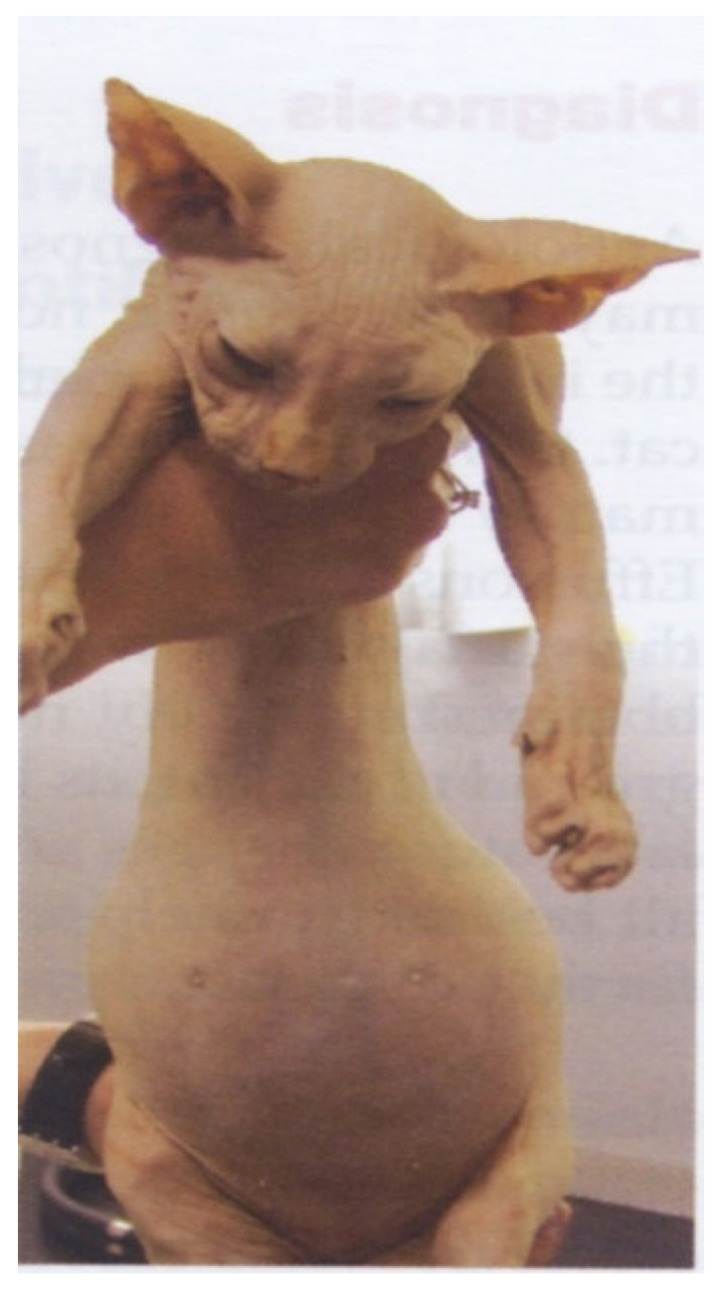
Ascites in a young Sphinx cat presenting with FIP. Image Hannah Dewerchin, Ghent University, Belgium [46].

FIP can be associated with effusion formation in one or more body cavities. Abdominal effusions leading to a clinical presentation of ascites, sometimes with abdominal distension, are the most common effusions seen with FIP [17,18,45] (Figure 4).

Pleural effusion can be present concurrently to abdominal effusion. In some cats, the effusion is restricted to the thorax; cats with pleural effusion can present with dyspnoea [21,45,213,214]. In a retrospective study [215] including 306 cats diagnosed with pleural effusion of established aetiology, FIP was only diagnosed in 9% of cats, while cardiac disease was the most common aetiology (35%), followed by neoplasia (31%), pyothorax (9%) and chylothorax (5%). Cats with FIP were significantly younger than those with cardiac disease and neoplasia, and cats with cardiac disease had a significantly lower body temperature, higher serum alanine aminotransferase and alkaline phosphatase activity, and lower protein concentrations and nucleated cell counts in the effusion than cats with FIP [215].

Pericardial effusions [28,216], with or without effusions in other body cavities, are also occasionally reported. Rarely, effusion in the scrotum is present in intact male cats due to a serositis involving the tunica vaginalis of the testes, leading to scrotal enlargement. When effusions form in FIP, the disease progression is often quite acute in nature, progressing within a few days or weeks and severely limiting survival [37].

FIP is often more difficult to diagnose when effusions are not present because fever, anorexia, lethargy, and weight loss (or failure to gain weight in kittens) can be the only clinical signs, particularly in the early stages of disease. FIP presenting without effusions also tends to be more chronic than FIP associated with effusions, progressing over a few weeks to months. Additional signs of FIP without effusions depend on the organs affected by the pyogranulomatous lesions and can include the central nervous system (CNS) [31,33,34,35], eyes [31,32] and/or abdominal organs (such as the liver, abdominal lymph nodes, kidney, pancreas, spleen and/or gastrointestinal tract) [15], and such signs can also occur in cats with effusions.

**Figure 5:**
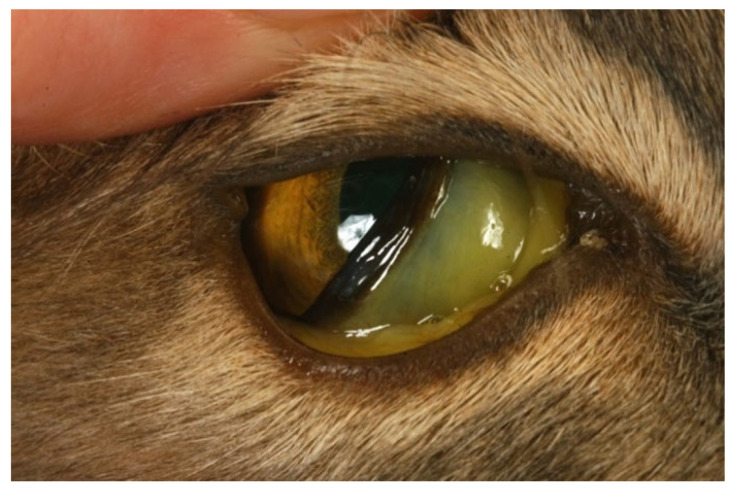
Icterus can occur in cases with FIP, particularly in cats with effusive FIP. Image Séverine Tasker, Bristol Veterinary School, University of Bristol, UK.

Renomegaly, but also occasionally a reduction in kidney size, can occur. A pyogranulomatous pneumonia can occur [217,218], causing respiratory signs. Abdominal lymphadenomegaly and lymphadenopathy are common [17,30,132,219]. In one retrospective study of suspected cases of FIP [20], 41% of cats had a palpable abdominal mass on palpation, believed to be either mesenteric lymphadenomegaly or an intestinal mass. Mesenteric lymphadenomegaly and abdominal organomegaly were noted in 27% and 25% of 28 cats with FIP, respectively, in one report [17]. In another study of suspected FIP in cats without effusions or with ‘mixed’ signs of both effusive and non-effusive FIP (‘mixed’ was the terminology used by the authors to describe cases with signs of both) [31], 31% of cats had abdominal lymphadenopathy, but the size of the lymph nodes was not described. Jaundice can occur (Figure 5), more commonly in cats with effusions; although hyperbilirubinaemia is common, levels are often not high enough to result in clinical jaundice [18,21,45].

#### 6.2.2. Clinical Signs of FIP Associated with the Intestinal Tract

FIP can also manifest in the intestinal tract and/or regional lymph nodes (sometimes called a ‘focal form of intestinal FIP’ [220]), presenting typically as a palpable abdominal mass due to primary involvement of the MLNs and/or thickening of the intestinal tract. As mentioned above, in one study [20], 41% of cats with suspected FIP had a palpable abdominal mass, believed to be either mesenteric lymphadenomegaly or an intestinal mass. It can be particularly challenging to diagnose these cases as the lesions can be hard to initially differentiate from neoplasia [221], toxoplasmosis [222] or mycobacterial infection [223]. Diarrhoea is sometimes reported [2,20,45].

FIP involving the intestinal tract can manifest as a protein-losing enteropathy, leading to low total protein and globulin values, in contrast to the usual hyperglobulinaemia in FIP. Often, these cats present with MLN enlargement due to necrogranulomatous lymphadenitis [221,224], or solitary mural intestinal lesions of the colon or ileo-caecocolic junction with associated regional lymphadenopathy [220]. Cats with intestinal FIP usually have a history of weight loss, vomiting and diarrhoea or constipation.

#### 6.2.3. Clinical Signs of FIP Associated with the Skin

Dermatological signs are occasionally reported in FIP and can manifest as single or multiple non-pruritic or pruritic nodules or papules [225,226,227,228], due to pyogranulomatous-necrotising dermal phlebitis/vasculitis. Skin fragility syndrome was reported in a cat with FIP and concurrent hepatic lipidosis [229]. Idiopathic ulcerative dermatitis (IUD) has also been reported with FIP. In one report [230], IUD was diagnosed in a cat with uveitis, and the small ulcer on the dorsal neck was positive for the FCoV antigen when tested by IHC. However, in another report of a cat with IUD [231], the FCoV antigen IHC of the skin was negative, although FIP was confirmed by IHC on kidney tissue. Priapism has been reported as a result of granulomatous changes in tissues surrounding the penis [232].

#### 6.2.4. Clinical Signs of FIP Associated with the Nervous System

**Figure 6:**
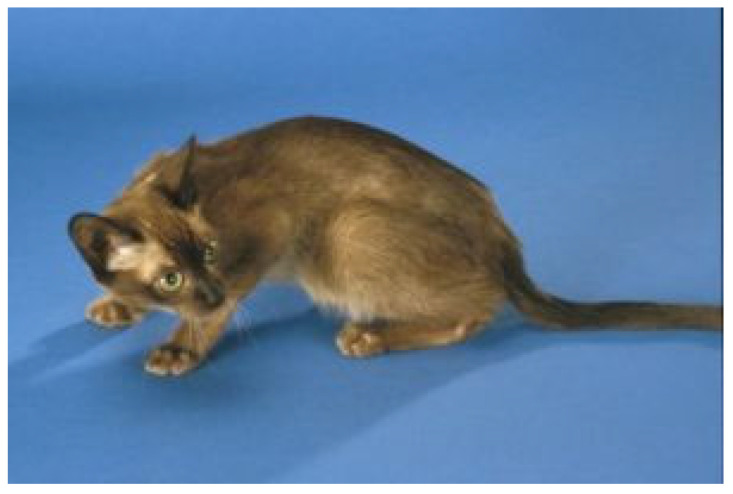
Ataxia can occur in cats with neurological FIP. Image Séverine Tasker, Bristol Veterinary School, University of Bristol, UK.

**Figure 7:**
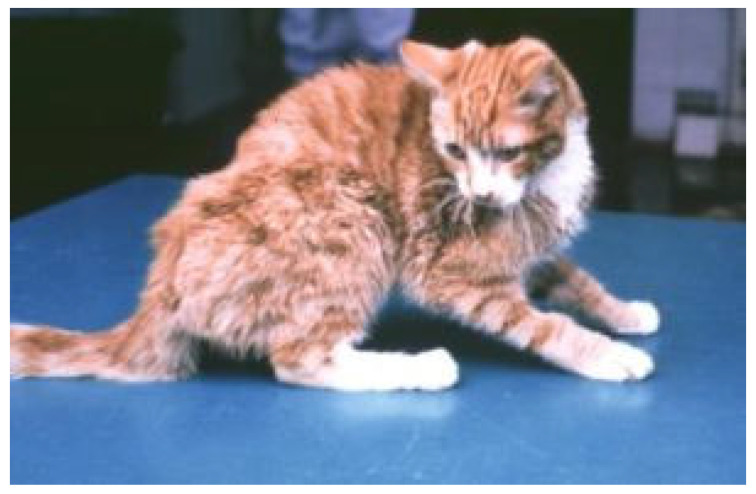
Ataxia (wide-based stance) and obtundation in a cat with neurological FIP. Image Allan May, University of Glasgow, UK through Diane Addie, www.catvirus.com.

Neurological FIP can result in clinical signs associated with focal, multifocal, or diffuse changes in the brain, spinal cord, and meninges. Up to 30% of cats with FIP show neurological signs [34,35,233,234,235,236,237,238]. Sometimes, cats with FIP present with only neurological disease [239]. Three clinical syndromes were identified in a retrospective study of neurological FIP [33]; of 24 cats, 3 had a T3-L3 myelopathy, 7 had central vestibular syndrome and 14 had multifocal CNS disease. Commonly reported signs include ataxia (with varying degrees of tetra- or paraparesis; Figure 6 and Figure 7), hyperaesthesia, nystagmus, seizures [240], behavioural and mental state changes, and cranial nerve deficits. Central vestibular clinical signs can include head tilt, vestibular ataxia, nystagmus, obtunded appearance, and postural reaction deficits; obtundation was reported in all five cats with FIP that presented with neurological signs in one case series [45]. Interestingly, a retrospective study [241] that reviewed cats presenting with vestibular disease did not identify any discrete clinical characteristics that would help differentiate cats with vestibular disease due to FIP from other causes. This was a surprise given that FIP primarily affects younger cats and is often associated with concurrent non-neurological signs. The absence of clinical characteristics specifically associated with FIP may have been because the study included a number of younger cats with other diagnoses (middle ear polyps, thiamine deficiency, intracranial empyaema and otitis media/interna), and cats with intracranial empyaema can have non-neurological systemic signs. Fever was less common in cats with neurological FIP compared to those without neurological signs [18]. A retrospective study [242] of cats referred for investigation of spinal disease found FIP to be the cause in 18 of 221 cats; concurrent systemic abnormalities and abnormal findings on clinical examination were significantly associated with a diagnosis of FIP, but these features were also associated with a diagnosis of spinal lymphoma (16 cats) and empyaema (3 cats).

#### 6.2.5. Clinical Signs of FIP Associated with the Eye

**Figure 8:**
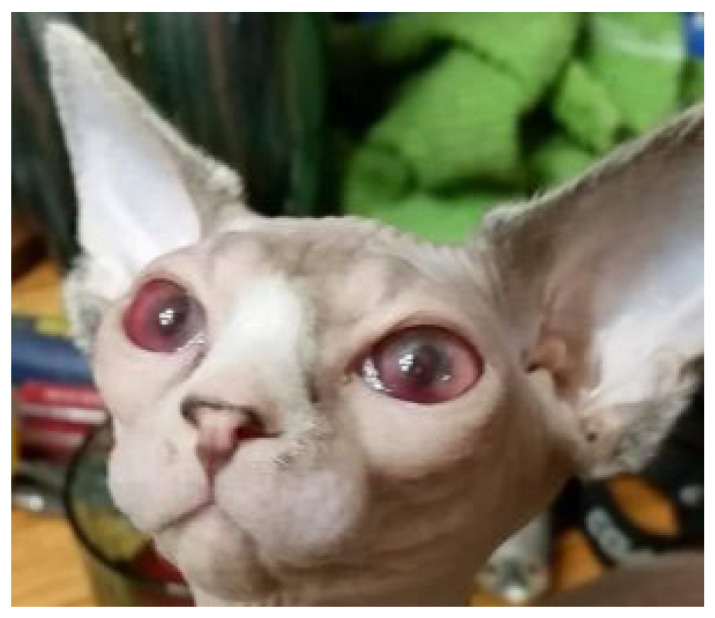
FIP-associated anterior uveitis can manifest variably such as with the presence of hyphaema. Image Maria Bonino and Erica Carter.

**Figure 9:**
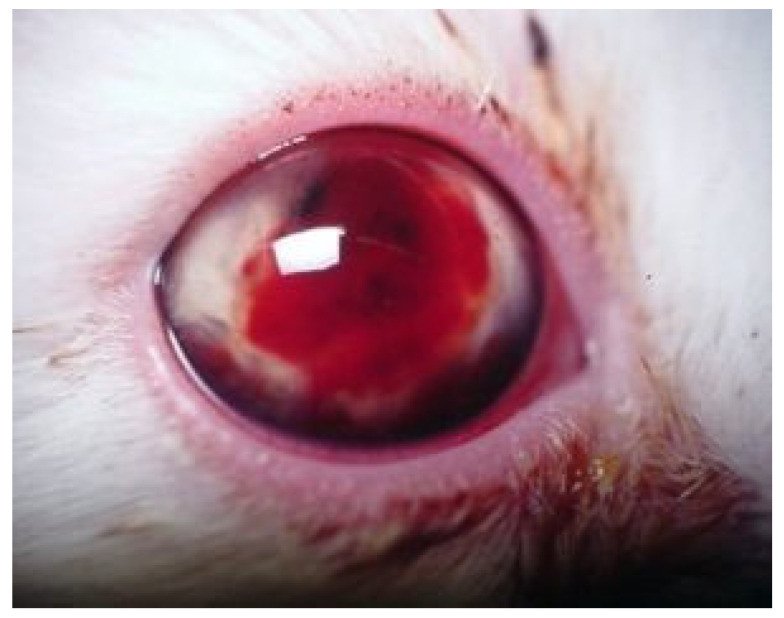
FIP-associated anterior uveitis can manifest variably such as with the presence of hyphaema. Image Albert Lloret, Universitat Autònoma Barcelona, Spain [46].

**Figure 10:**
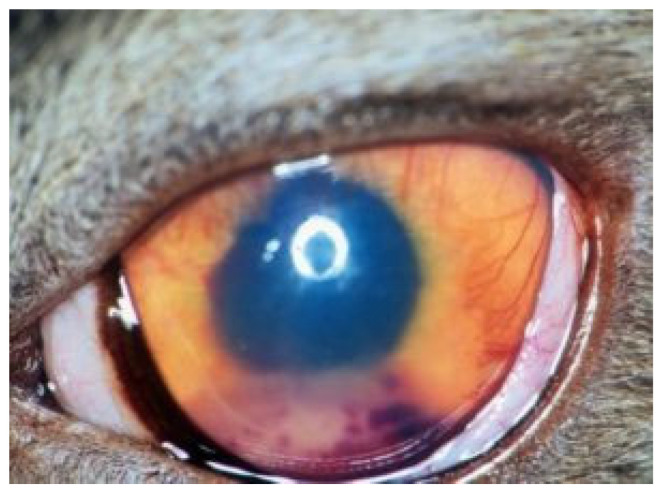
FIP-associated anterior uveitis can manifest variably such as with the presence of inflammatory keratic precipitates. Image Eric Déan, Vet-Oeil Ophthalmology Clinic, France [46].

FIP was the second-most-common cause of uveitis after idiopathic uveitis in studies of 120 cats with uveitis in the USA (16% had FIP) [243], and 92 cats with uveitis in the UK (again, 16% had FIP) [244]. A study describing the ocular lesions in 15 cats with FIP found effusions in 13 cats and no effusion in only 2 cats [32], although other authors have found a low prevalence of effusions in cats with FIP-associated uveitis [244]. Ocular manifestations of FIP comprise anterior and/or posterior uveitis [15,35,45,233,243] (Figure 8, Figure 9 and Figure 10), with anterior uveitis being more common [245]. The uveitis is unilateral or bilateral [244]. Important differential diagnoses include toxoplasmosis [246], lymphoma, feline immunodeficiency virus (FIV) and feline leukaemia virus (FeLV) infection [243,244]. Clinical signs include changes in iris colour, dyscoria or anisocoria secondary to iritis, sudden loss of vision and hyphaema (Figure 8 and Figure 9). Keratic precipitates can appear as ‘mutton fat’ deposits on the ventral corneal endothelium (Figure 10). The iris can show swelling and a nodular surface, and aqueous flare can be detected. On ophthalmoscopic examination, chorioretinitis, fluffy perivascular cuffing (representing retinal vasculitis), dull perivascular puffy areas of pyogranulomatous chorioretinitis, linear retinal detachment, vitreous flare and fluid blistering under the retina can be seen.

#### 6.2.6. Miscellaneous Clinical Signs of FIP

FIP-associated rhinitis [117] was described in a young cat that presented with some upper respiratory signs as well as other more typical signs of FIP; extensive respiratory panel testing on upper respiratory tract swabs in this cat revealed only a low positive test result for *Mycoplasma felis,* whilst the histopathological examination of lung (and liver and intestine) and nasal samples (including FCoV antigen IHC on the nasal samples) confirmed a diagnosis of FIP. Another report described three cats with FIP that had presented with mild upper respiratory signs before showing other more typical signs of FIP (fever, icterus, lethargy, anorexia, effusions) within the following 10 days [55].

Myocarditis associated with FIP has also been described in a cat without effusion [247]; this particular case had presented with fever, weight loss and diarrhoea before developing dyspnoea and then neurological and ocular signs of FIP. The histopathology of various organs, including cardiac tissue, was consistent with FIP, and the FCoV antigen IHC of the heart was also positive.

**Summary of Section 6: Clinical Signs** *FCoV Infection* **Cats with FCoV infection are usually subclinical**, although **occasionally diarrhoea and/or vomiting and poor growth (in kittens)** can occur. *FIP* Cats that go on to develop FIP after FCoV infection present with varied clinical signs depending on the distribution of vasculitis (which can lead to effusions) and/or (pyo)granulomatous lesions (which can lead to mass lesions) in the body. Although **effusive and non-effusive forms of FIP** are often described, there is **much overlap** between these forms. Clinical signs of FIP can change over time, and therefore **repeated physical examinations** are important to detect newly apparent clinical signs; for example, an effusion can develop, or ocular changes can become visible on ophthalmoscopic examination. ABCD FIP Diagnostic Approach Tools [211] are available to help the vet assess clinical signs for FIP. Non-specific clinical signs include **lethargy, anorexia, and weight loss (or failure to gain weight/stunted growth in kittens)**. A **fever** that is refractory to treatment is common. **Effusions are common**, especially in the **abdomen**, but **pleural effusions and pericardial** effusions are also seen, sometimes concurrently. When effusions are present, the disease progression is often quite fast, within a few days or weeks. When effusions are not present, FIP is often more difficult to diagnose and it also tends to be more chronic, progressing over a few weeks to months. Additional signs of non-effusive FIP depend on the organs affected but can include the central nervous system, eyes and/or abdominal organs (such as the liver, abdominal lymph nodes [especially mesenteric lymph nodes], kidney [including renomegaly], pancreas, spleen and/or gastrointestinal tract). These signs can also be present in cats with effusions. **Abdominal lymphadenomegaly or intestinal masses** (sometimes palpable), can occur. **Jaundice** can occur, more commonly in cats with effusions, but the degree of hyperbilirubinaemia is often not high enough to result in clinical jaundice. Occasionally, cats with FIP show **skin signs**. Neurological signs seen with FIP include **ataxia** (with varying degrees of tetra- or paraparesis), **hyperaesthesia, nystagmus, seizures**, behavioural and mental state changes, and cranial nerve deficits. Central vestibular clinical signs can include head tilt, vestibular ataxia, nystagmus, obtunded appearance, and postural reaction deficits. Fever was shown to be less common in cats with neurological FIP compared to those without neurological signs. FIP can also cause unilateral or bilateral **uveitis**. Clinical signs include changes in iris colour, dyscoria or anisocoria secondary to iritis, sudden loss of vision and hyphaema. **Keratic precipitates** can appear as ‘mutton fat’ deposits on the ventral corneal endothelium, and aqueous flare can occur. On ophthalmoscopic examination, chorioretinitis, fluffy perivascular cuffing (representing retinal vasculitis), dull perivascular puffy areas of pyogranulomatous chorioretinitis, linear retinal detachment, vitreous flare and fluid blistering under the retina can all be seen. Other less-common signs associated with FIP have included rhinitis and clinical signs associated with myocarditis.

## 7. Diagnosis of FIP

This section will focus on the diagnosis of FIP in sick cats showing clinical signs that could be suggestive of FIP. A cat cannot develop FIP unless it has been previously infected with FCoV and so the demonstration of FCoV (as RNA or antigen) in affected tissues and effusions, with other findings (e.g., biochemistry, cytology) consistent with FIP, is helpful during diagnostic investigations of FIP.

**Figure 11:**
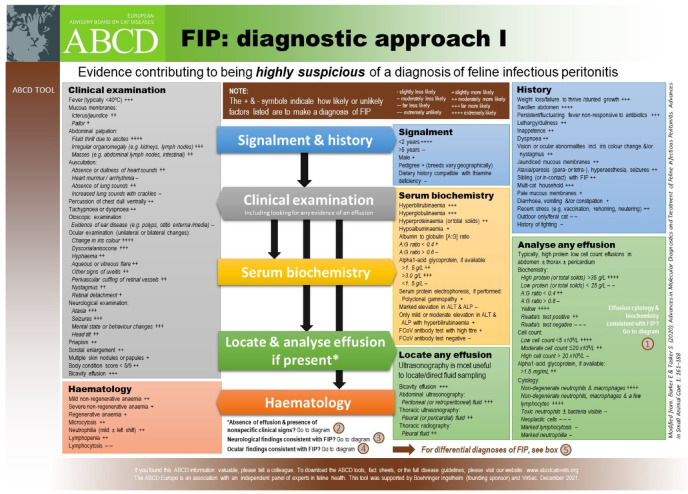
European Advisory Board on Cat Diseases (ABCD) Feline Infectious Peritonitis (FIP) Diagnostic Approach Tools: diagnostic approach I, showing evidence that can contribute to being highly suspicious of a diagnosis of FIP. This tool is available online [211], with revisions made to the online version as required. Many features of the cat’s signalment, history and clinical examination can contribute to a suspicion of FIP. Effusion analysis is always extremely helpful, so looking for evidence of an effusion and then sampling should be prioritised whenever possible. Certain haematological features can also contribute to the suspicion of FIP as a diagnosis.

**Figure 12:**
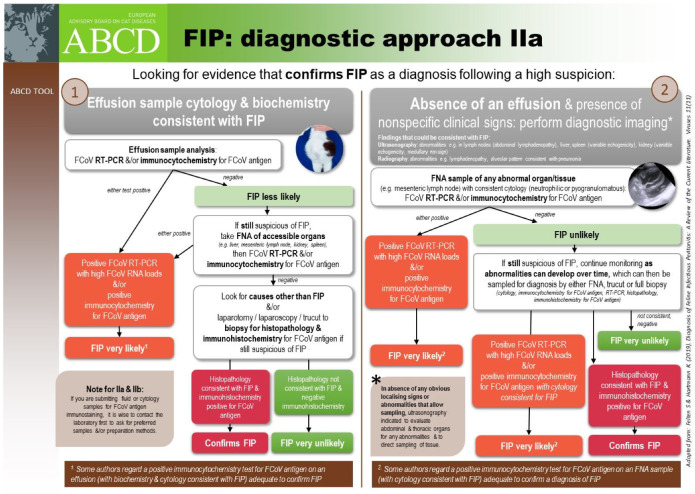
European Advisory Board on Cat Diseases (ABCD) Feline Infectious Peritonitis (FIP) Diagnostic Approach Tools: diagnostic approach IIa, showing diagnostic testing evidence that can confirm FIP as a diagnosis following being highly suspicious of FIP in cats with an effusion (1) and cats that neither have effusions nor specific clinical signs (2). This tool is available online [211], with revisions made to the online version as required.

**Figure 13:**
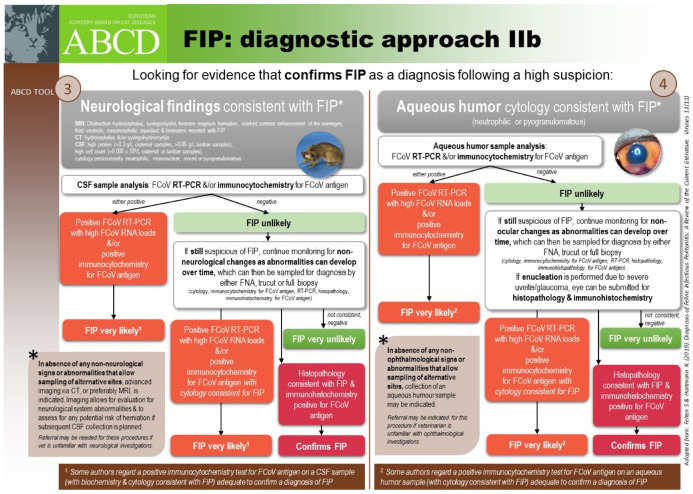
European Advisory Board on Cat Diseases (ABCD) Feline Infectious Peritonitis (FIP) Diagnostic Approach Tools: diagnostic approach IIb, showing diagnostic testing evidence that can confirm FIP as a diagnosis following being highly suspicious of FIP in cats with neurological signs (3) and cats with aqueous humour cytology consistent with FIP (4). This tool is available online [211], with revisions made to the online version as required. In this figure, the confirmation of a diagnosis of FIP requires the collection of cerebrospinal fluid (CSF) or aqueous humour. However, it is generally easier to sample effusions, if present, or accessible abnormal organs or tissues (e.g., mesenteric lymph node, identified by imaging) by fine-needle aspiration, if present, as indicated in Figure 12.

**Figure 14:**
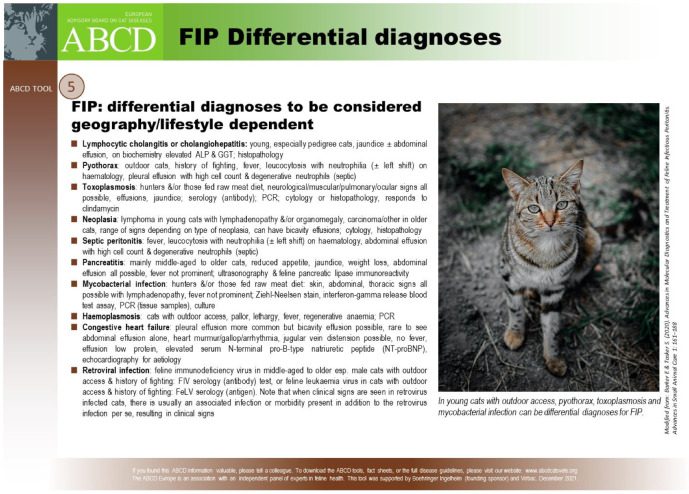
European Advisory Board on Cat Diseases (ABCD) Feline Infectious Peritonitis (FIP) Diagnostic Approach Tools: differential diagnoses. This tool is available online [211], with revisions made to the online version as required.

The ABCD FIP Diagnostic Approach Tools found online [211] and in Figure 11, Figure 12, Figure 13 and Figure 14 show an overview of criteria that can be used to confirm a diagnosis of FIP or make a diagnosis of FIP very likely. Now that effective antivirals for the treatment of FIP exist, the trial treatment of cases without a confirmed diagnosis of FIP, but in which the diagnosis is very likely (Figure 11, Figure 12 and Figure 13), can be warranted, as the response to effective antivirals is usually rapid. This is discussed in Section 10 on Treatment of FIP. Further information on the diagnostic tests mentioned in Figure 11, Figure 12, Figure 13 and Figure 14 is in this section.

### 7.1. Signalment and History for FIP

When considering FIP as a differential diagnosis, one must remember that FIP is more common in young cats (especially under two years old [11,13,15,18,19,20]) and that male cats [13,14,15,16,18,76] are at a slightly higher risk of disease, according to some studies. However, cats of any age or sex can be affected. In one study, the median age of a group of cats with FIP without effusions was 39 months [212]. Additionally, most cats that develop FIP come from multi-cat households or have a history of having been housed in multi-cat households. Although certain breeds have been shown to be predisposed to FIP in certain countries [11,14], it is believed that this is due to genetic risk factors being present in those breeds in those countries rather than the existence of worldwide generalised breed predispositions [18], although a predilection for pedigrees has been reported [11,12,13,14,15,16]. A recent history of stress (e.g., adoption, being in a shelter, neutering, upper respiratory tract disease, vaccination, travel, new household member) is commonly apparent [18,20,248] and may contribute to the development of FIP in a FCoV-infected cat.

**Summary of Section 7: Diagnosis of FIP; Section 7.1: Signalment and History for FIP** FIP is more common in **young cats** (especially under two years old) and **some pedigree breeds**, and **male cats** are at slightly higher risk of disease. Additionally, most cats that develop FIP come from **multi-cat households** or have a history of having been housed in multi-cat households. A **recent history of stress** (e.g., adoption, being in a shelter, neutering, upper respiratory tract disease, vaccination) is common.


### 7.2. Approach to the Diagnosis of FIP

**Figure 15:**
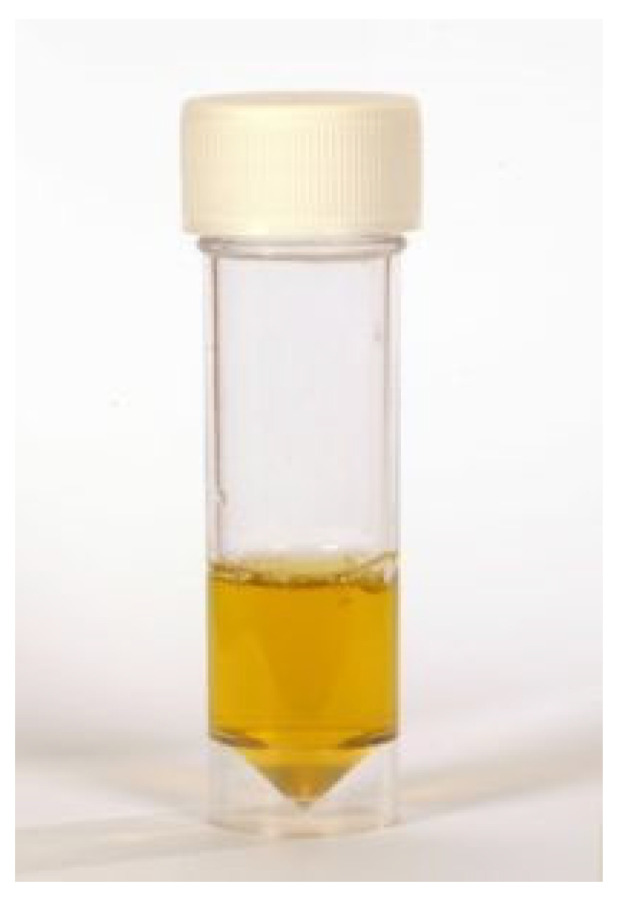
Abdominal effusion sample collected from a cat with FIP showing typical clear straw-yellow-coloured fluid. Image Séverine Tasker, Bristol Veterinary School, University of Bristol, UK.

In cats with FIP that have an effusion, sampling the effusion is the single most useful diagnostic step (Figure 11 and Figure 12); this is because tests on effusions often have a higher diagnostic value in comparison to tests on blood [249], and effusion samples are often relatively easy to obtain. If the effusion is not large in volume, imaging can be used [250] to confirm, identify and localise smaller volumes. Ultrasonography is generally regarded as being more sensitive than radiography for this, but it depends on where pockets of fluid reside (see Section 7.4.1 on Routine Imaging: Ultrasonographic and Radiographic Findings). Repeated ultrasonography to identify any small-volume effusion is recommended and, similarly, ultrasonography can be used to guide the sampling of small pockets of fluid [251]. Once an effusion is sampled, the first thing to do is to take note of its appearance: if it is frank blood, or if it can be discerned as urine, FIP is very unlikely. Additionally, purulent exudates are usually not caused by FIP [252], although occasionally bacterial translocation in cats with effusions can complicate diagnosis (Séverine Tasker, personal communication). The presence of chyle will usually indicate other diseases, such as heart failure, lymphoma or a ruptured thoracic duct, but cats with FIP with pure chylous effusion have been reported [253]. FIP effusions are usually clear, viscous/sticky and straw-yellow in colour (Figure 15).

Diagnosing FIP if no effusion is present, however, can be very challenging due to the high number of possible clinical signs and the non-specificity of most of them (e.g., anorexia, lethargy, weight loss, fever) and the lack of accessible fluid to sample. Peritoneal lavage can be performed by instilling 20 mL/kg of 0.9% saline into the peritoneal cavity, massaging the abdomen, and withdrawing the fluid by paracentesis [254,255], although the value of analysis of lavage fluid in the diagnosis of FIP of cases without effusions is not clear [255]. The definitive diagnosis of FIP in cases that do not have effusions, by collection of tissue biopsies ante-mortem, for histopathology and IHC, can be very difficult due to, for example, problems accessing affected tissues, contra-indications for general anaesthesia or invasive biopsy collection in a sick cat, and/or costs involved in collection. Cases with neurological or ocular signs can be approached via the sampling of cerebrospinal fluid (CSF) or aqueous humour, although these techniques are not commonly performed in non-referral veterinary clinics. Currently, there is no non-invasive, confirmatory test available for cats with FIP that do not have effusions, although valuable information can be gained through the analysis of FNA samples for cytology and FCoV antigen or RNA detection following collection from affected organs, if accessible, as described below. Tissue FNA samples are usually easier to obtain than tissue biopsies.

The information in this review will consider the merits and drawbacks (and sometimes sensitivity and specificity) of tests available for the diagnosis of FIP, and FCoV infection if relevant. Although each individual test will be described, it should be remembered that when a cat with suspected FIP is investigated, a veterinarian will be interpreting several test results at the same time, as well as taking into account the signalment and history of the cat. Such interpretation is important in helping to determine how likely FIP is as a diagnosis, in the absence of a definitive diagnosis. The advantage of integrating multiple test results during interpretation has been shown [256]; additionally, machine learning can be applied to the diagnosis of FIP [257].

Now that effective antiviral treatments, such as GS-441524 [20,24], are available for FIP (see Section 10 on Treatment of FIP), a rapid and sustained positive response to antiviral treatment is also a means of supporting a diagnosis of FIP.

**Summary of Section 7: Diagnosis of FIP;** Section 7.2**: Approach to the Diagnosis of FIP:** If an **effusion is present, sampling it is the single most useful diagnostic step** because tests on effusions have a higher diagnostic value compared to those on blood samples. Samples of effusion can be easy to obtain; **imaging (especially ultrasonography) is used to confirm, identify, localise, and sample smaller volumes**. FIP effusions are usually clear, viscous/sticky and straw-yellow in colour. **Diagnosing FIP if there is no effusion present is more challenging** due to the **large number of possible clinical signs and their non-specific nature** (e.g., anorexia, lethargy, weight loss, fever) and because **biopsy collection ante-mortem can be very difficult** due to, for example, problems accessing affected tissues, contra-indications for general anaesthesia or invasive biopsy collection in a sick cat, and/or costs involved in tissue collection. Cases with neurological or ocular signs can be approached via the sampling of cerebrospinal fluid or aqueous humour, but these techniques are not performed commonly outside of referral clinics. There is no non-invasive, confirmatory test available for cats with FIP that do not have effusions, although in some cases valuable supportive information can be gained through the analysis of fine-needle aspirate (FNA) samples collected from affected organs, if accessible. **Tissue FNAs are usually easier to collect than tissue biopsies.** The integration of multiple test results is most useful to help direct the clinician to a diagnosis of FIP being very likely, in the absence of confirmatory testing.

### 7.3. Laboratory Changes in FIP

#### 7.3.1. Routine Haematology

Routine haematological changes are not specific for FIP, but common abnormalities are lymphopenia (seen commonly and maybe more in cats with effusions than in cats without), neutrophilia, a left shift, and a mild-to-moderate normocytic, normochromic anaemia [15,18,20,26,27,43,45,212,258,259]. No difference in the likelihood of anaemia was found between cats with and without effusions in one study [43]. An association between FIP and microcytosis (with or without anaemia) has been reported [18]. Immune-mediated haemolytic anaemia occasionally occurs [15,18]. A decreasing red-blood-cell count is a poor prognostic sign [27,212] and, indeed, a reversal of the anaemia occurs in successfully treated cats [43,212].

#### 7.3.2. Serum Biochemistry

Serum biochemistry changes are also non-specific in cats with FIP, but certain abnormalities can be helpful in making one consider FIP as more likely as a diagnosis.

Hyperglobulinaemia is often reported in FIP and can be accompanied by hypoalbuminaemia or low-to-normal serum albumin [18,45,258]. The presence of hypoalbuminaemia alongside hyperglobulinaemia means that hyperproteinaemia is not always present [18]. The albumin to globulin (A:G) ratio can be low, and the value of this ratio can be used to help evaluate how likely FIP is; the A:G ratio has a higher diagnostic value than either total serum protein or globulin concentration [249]. Various A:G ratio cut offs have been suggested, e.g., an A:G ratio of less than 0.4 makes FIP very likely, whilst an A:G ratio of greater than 0.8 makes FIP very unlikely [15,26,27]. One study [260] on a population of cats with a prevalence of FIP of 4%, reported that a serum A:G ratio of greater than 0.6 was useful in ruling out FIP, but that lower ratios were not helpful in ruling in FIP. Additionally, the frequency and magnitude of hypoalbuminaemia, hyperglobulinaemia and low A:G ratios reported in cats with FIP have decreased in more recent years [18,261], which could be due to veterinarians diagnosing FIP earlier, meaning that cases have not progressed to show these changes. Polyclonal and monoclonal elevated γ-globulins have been reported in cats with FIP [262], although polyclonal elevations are far more common. In one study, a low A:G ratio was found to be a negative prognostic indicator in cats with FIP given immunostimulant treatment [212].

Increased bilirubin levels, in the absence of both haemolysis and moderate elevations of liver enzyme activity, should raise the suspicion of FIP. Hyperbilirubinaemia occurs in 22–84% of cats with FIP [15,18,26,27,45], and is especially seen in FIP cases with effusions [18]. Increased bilirubin values are not always correlated with elevated liver enzymes [18], as hyperbilirubinaemia in cats with FIP is not necessarily a reflection of parenchymal liver disease. Alanine aminotransferase (ALT), aspartate transferase (AST) and alkaline phosphatase (ALP) were normal in 86%, 66% and 95%, respectively, of cats with FIP [18]. The hyperbilirubinaemia may be due to excessive erythrocyte fragility, leading to haemolysis, with the reduced clearing of haemoglobin breakdown products [9], or altered bilirubin metabolism due to high TNF-α levels, leading to reduced bilirubin transport into and out of liver cells. It has been found that the level of bilirubin can rise as FIP progresses, and that rising bilirubin levels (and falling red-blood-cell counts) are a poor prognostic sign [27]. Indeed, a study [19] evaluating the response to nucleoside analogue antiviral treatment, of cats with suspected effusive FIP found that the total bilirubin levels in those that survived were significantly lower than those that did not, suggesting that circulating total bilirubin levels might be a prognostic risk factor for response to treatment in effusive FIP. A similar finding by the same group was found [31] in cats with ‘mixed’ effusive and non-effusive FIP.

As described earlier, the kidneys can be affected in FIP via pyogranulomatous lesions or glomerulonephritis [263]; these changes can result in azotaemia, although this is more common in cases without effusions [18].

Hypoglycaemia was reported in 5 of 32 (15%) cats with FIP; this may reflect disease severity, the presence of severe inflammatory response syndrome, or sepsis [45].

Acute phase proteins (APPs) are produced by the liver in acute infections and many inflammatory and non-inflammatory diseases in response to cytokines released from macrophages and monocytes. The major APPs in cats are α1-acid glycoprotein (AGP) and serum amyloid A (SAA).

AGP has an immunomodulatory function, and assays are available for its measurement in laboratories in some countries. The reference range for AGP serum concentrations is less than 0.48 mg/mL (less than 480 µg/mL) [264], and a moderately elevated serum AGP concentration [31] and concentrations of greater than 1.5 mg/mL [43,256], are frequently reported in cats with FIP. The magnitude of the increase in serum AGP might be helpful in the diagnosis of FIP [264,265,266,267]. One report [267] found that markedly elevated serum AGP concentrations of greater than 3 mg/mL could support a diagnosis of FIP in cats with a low pre-test probability of disease (i.e., with a history and clinical findings not typical of FIP), whereas less marked elevations were supportive of a diagnosis of FIP in cats with a higher pre-test probability of disease (i.e., with a history and clinical findings more typical of FIP). However another, albeit very small, study of cats with FIP actually found that even moderately elevated AGP concentrations of greater than 1.5 mg/mL were still able to discriminate between cats with and without FIP [265]; interestingly, this study comprised unusual cases of FIP with atypical presentations, although a diagnosis of FIP was confirmed in all cases. However, it must be emphasised that AGP is not specific for FIP and can be increased in other diseases. It has been suggested that an AGP concentration of less than or equal to 1.5 mg/mL could be useful to rule out FIP [256]. AGP concentrations have been found to increase moderately and transiently in all the cats in a household before the appearance of cases of FIP in an environment with endemic FCoV infection [268]. It has also been found that AGP is often hyposialylated in cats with FIP, but not usually in clinically healthy FCoV antibody-positive cats or cats with other diseases [269,270]. However, testing for the sialylation of AGP is not available routinely. A feline immunometric enzyme-linked immunosorbent assay (ELISA) has become commercially available to measure feline AGP [271]; a reference interval of less than 0.33 mg/mL (less than 328 µg/mL) was established, although a serum sample dilution of 32,000 was used in the study, compared to the 10,000 recommended by the manufacturer, although different laboratories might use different cut-off values. AGP measurement may be useful to differentiate likely cure of FIP from only remission after treatment [43], as described in Section 10 on Treatment of FIP.

SAA is also markedly increased in cats with FIP [20,24,157], especially in cats with FIP and effusions compared to those without effusions [19,31]. Although AGP was more useful than SAA in one diagnostic study [266], further work is required to evaluate its diagnostic and prognostic value. Additionally, SAA tests are more widely available than AGP tests in some parts of the world.

#### 7.3.3. Cytology and Biochemistry on Effusions

As described under imaging, ultrasonography or radiography can be used to identify or confirm the presence of effusions and to assist in sample collection [36,250], which can be important as the analysis of a sample of effusion is very helpful in the diagnosis of FIP.

FIP effusions are highly proteinaceous [45], with a total protein concentration that is usually greater than 35 g/L, consistent with that of an exudate. An early study [272] describing the characteristics of effusions of 12 cats with FIP reported total protein concentrations of 32–99 g/L (median 59 g/L). In contrast, the cell counts of effusions due to FIP are often relatively low, usually less than 5 × 10^9^/L cells (which would be more consistent with a modified transudate); however, sometimes, cell counts are higher, for example, up to around 20 × 10^9^/L cells. Slides for cytological examination can be prepared from effusions by making direct smears with the fluid onto microscope slides if the effusion is turbid (cloudy); if the effusion is clear with minimal turbidity, the centrifugation of a sample of effusion and preparing smears from the pellet (following resuspension in around 0.5 mL of effusion) can improve smears’ diagnostic yield [36]. Effusion cytology is typically pyogranulomatous in nature with macrophages, non-degenerate neutrophils, and few lymphocytes. Thick eosinophilic (pink-red) proteinaceous backgrounds are often also described on cytology [20]. If cytology reveals a septic neutrophilia (typically with degenerate neutrophils containing bacteria), neoplastic cells, or a marked lymphocyte population, FIP is highly unlikely [273].

The effusions of cats with FIP typically have low A:G ratios; an A:G ratio of less than 0.4 has a high positive predictive value, whereas a value of greater than 0.8 has a high negative predictive value [18,272]. One study found that elevated effusion AGP concentrations (of greater than 1.55 mg/mL) were more useful (sensitivity and specificity of 93%) in differentiating the effusions of cats with FIP from those of cats without FIP when compared with AGP levels in the serum or other acute phase proteins [266]; however, the diagnosis of FIP in the cats in this study was not always confirmed.

#### 7.3.4. Rivalta’s Point-of-Care Test on Effusions

Rivalta’s test is a crude point-of-care assay that was originally developed to differentiate a transudate from an exudate in humans. It is important to note that a positive result is not specific for FIP, and positive results have been reported in cats without FIP, for example, in those with septic peritonitis and lymphoma [274]. The positive predictive value was 58% in a study of cats who presented with effusion, in which the prevalence of FIP was 35% [274]. If positive, effusion cytology can be helpful to discriminate between these causes [273]. The Rivalta’s test had a high negative predictive value of 93% for the exclusion of FIP [274], making it useful to rule out FIP quickly and cheaply at point-of-care. A positive result needs confirmation with other tests.

**Figure 16:**
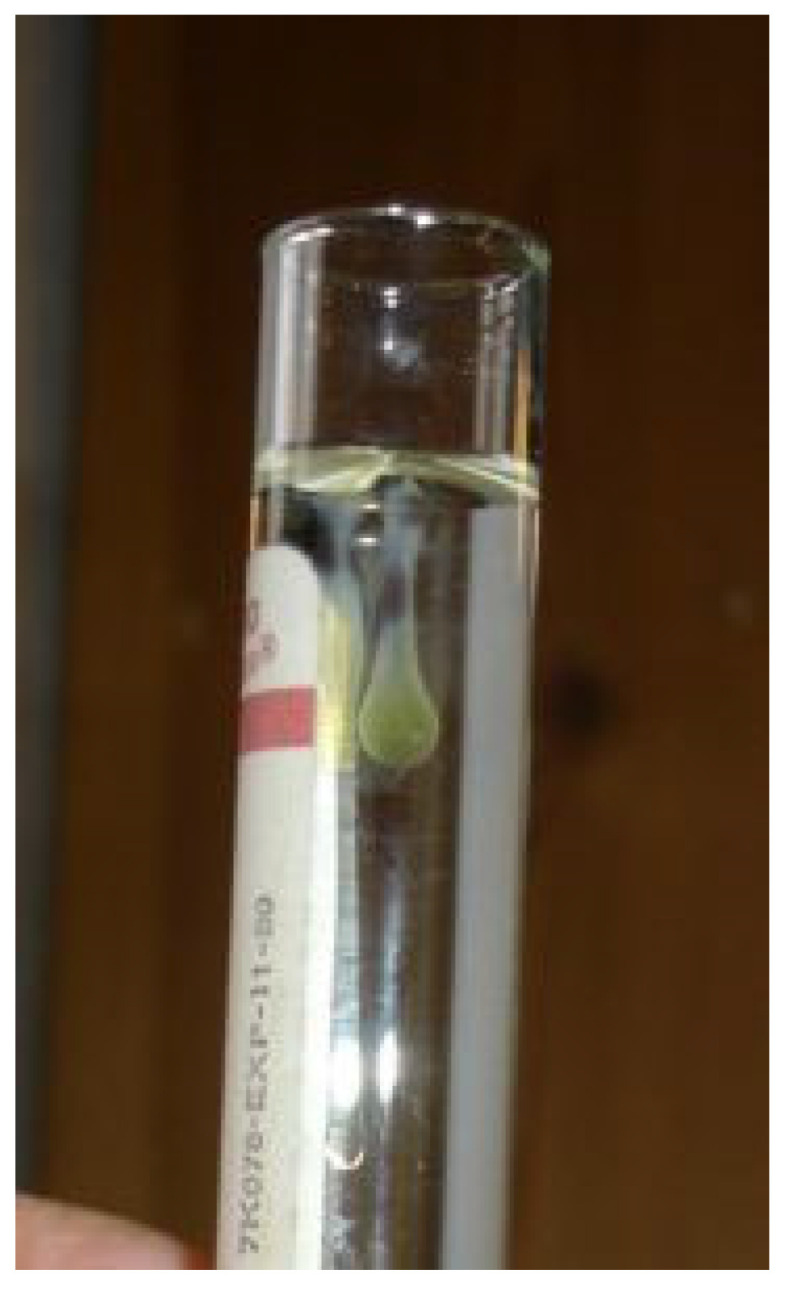
This image shows a positive Rivalta’s test; a drop of abdominal effusion has been placed onto the surface of a mixture of 8 mL of distilled water and one drop of 98% acetic acid (or white vinegar) in a test tube, and it has retained its shape with a connection to the solution surface. This is not very specific for FIP but can be performed in-house; a positive test increases the likelihood of FIP, while a negative test makes FIP very unlikely. Image Diane Addie, www.catvirus.com.

To perform the Rivalta’s test, a commercially available point-of-care test can be used, or the test can be made up in-house. For the latter, 8 mL of distilled water at room temperature and one drop of 98% acetic acid (or alternatively white vinegar) [275] are mixed in a test tube. One drop of effusion is then carefully placed or layered onto the surface of the solution in the test tube. A positive Rivalta’s test is indicated by the drop staying attached to the surface of the solution, retaining its shape with a connection to the surface, or floating slowly to the bottom of the tube as a drop or like a jellyfish (Figure 16). A negative test is indicated by the drop disappearing and the solution remaining clear. However, the interpretation of results can be problematic due to subjectivity and difficulties in deciding whether a result is positive or negative [275]. Using cold water can result in a false-negative reaction (Diane Addie, personal communication).

#### 7.3.5. FNA Cytology

There are few data on the sensitivity and specificity of FNA cytology in the diagnosis of FIP. One study compared the usefulness of hepatic and renal FNA cytology and tru-cut biopsy (TCB) histopathology in samples collected from cats with FIP confirmed by histopathology and FCoV immunostaining [276]. In this study, the cytological and histopathological findings of the FNAs and TCBs were classified according to whether they were consistent with FIP for calculation of sensitivity. Typical FNA cytological features of FIP were: highly cellular samples containing the normal cell population of the sampled organs (e.g., hepatocytes, renal tubular epithelial cells), but also neutrophils, macrophages, plasma cells, and lymphocytes, supporting a diagnosis of pyogranulomatous inflammation. The sensitivity of FNAs and TCBs from hepatic (82% and 64%, respectively) and renal (42% and 39%, respectively) tissues was poor, although combining the analysis of TCB and FNA results for each of the tissues increased sensitivity (to 86% for hepatic and 48% for renal). However, specific lesions within the liver and kidneys were not targeted for sampling in this study, and targeted sampling under ultrasound guidance might have improved sensitivity (see Section 7.4 on Diagnostic Imaging in FIP).

One study of cats with FIP described lymph node cytology sampling in 10 cats, with all 10 showing pyogranulomatous inflammation [45].

#### 7.3.6. CSF Analysis

Information on the CSF sampling technique is described in detail elsewhere [277], but referral or consultation with a neurologist for those unfamiliar with the technique is recommended. Although CSF samples are commonly collected from cats with neurological signs, care must be taken with cisternal CSF sampling as the risk of brain herniation is high [238,239,278,279] due to increased intracranial pressure that can arise, for example, in association with hydrocephalus due to FIP. Thus, ideally, advanced imaging, such as magnetic resonance imaging (MRI) and computed tomography (CT), should be performed before CSF sampling to assess the potential risk of herniation.

CSF samples from cats with FIP can show elevated protein concentrations (of greater than 0.30 g/L [greater than 30 mg/dL] in cisternal samples, and greater than 0.46 g/L [greater than 46 mg/dL] in lumbar samples with reference ranges of less than or equal to 0.30 g/L and less than or equal to 0.46 g/L for cisternal and lumbar CSF samples, respectively); occasionally, marked elevations of protein occur (greater than 20 g/L [200 mg/dL]). Additionally, CSF samples of cats with FIP often have an increased cell count (greater than 0.008 × 10^9^/L [greater than 8 cells/µL] in either lumbar and/or cisternal samples; reference range less than or equal to 0.008 × 10^9^/L [less than or equal to 8 cells/µL]); occasionally this pleocytosis is extremely marked in cats with FIP (cell counts of greater than 1 × 10^9^/L [greater than 1000 cells/µL]). Cytological examination of the CSF can show the pleocytosis to be predominantly neutrophilic, mononuclear, mixed, or pyogranulomatous [33,280,281]. Some cats with neurological FIP have unremarkable CSF analysis results [35,282].

#### 7.3.7. Aqueous Humour Analysis

Information on the aqueous humour sampling technique is described in detail in the literature [283], but referral or consultation with an ophthalmologist for those unfamiliar with the collection technique is recommended. Aqueous humour samples from cats with FIP show cytological features similar to what is found in CSF samples, i.e., mixed inflammation with neutrophils with or without macrophages.

**Summary of Section 7: Diagnosis of FIP; Section 7.3: Laboratory Changes in FIP:** Routine haematology and serum biochemistry Routine haematological changes are not specific for FIP, but common abnormalities include **lymphopenia, neutrophilia**, sometimes with a left shift, and a mild-to-moderate normocytic, normochromic anaemia. Serum biochemistry changes are more helpful and include **hyperglobulinaemia**, accompanied by hypoalbuminaemia or low-to-normal serum albumin and a **low albumin to globulin (A:G) ratio of less than 0.4** (an A:G ratio of greater than 0.8 makes FIP very unlikely). **Increased bilirubin levels in the absence of haemolysis or elevations of liver enzyme activity raise the suspicion of FIP**. **Acute phase proteins** (APPs) are produced in the liver in many inflammatory and non-inflammatory diseases; the major APP in cats is **α1-acid glycoprotein (AGP),** and **moderately elevated serum AGP concentrations** of greater than 1.5 mg/mL often **occur with FIP**. Another important APP in cats is **serum amyloid A**, more readily available in some countries, which is also markedly increased in cats with FIP. Cytology and biochemistry of effusions FIP **effusions** are **highly proteinaceous**, with a total protein concentration greater than 35 g/L, consistent with an exudate, but with **relatively low cell counts** of less than 5 × 10^9^/L cells, more consistent with a modified transudate; however, sometimes, cell counts rise to 20 × 10^9^/L. Cytology is **pyogranulomatous,** with macrophages, non-degenerate neutrophils and few lymphocytes. **Thick eosinophilic** (pink-red) **proteinaceous backgrounds** on cytology slides are often described. If cytology reveals a septic neutrophilia (typically with degenerate neutrophils containing bacteria), neoplastic cells or a marked lymphocyte population, other diseases are more likely. The **Rivalta’s test** is a crude **point-of-care assay** to identify proteinaceous inflammatory exudates, which occur with FIP, but also septic peritonitis and lymphoma. If positive, effusion cytology can be helpful to discriminate between these causes. **A negative Rivalta’s test, however, is more helpful to rule out FIP.** To perform the Rivalta’s test, 8 mL of distilled water at room temperature and one drop of 98% acetic acid (or white vinegar) are mixed in a test tube, and then one drop of effusion is carefully placed or layered onto the surface of the solution. A positive result is indicated by the drop staying attached to the surface of the solution, retaining its shape with a connection to the surface, or floating slowly to the bottom of the tube as a drop or like a jellyfish. A negative test is indicated by the drop disappearing and the solution remaining clear. However, the interpretation of results can be subjective, and it can be hard to decide whether a result is positive or negative. Cytology of fine-needle aspirates (FNAs), cerebrospinal fluid (CSF) or aqueous humour samples, and biochemistry, if applicable Typical **FNA** features of FIP are highly cellular samples containing the normal cell population of the sampled tissues with the additional presence of neutrophils, macrophages, plasma cells, and lymphocytes, consistent with **pyogranulomatous inflammation**. An examination of the **CSF** can show a **pleocytosis**, predominantly neutrophilic, mononuclear, mixed or pyogranulomatous in nature, with **elevated protein** concentrations. The cytology of **aqueous humour** can show **pyogranulomatous or mixed inflammation** with neutrophils with or without macrophages.

### 7.4. Diagnostic Imaging in FIP

#### 7.4.1. Routine Imaging: Ultrasonographic and Radiographic Findings

**Figure 17:**
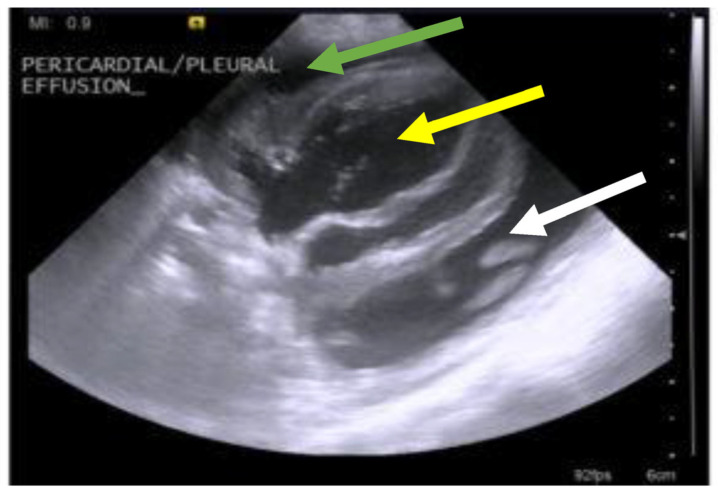
Ultrasonogram of a young cat with FIP showing pericardial and pleural effusion; ultrasonography can be used to guide sampling of the effusion. Green arrow = pericardial fluid. Yellow arrow = cardiac ventricle. White arrow = pleural effusion. Image Séverine Tasker, Bristol Veterinary School, University of Bristol, UK.

**Figure 18:**
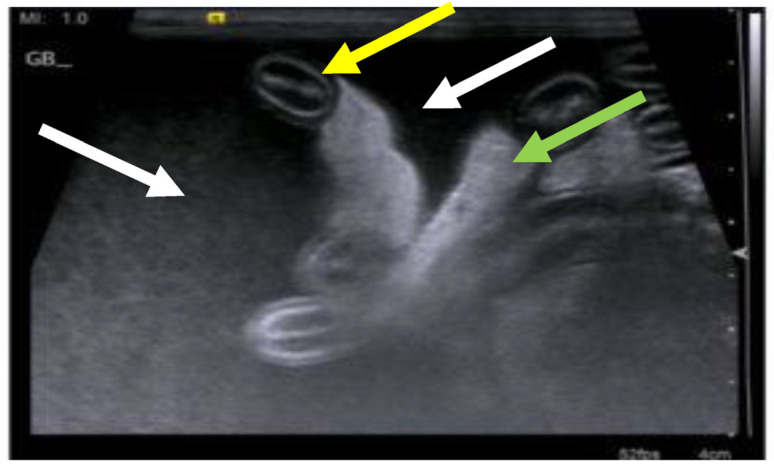
Ultrasonogram of a young cat with FIP showing abdominal effusion; ultrasonography can be used to guide sampling of the effusion. Yellow arrow = small intestinal loop in transverse section. White arrows = peritoneal effusion—note the effusion is echogenic, suggesting cellularity. Green arrow = mesenteric fat. Image Séverine Tasker, Bristol Veterinary School, University of Bristol, UK.

**Figure 19:**
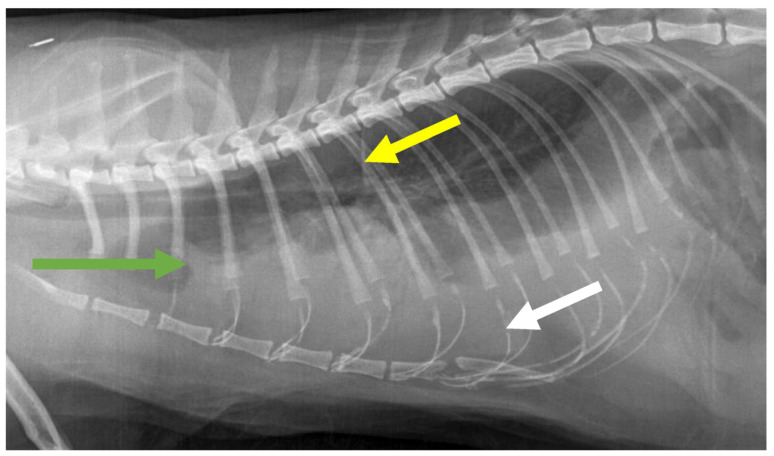
Lateral thoracic radiograph showing the presence of a pleural effusion; Yellow arrow = pleural fissure lines. White arrow = border effacement of the cardiac silhouette. Green arrow = the pleural effusion has displaced air-filled lung dorsally and the lungs are reduced in size. Image Andrew Parry, Willows Veterinary Centre, Solihull, UK.

Ultrasonography (Figure 17 and Figure 18) or radiography (Figure 19) can be used to locate or confirm the presence of effusions and to assist in sample collection [250].

A review of abdominal ultrasonographic findings in 16 cats with FIP [29] showed the presence of peritoneal fluid in 7 cases, and retroperitoneal fluid was found in 1 cat. Abdominal lymphadenopathy was documented in nine cats. The liver was of normal echogenicity in 11 cats and variably hypoechoic or hyperechoic in the remainder. The spleen was of normal echogenicity in most cats and hypoechoic in two. Five cats had hypoechoic subcapsular rims in one or both kidneys. In another study of FIP cases undergoing treatment, 16 of the 18 cats showed lymphadenomegaly on abdominal ultrasonography at presentation [219]. In 22 cats with FIP that underwent ultrasonography, 5 cats had colonic wall thickening [45]. A retrospective ultrasonographic study [284] focused on the significance of the medullary rim sign (MRS) in the kidneys of cats; of 661 cats that had undergone abdominal ultrasonography, 23 cats were diagnosed with FIP; 15 of these had MRS (mostly the thick-marked intensity type) and 8 did not, corresponding to a significant association between the presence of MRS and FIP. A diagnosis of FIP was made by the clinician without details given on diagnostic criteria for FIP. The significance of the association between MRS and FIP is not known, but it is an interesting finding.

Pneumonia due to FIP is occasionally reported and can be associated with thoracic radiographic changes [218]. In a retrospective study of 148 cats with pleural effusion [285], no radiographic variables were found to be predictive of a diagnosis of FIP, although only 2 of the 148 cats had FIP.

**Figure 20:**
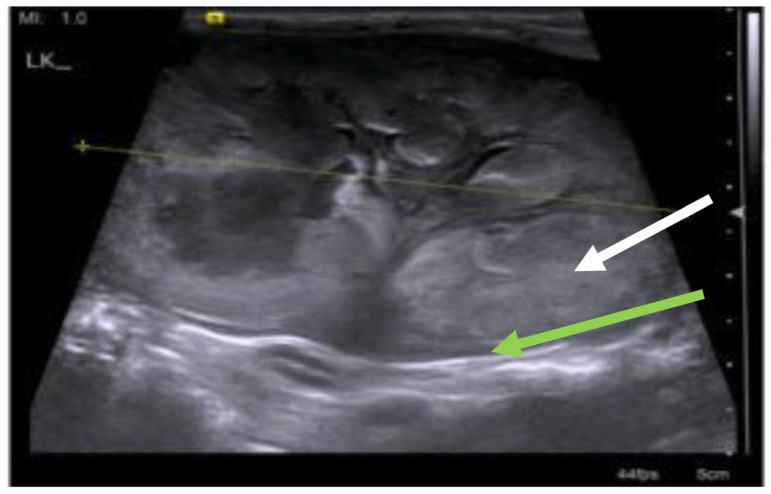
Ultrasonogram of a cat with FIP and renomegaly with a loss of normal renal architecture; ultrasonography might be useful to guide fine-needle aspirate or tissue core-biopsy sampling of organs by targeting abnormal tissue. The kidney is enlarged (50 mm, normal size range is 33–44 mm). White arrow = loss of corticomedullary distinction with heterogeneously echogenic renal parenchyma. Green arrow = pericapsular hypoechoic material is sometimes seen in cases with FIP. However, this is also encountered with other diseases (e.g., lymphoma). Image Séverine Tasker, Bristol Veterinary School, University of Bristol, UK.

It is clear that no specific ultrasonographic or radiographic findings occur in FIP. However, imaging can be useful to find small volumes of effusion to sample and to direct the sampling of abnormal tissues from affected organs (Figure 20) (e.g., collecting FNAs for cytology or collecting ultrasound-guided needle cores (e.g., tru-cut) biopsies for histopathology). Illustrative details on how to find small pockets of fluid and collect FNAs under ultrasonographic guidance are available in the 2022 AAFP/EveryCat Feline Infectious Peritonitis Diagnosis Guidelines [36].

#### 7.4.2. Advanced Imaging of the CNS: MRI and CT

**Figure 21:**
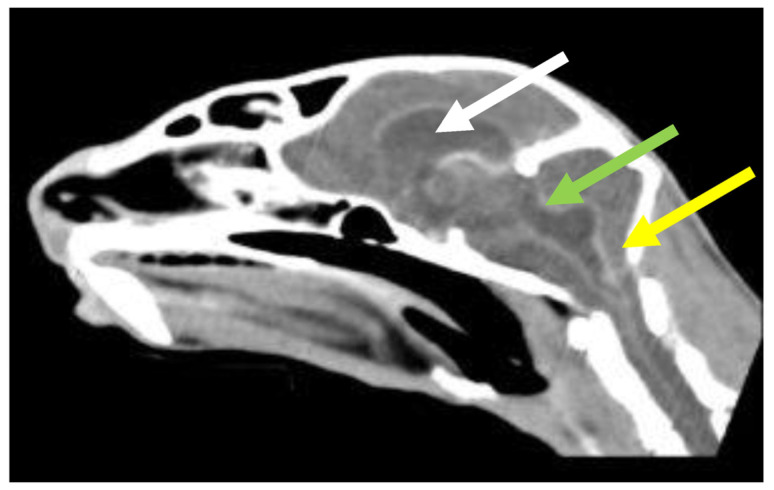
CT of the head of a cat with neurological signs due to FIP post-contrast—reconstructed to show a sagittal midline view. White arrow = there is evidence of generalised ventriculomegaly, suggesting alteration/obstruction to CSF flow. Green arrow = homogeneous contrast enhancement of the lining of the ventricles (ependyma) is sometimes seen in patients with FIP. Yellow arrow = the increased ventricular size in this patient has led to an increase in volume of the contents of the calvarium. This patient has coning of the cerebellum—the cerebellar vermis is beginning to pass through the foramen magnum—a life threatening finding. Image Séverine Tasker, Bristol Veterinary School, University of Bristol, UK.

**Figure 22:**
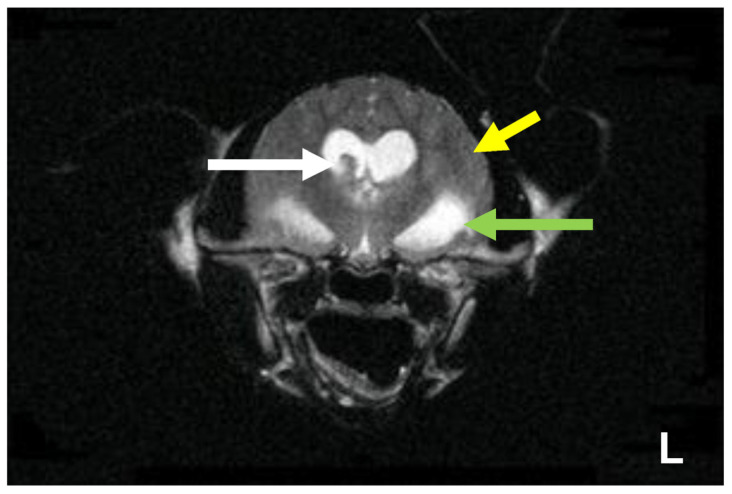
T2W transverse MRI of a cat with neurological signs due to FIP. The brain also appears swollen with a lack of visible sulci. Yellow arrow = the gyri in this patient are enlarged, with narrowing of the sulci. This will be due to parenchymal inflammation. Green arrow = the edge of the left lateral ventricle is identified and appears dilated. White arrow = the T2W isointense (to grey matter) structure is an enlarged right choroid plexus. Image Séverine Tasker, Bristol Veterinary School, University of Bristol, UK.

When a cat is showing neurological signs, imaging the brain by MRI, if available, can be useful to demonstrate neurological abnormalities due to FIP. Obstructive hydrocephalus, syringomyelia, foramen magnum herniation and the marked contrast enhancement of the meninges, third ventricle, mesencephalic aqueduct and brainstem have been reported in cats with FIP [33,35,238,279]. Some cats only show abnormalities after the administration of contrast [35,238], and some cats have normal MRI even after contrast administration, despite the presence of meningoencephalitis [238] A description of CT findings in cats with neurological FIP has not been published, and, although hydrocephalus and/or syringohydromyelia can sometimes be detected by CT, MRI is likely to be more sensitive in the detection of subtle intraparenchymal lesions [286] (Figure 21 and Figure 22). Imaging of the CNS is indicated before performing CSF sampling to assess the potential risk of herniation.

**Summary of Section 7: Diagnosis of FIP; Section 7.4: Diagnostic imaging in FIP:** **No specific ultrasonographic or radiographic findings exist for FIP.** **Ultrasonography (in particular) and radiography** can show the **presence of effusions**. Pneumonia due to FIP that is occasionally reported can be associated with radiographic changes. Ultrasonography can reveal **abdominal lymphadenomegaly or lymphadenopathy** and/or **abnormalities of the liver, spleen, intestines and/or kidneys (which can include a medullary rim sign)**, depending on which organs are affected. **Imaging can also be of use to the direct sampling of abnormal tissues**, e.g., fine-needle aspirate for cytology examination to reveal non-septic pyogranulomatous inflammation, or ultrasound-guided needle core (e.g., tru-cut) biopsies can be collected and submitted for histopathology. When a cat is showing **neurological signs**, the imaging of the brain by **magnetic resonance imaging**, if available, with contrast, can be useful to demonstrate neurological abnormalities (such as obstructive hydrocephalus, syringomyelia, foramen magnum herniation and marked contrast enhancement of the meninges, third ventricle, mesencephalic aqueduct, and brainstem). A description of computerised tomography findings in cats with neurological FIP has not been published, but MRI is likely to be more sensitive in the detection of subtle intraparenchymal lesions. **Advanced imaging of the central nervous system is indicated before performing cerebrospinal fluid sampling to assess the potential risk of herniation**.

### 7.5. Direct Detection of FCoV

#### 7.5.1. Detection of FCoV Antigen

##### Histopathological Examination of Tissues with FCoV antigen Immunostaining

**Figure 23:**
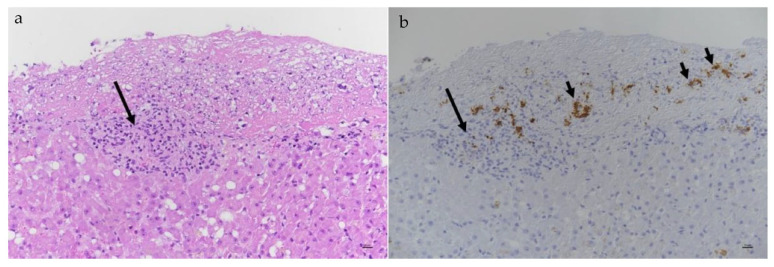
(**a**) Histopathology: Hematoxylin and eosin stain and (**b**) positive FCoV antigen immunostaining in a cat with FIP: liver, fibrinous perihepatitis with embedded FCoV-infected macrophages (shorter arrows) and focal granulomatous infiltrate (longer arrows) with FCoV-positive macrophages. Image Anja Kipar, University of Zurich, Switzerland.

**Figure 24:**
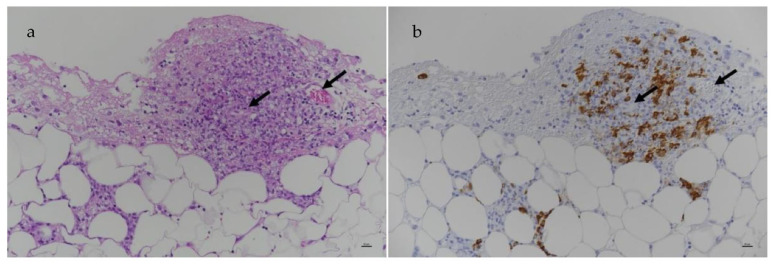
(**a**) Histopathology: Hematoxylin and eosin stain and (**b**) positive FCoV antigen immunostaining in a cat with FIP: Mesentery with focal granulomatous infiltrate with embedded small veins (arrows) and abundant FCoV-positive macrophages. Image Anja Kipar, University of Zurich, Switzerland.

**Figure 25:**
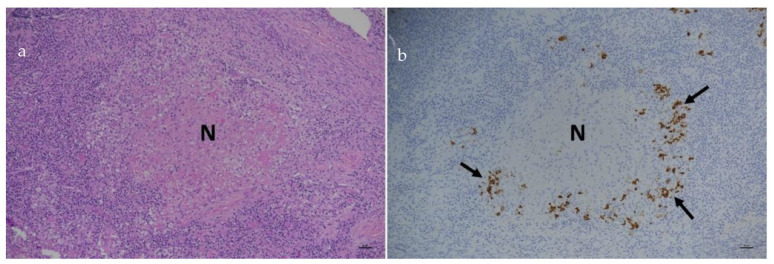
(**a**) Histopathology: Hematoxylin and eosin stain and (**b**) positive FCoV antigen immunostaining in a cat with FIP: Mesenteric lymph node with focal granulomatous infiltrate with extensive central necrosis (N) and abundant FCoV-infected macrophages (arrows) in the surrounded infiltrate. Image Anja Kipar, University of Zurich, Switzerland.

**Figure 26:**
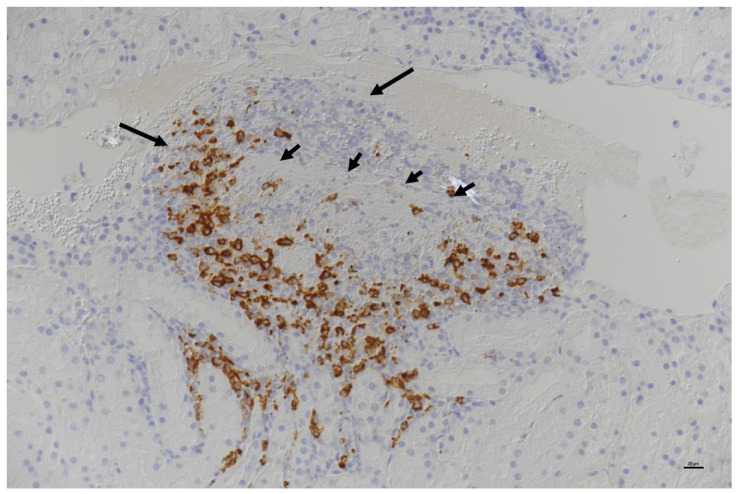
Positive FCoV antigen immunostaining in a cat with FIP: Kidney, stellate vein in subcapsular cortex with granulomatous (peri)phlebitis. Focally, the granulomatous infiltration has destroyed the vascular basement membrane (left arrow), protrudes into the lumen of the vein (wall-bound thrombus; right arrow) and is present in surrounding tissue, containing abundant FCoV-infected macrophages (cells stained in brown). Shorter arrows outline the remnants of the basement membrane. Image Anja Kipar, University of Zurich, Switzerland.

The definitive diagnosis of FIP relies on consistent histopathological changes in affected tissues, and this, with concurrent FCoV antigen immunostaining, is considered the gold standard for diagnosis (Figure 23, Figure 24, Figure 25 and Figure 26).

Immunostaining exploits the binding of antibodies to host-cell-associated FCoV antigens, which are subsequently visualised by enzymatic reactions producing a colour change in a process called IHC. However, care must be taken to ensure that adequate controls are in place for each organ examined since non-specific staining can occur, leading to false-positive results (see below and Section: Cytology with FCoV antigen Immunostaining on Effusions, FNAs, CSF and Aqueous Humour).

The ‘classical’ FIP histopathological lesion is a blood vessel surrounded by an inflammatory lesion dominated by monocytes/macrophages intermingled with a few neutrophils and lymphocytes [6], which are mainly CD4+ [287]. Occasionally, monocytes can be seen attached to endothelial cells or emigrating from the vessel [6]. Periventricular encephalitis and leptomeningitis are commonly seen in neurological FIP [239,288]. A useful study [289] documented the following patterns as being consistent with FIP lesions:

- Pyogranulomas on one or more serosal surfaces;
- Granulomas with or without necrotic areas;
- Lymphocytic and plasmacytic infiltrates in specific sites (e.g., band-like infiltrate in serosal surfaces, perivascular infiltrate in meninges and CNS);
- Granulomatous to necrotising vasculitis and fibrinous serositis.

Histopathology alone is sometimes used to definitively diagnose FIP [290]. In one study analysing 93 tissues from 14 cats with FIP [289], histopathological lesions consistent with FIP were most commonly found in the lungs (77% of samples), then kidneys (64%), MLNs (62%), liver (57%) and spleen (57%). Differential diagnoses for pyogranulomatous inflammation include other infectious diseases (e.g., infections with mycobacteria, actinomyces, nocardia, rhodococcus, pseudomonas [291], toxoplasmosis, bartonella, fungi), as well as rarer idiopathic sterile pyogranulomatous diseases that can present with mass lesions, such as in the lymph nodes (e.g., mesenteric, submandibular) [292] or skin.

However, in addition to histopathological changes, a definitive diagnosis of FIP should rely on the demonstration of positive immunostaining for FCoV antigens within macrophages in the histopathological lesions, such as by IHC [4,289,293]. Positive-FCoV antigen-IHC is highly specific and reliable [239,289,294] as long as it is performed with appropriate controls and reagents that prevent the non-specific binding of the FCoV antibody to the tissues, as otherwise, false-positive results occur. However, the visualisation of the pattern of FCoV antigen staining by an experienced pathologist should discern non-specific staining. Additionally, a negative result does not exclude FIP as FCoV antigens can be variably, and sparsely, distributed within lesions [255,276,289] and might not be detected in all histopathological sections prepared from FIP-associated tissues changes [293]. This may be in part dependent on how acute the disease is [295]. If unexpected negative IHC results are obtained, it is worth requesting additional sections of biopsies to be cut and examined by the pathologist [251,289]. The size of samples, when small, may reduce the sensitivity of IHC, especially if FCoV antigen distribution is sparse. In one study that evaluated hepatic and renal TCB samples collected from cats with FIP (mostly at post-mortem examination) [276], the sensitivity of IHC was only 24% in hepatic samples and 39% in renal samples. However, sampling was random rather than targeted at lesions.

Samples of affected tissues (e.g., liver, kidney, spleen, MLNs) can be collected at post-mortem examination (this used to be common before the availability of effective antiviral treatments for FIP) or in vivo by laparotomy, laparoscopy or ultrasound-guided TCB. Eyes enucleated as a result of uveitis-associated intractable glaucoma or pain can also be submitted for histopathology and IHC [174]. However, ocular tissues characterised by heavy plasmacytic inflammation in FIP are less likely to be IHC-positive for FCoV antigens [245]. The samples most likely to be useful are those that are affected by the disease process, and this can be guided by the results of diagnostic testing (e.g., imaging results, pyogranulomatous inflammation on FNA cytology) as well as clinical signs.

**Figure 27:**
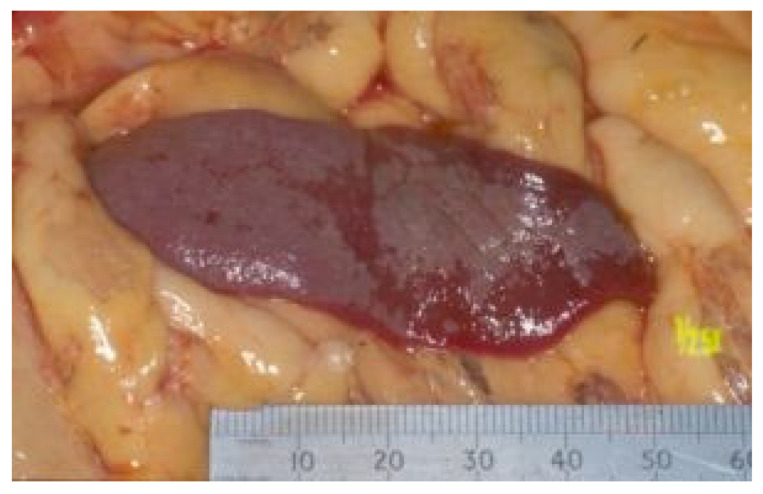
Gross appearance of fibrinous plaque-like inflammation present on the surface of the spleen in a case of FIP with an abdominal effusion that underwent post-mortem examination. Image Séverine Tasker and the Pathology Department, Bristol Veterinary School, University of Bristol, UK.

**Figure 28:**
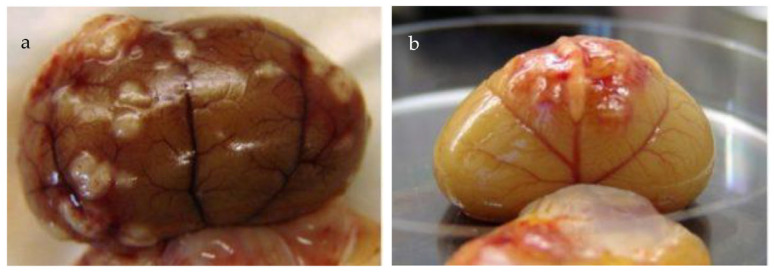
(**a**,**b**) Gross appearance of the kidneys from two cats with FIP, showing renomegaly with pyogranulomas visible on the renal surface on post-mortem examination. (**b**) shows how pyogranulomas can be centred on blood vessels. These lesions could be mistaken for tumours on gross post-mortem examination, which is why histopathology, and ideally, immunohistochemistry is necessary. Images Pathology Department, Bristol Veterinary School, University of Bristol, UK.

If cats are euthanised due to suspected FIP, and without the option to treat the cat with effective antiviral treatment, samples should ideally be collected at post-mortem examination for histopathological examination and FCoV antigen immunostaining (see above) to confirm the disease. Gross findings sometimes are suggestive of FIP [296] (Figure 27 and Figure 28), but lesions might not be obvious. Indeed, it is known that histopathological changes consistent with FIP can be seen in tissues that have not shown macroscopic changes at post-mortem examination [289]. Large pyogranulomatous lesions can also be mistaken for tumours (Figure 28).

##### Cytology with FCoV antigen Immunostaining on Effusions, FNAs, CSF and Aqueous Humour

**Figure 29:**
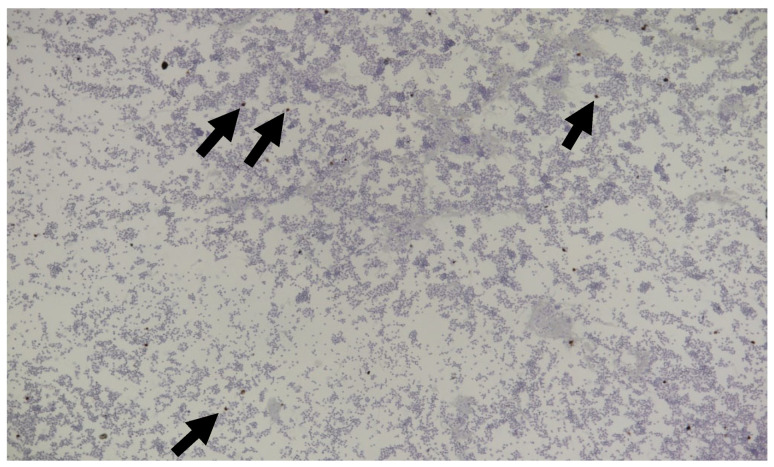
Immunocytochemistry showing the presence of FCoV antigen in macrophages in an effusion of a cat with FIP. Overview at 40× magnification, cytospin, scattered positive macrophages clearly visible at low power (e.g., black arrowheads) before cells further identifiable. Image Alex Malbon and Anja Kipar, University of Zurich, Switzerland.

**Figure 30:**
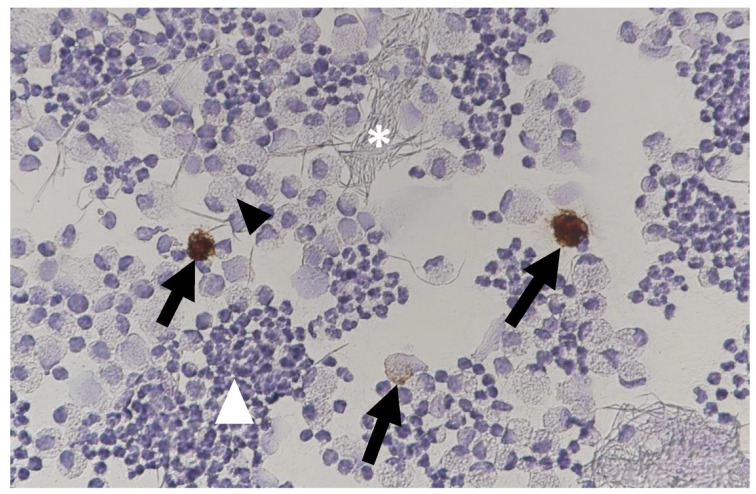
Immunocytochemistry showing the presence of FCoV antigen in macrophages in an effusion of a cat with FIP. 400× magnification, cytospin, highly cellular—macrophages (black arrowhead), neutrophils (white arrowhead) and fibrin (white asterisk). Scattered positive macrophages (black arrows). Image Alex Malbon and Anja Kipar, University of Zurich, Switzerland.

FCoV antigen immunostaining can be performed on cytology samples such as effusions, FNAs, CSF and aqueous humour, using immunocytochemistry (ICC) or immunofluorescence (IF). Host-cell-associated FCoV antigens, in macrophages, are detected with FCoV-specific antibodies conjugated with enzymes or fluorescent markers. The presence of FCoV antigens can then be demonstrated by either enzymatic reactions producing a colour change (see Figure 29 and Figure 30) or by the visualisation of fluorescence using a UV microscope, respectively. The varied methods that have been used for the detection of FCoV antigens within macrophages has led to variability in the reported specificities for immunostaining in cytology samples.

The FCoV immunostaining of effusion samples has shown variable sensitivity from 57 to 100% [14,249,273,297,298,299,300], depending on the methodology used. Since this technique relies on staining FCoV within macrophages in the effusion, false-negative results can occur [301], especially if the effusion is cell-poor and/or the FCoV antigen is masked by FCoV antibodies in the effusion.

FCoV immunostaining is generally considered to be very specific on effusions. Direct techniques that have used a single antibody, conjugated to a fluorochrome, have consistently demonstrated 100% specificity in different laboratories [14,249,273,298,300]. In contrast, indirect methods on effusions using avidin and biotinylated horseradish peroxidase complexes and secondary antibodies [297], or direct methods in which two antigens are targeted with the application of two antibodies [299], have reported lower specificities of 72% and 71%, respectively. In the former study, eight (three cats with heart failure and five cats with neoplasia) of twenty-nine non-FIP effusions were found to be positive by ICC [297], whilst in the latter study, two of seven non-FIP effusions (one of the two cats had heart failure, the other cholangiocarcinoma) were found to be positive by IF [299]. Whilst a multiplex fluorescent ICC assay utilising dual antibodies (vimentin and FCoV) has been developed [302], specificity and sensitivity data using this technique are yet to be determined. It is therefore important for clinicians to be aware of variations in immunostaining techniques and to be familiar with the specificity of the methodology employed by their local laboratory, as well as confirmation of the inclusion of negative controls in testing when interpreting positive results.

Some diagnostic laboratories prefer to use cell pellets from centrifuged effusion samples to prepare formalin-fixed, paraffin-embedded samples that can then be treated like a tissue specimen for FCoV antigen IHC [293] or IF [301]. This might improve the reliability of the detection of FCoV antigen [293], although the processing time required for these samples would be longer than for direct cytological immunostaining (or for RT-PCR testing; see Section on FCoV RT-PCR on Effusions). The larger the effusion volume sample is, the better the sensitivity is likely to be, probably due to a larger cell harvest. This was illustrated in a single case report [41], although samples were collected at different time points making comparisons difficult. Further studies are required to confirm the advantage of larger volume samples.

The FCoV immunostaining of FNA samples has not yet been described in large comprehensive studies, although it has been reported in small numbers of cats [45]. One study that performed hepatic and renal FNA ICC in cats with FIP (samples were collected randomly from these organs mostly at post-mortem examination) [276] reported a sensitivity of only 17% to 31% in hepatic FNAs and 11% to 20% in renal samples.

If collecting FNAs for immunostaining, the diagnostic laboratory can be contacted to find out how they would like samples prepared for submission. Some might request cytospins, if available, whilst others ask for several FNAs to be placed into a tube containing a small amount of saline (or autologous serum) until the solution is cloudy and then submitting this to the laboratory; the cloudiness crudely indicates adequate cell presence. Others might request that the cloudy solution of FNAs is centrifuged, and the resulting cell pellet fixed by adding 2 mL of buffered formalin and agitating or vortexing before submission for immunostaining. Note that different sample preservations may be required for FNA samples being submitted for FCoV RNA detection (see Section on FCoV RT-PCR on Tissue and FNA Samples).

FCoV immunostaining using ICC has been reported as being successful in detecting FCoV in the CSF of a cat with neurological FIP [235]. One study evaluated ICC in the CSF of cats with and without FIP that presented with and without neurological signs, collected at post-mortem examination [303]. This study found that 17 of 20 cats with FIP gave positive ICC results, but of 18 cats without FIP, 3 also had positive results (1 cat each with mediastinal lymphoma, lymphocytic meningoencephalitis and hypertensive angiopathy with brain haemorrhage), limiting the test’s specificity. The reasons for the positive ICC results in these three cats without FIP are not known, but suggested possibilities include the concurrent presence of FIP alongside the other confirmed diseases present (although the IHC staining of neurological tissues was also negative), the detection of the presence of systemic FCoV antigens in the absence of FIP or non-specific staining, aberrant antibody binding, and other methodology reasons. The analyses in this study [303] excluded those cats that had no cells present in their CSF as ICC could not be performed on these cats. The same group [280] performed CSF ICC on two cats with neurological signs that did not have FIP, and although one of these was positive, the cytology of the CSF was lymphomonocytic, which would not have been consistent with a diagnosis of FIP. This same study also performed CSF ICC on seven cats with confirmed FIP—three with neurological signs and four without. Two of the three cats with neurological FIP were ICC-positive whilst three of the four cats with non-neurological FIP were also ICC-positive. Most of the ICC-positive results in the FIP cats in this study showed pyogranulomatous cytology in the CSF, consistent with FIP. The application of ICC to CSF samples collected ante-mortem from a larger number of cats with neurological signs due to FIP and other causes would be desirable to further evaluate the usefulness of CSF ICC.

The use of FCoV antigen immunostaining has also been described in aqueous humour samples collected directly following euthanasia from 26 cats with confirmed FIP and 13 cats with other diseases [304]; most (25 with FIP and 11 with other diseases) of these cats were also included in a subsequent study describing both FCoV RT-PCR and FCoV antigen immunostaining in cats with FIP (31 cats) and cats with other diseases (27 cats) [305]. These two studies reported sensitivities of 64% [304] and 63% [305] for aqueous humour FCoV antigen immunostaining, but most of the cats with FIP in these studies did not have ocular signs. The specificities were 82% [304] and 80% [305], with positive results occurring in one control cat with lymphoma and one control with a pulmonary adenocarcinoma (in both cats, the aqueous humour cytology was not consistent with FIP). Accompanying cytology is important to aid interpretation, as is ensuring the laboratory performing the FCoV antigen immunostaining has robust methods and controls. Aqueous humour as a target sample to test is interesting as it can be collected non-invasively from cats with suspected FIP, although the sample collection technique used in the published studies [304,305] might need modification for use ante-mortem (e.g., use of a smaller 27–29 gauge insulin needle) [283]. The further evaluation of ICC on aqueous humour samples collected ante-mortem from cats with uveitis due to FIP and other causes is needed to further assess the usefulness of ICC in the diagnosis of FIP.

##### Detection of FCoV Antigen in Faeces by Rapid Immunomigration Tests

One study has been published [129] evaluating three rapid immunomigration (also known as lateral flow) tests to detect FCoV antigens in the faeces of cats from shelters. The tests were compared to the results of faecal RT-PCR and showed poor sensitivity (21% to 66%) but reasonable specificity (73% to 100%) [129]. The study concluded that the tests had too poor a sensitivity to be used to identify cats shedding FCoV in their faeces. Moreover, the detection of FCoV in faeces (either antigen, as here, or RNA detection by RT-PCR, see Section on FCoV RT-PCR on Faecal Samples) should never be used to diagnose FIP as it is known that, based on RT-PCR studies, cats with FIP do not always shed FCoV, and cats in multi-cat households without FIP commonly shed FCoV (Table 1).

#### 7.5.2. Detection of FCoV RNA by RT-PCR

In general, PCR is a method by which DNA is exponentially amplified by a polymerase enzyme with the use of primers (and a probe in quantitative PCR assays) to target a specific sequence, enabling sensitive detection down to a very low starting DNA copy number. Post-PCR amplification processing (e.g., sequencing) can be applied if needed.

PCR only amplifies DNA; because FCoV is an RNA virus, a pre-PCR step using a viral enzyme, reverse-transcriptase (RT), is required to generate a strand of cDNA using the original FCoV RNA template, in a process known as reverse transcription. A combination of this process and PCR is known as RT-PCR [54]. The RT-PCR assays available to detect FCoV RNA often amplify both cell-associated subgenomic mRNA (RNA produced in feline cells when the FCoV replicates), as well as cell-associated and virus particle-associated genomic RNA (which correlates to the presence of whole FCoV). Where in the FCoV genome the PCR primers bind to determines whether subgenomic mRNA is preferentially amplified in an RT-PCR assay [54,306]. Those RT-PCR assays that favour the amplification of subgenomic mRNA might overestimate the FCoV viral loads present in the sample [54]. Laboratories should be able to report the analytical sensitivity and specificity of their RT-PCRs and also provide details of the positive and negative controls that they use. Usually, highly conserved areas of the FCoV genome are targeted in RT-PCR assays to maximise sensitivity. As an RNA virus, FCoV shows a high rate of error during replication, and any mutations at the site of primer and/or probe binding can result in a loss of RT-PCR assay efficiency, and ultimately sensitivity. PCR assay conditions (e.g., temperature) can be altered to tolerate such mutations, but this can reduce specificity [306]. Additionally, RT-PCRs designed to target type I FCoV, which represents the majority of field strains found in naturally infected cats (although geographical variation exists [74,76]), might not amplify type II FCoV if the primers and probe bind to the region of the FCoV genome that differs between the two types (i.e., around the spike (S) protein, Figure 3) [58,62,67].

FCoV RT-PCR has been used to detect FCoV RNA in blood, effusion, tissue (including FNAs), CSF, or aqueous humour samples from suspected cases of FIP, with varying results. Bronchoalveolar-lavage FCoV RT-PCR was also used to support a diagnosis of FIP in one cat with respiratory signs but no effusion [212].

Ideally, RT-PCR assays used should be quantitative (this is the ‘q’ in RT-qPCR) and be able to report the FCoV load present in the sample, because this information is an important aid to the interpretation of results. This is because systemic FCoV infection (viraemia) can occur in healthy cats and cats without FIP, as well as in cats with FIP. However, the FCoV viral loads in healthy cats and cats without FIP are lower than those in cats with FIP [7,84,85,307]. So, a positive RT-qPCR result is not specific for FIP, but positive RT-qPCR results with a high FCoV load strongly support a very likely diagnosis of FIP.

Running FCoV RNA RT-PCRs can be rapid, although, once the time taken to submit the sample to the laboratory is factored in, the reporting of results can still take a few days. This is usually quicker than FCoV antigen immunostaining on tissue biopsy samples and often also quicker than immunostaining on effusion samples. Rapid molecular techniques (e.g., RT loop-mediated isothermal amplification; LAMP) for detecting FCoV RNA in-house as point-of-care tests have been described [308,309,310]. These show some promise but have traditionally suffered from poor sensitivity, and further work on clinical samples is required before they can be recommended.

##### FCoV RT-PCR on Blood Samples

Samples derived from blood (e.g., whole blood, serum, plasma, or peripheral blood mononuclear cells [PBMCs]) can undergo RT-PCR for FCoV RNA detection following RNA extraction. When FCoV RT-PCR was performed on plasma or serum samples from cats with and without FIP in various studies [290,311,312], only 0% to 15% of FIP cases were positive for FCoV RNA, and none of the cats without FIP gave positive FCoV RT-PCR results. Although whole blood or PBMCs might be better targets for RT-PCR than serum [311], FCoV RNA was only detected in the whole blood of 2 of 18 (11%) cats with FIP 14 days after experimental infection [313]. One study was more successful in detecting FCoV RNA in blood samples from cats with FIP [256] when RT-PCR was applied to pellets derived from whole blood; positive results were obtained in six of eight (75%) cats with FIP, but none of eight cats with diseases other than FIP. Similarly, another study [24] documented that 15 of 18 (83%) cats with confirmed or highly suspected FIP were positive by RT-PCR for FCoV RNA in whole-blood samples. Finally, one study that tested the whole blood of 125 cats with suspected effusive FIP by RT-PCR [19] found 114 (91%) to be positive, whilst a similar study on cats with non-effusive, or ‘mixed’ effusive/non-effusive FIP, found 138/156 (89%) and 124/153 (81%) of blood samples, respectively, to be positive [31]. However, a positive blood sample RT-PCR result was one of the criteria used to deduce a diagnosis of FIP in these studies [19,31], likely biasing the numbers reported.

Interestingly the study [24] that found FCoV RNA in whole-blood samples from 83% of 18 cats with FIP used the same RT-PCR assay [based on the 7b gene of the FCoV genome [314]] as a previous study, which documented only 11% positive RT-PCR results in blood samples from 18 cats with FIP [313]. The reason for the discrepancy between these sensitivity results is not known and needs further investigation; for example, it may be due to sample collection, processing, or storage conditions.

The specificity of FCoV RT-PCR on blood samples is also an issue, as healthy and ill cats without FIP can have detectable FCoV RNA in the blood, albeit uncommonly. One study [83] found that 9 of 205 (4%) healthy USA shelter cats were FCoV RNA RT-PCR-positive in buffy coats prepared from blood; 1 of those had a replicating virus in the bloodstream, as demonstrated by a positive FCoV mRNA RT-PCR result, and this 8-week-old kitten was likely undergoing viraemia. Neither this kitten, nor seven of the nine FCoV RNA RT-PCR-positive cats with follow-up available, developed FIP during the subsequent six months. Another study [315] performed FCoV mRNA RT-PCR on PBMC samples to detect replicating FCoV and found that 23 of 424 (5.4%) samples from cats without clinical signs of FIP were positive, compared to 301 of 651 (46.2%) samples from cats with clinical signs suggestive of FIP. The cross-reactivity of this FCoV mRNA RT-PCR with human DNA has been suspected (Diane Addie, personal communication).

The more recent results obtained with FCoV RT-PCR on blood samples make it an interesting avenue to explore as a test to support a diagnosis of FIP.

##### FCoV RT-PCR on Effusions

Effusion samples in cats with FIP often contain FCoV RNA [313], which can be detected by RT-PCR. The centrifugation of the effusion sample to yield a cell pellet to use for RNA extraction may improve sensitivity [301]. Published studies amplified FCoV RNA in most (72–100%) effusion samples from cats with confirmed or suspected FIP [19,31,256,311,312,316] but usually not in any effusions from cats without FIP [311,312,316]. However, subsequent studies have challenged the specificity of RT-PCR on effusions. One study [106] amplified FCoV RNA, albeit at a low level, in abdominal fluid from 1 of 29 control cats that did not have FIP. Another study [290] amplified FCoV RNA from three (two of the three had only low levels of FCoV RNA) of twenty-four control cats without FIP that had effusions tested. In the latter study, the control cats that generated positive FCoV RT-PCR results had neoplasia (lymphoma and a malignant round-cell tumour) or chronic kidney disease (this cat had the higher FCoV RNA levels in the effusion). Another study [256] amplified FCoV RNA (levels not reported) from the effusion of one cat with intestinal carcinoma (out of six control cats with effusions tested). Finally, one study [301] documented a specificity of 81% for RT-PCR on effusions as positive RT-PCR results were obtained in 3 of 16 samples from cats without FIP; however, confirmation of the absence of FIP in these 3 cats was only based on the negative IF immunostaining of effusions from the cats, and it might well be that these 3 cats did indeed have FIP.

Thus, the presence of FCoV RNA, particularly in high levels, in an effusion that also has cytological and biochemical features suggestive of FIP, makes FIP a very likely diagnosis (Figure 12) [17], and this might be adequate information upon which to start trial treatment for FIP now that effective antiviral treatments such as GS-441524 [20,24] are increasingly available (see Section 10 on Treatment of FIP).

##### FCoV RT-PCR on Tissue and FNA Samples

When tissue biopsy samples are obtained from cats with suspected FIP, the samples should be submitted for histopathology and IHC, as this allows for a definitive diagnosis of FIP. However, if a delay in analysis is expected, tissue could be submitted for RT-PCR, as finding high levels of FCoV RNA in a sample of an affected organ can allow us to make a diagnosis of FIP very likely. This is because it is known that tissue samples from cats with FIP are significantly more likely to be FCoV RT-PCR-positive [106,256] and have significantly higher FCoV RNA loads in RT-PCR [107] than tissue samples from cats without FIP. In cats with FIP, FCoV RNA loads correlate with histopathological findings suggestive of FIP [106,313]. In one study that included 20 cats with FIP confirmed by IHC, 70–90% of incisional biopsies of popliteal and MLNs, liver, spleen, omentum, and kidneys were found to be positive by RT-PCR [255].

However, it is important to remember that cats without FIP can also be found to be positive for FCoV RNA by RT-PCR in tissues. One large study evaluating FCoV RT-PCR in 260 tissue samples from 57 cats with FIP, and 258 tissue samples from 45 cats without FIP [106], found that 90% of tissue samples from the 57 cats with FIP were FCoV RT-PCR-positive, but 8% of tissue samples from 45 cats without FIP were also FCoV RT-PCR-positive. Another larger study performed FCoV RT-PCR on 1861 samples from 87 cats without FIP and found that 24% (21/87) of the cats were FCoV RT-PCR-positive on at least one tissue or fluid sample besides faeces, and 4% (78/1861) of all of the samples tested were FCoV RT-PCR-positive [254]. Interestingly, only 1 of the 87 cats without FIP was found to be faeces-positive by RT-PCR [254].

It is recommended that tissue samples are not formalin-fixed before RT-PCR, as formalin can degrade RNA and decrease PCR sensitivity [251], although one study has described the successful use of FCoV RT-PCR in formalin-fixed paraffin-embedded tissues in cats with FIP [317].

FNAs, such as those obtained by ultrasound guidance, are often a good alternative to tissue samples for FCoV RT-PCR analysis, as they have the advantage of less-invasive collection. One study (although in abstract form only [318]) described the successful amplification of FCoV RNA from ultrasound-guided FNAs of abnormal tissues (tissue type not specified) in all 11 cats with FIP without effusions that were sampled, suggesting that FNAs could be a useful sampling material for RT-PCR in cats with FIP that do not have effusions. Indeed, positive RT-PCR results on MLN FNAs collected at either ante-mortem or post-mortem examination were reported in 18 of 20 (i.e., sensitivity of 90%) cats with FIP but without effusions [30]; in this study, the 2 cats with FIP that were negative on MLN FNA RT-PCR were neurological FIP cases. This study controlled and evaluated cats without FIP too, detecting MLN FCoV in only 1 of the 26 cats without FIP (i.e., specificity of 96%) [30]. In this study, FCoV RNA survived well in transport as some of the FNAs tested by RT-PCR were sent by regular mail without ice or RNA preservative [30]. The successful use of FCoV RT-PCR on MLN FNAs collected ante-mortem to diagnose FIP has also been described in a descriptive study of a small cohort of cats with FIP [43].

A study of 20 cats with FIP compared the RT-PCR results on FNAs and incisional biopsies of popliteal and MLNs, liver, and spleen. Percentages of positive RT-PCR results were similar for FNA (65% to 85%) and incisional biopsy (70% to 90%) samples [255], suggesting FNAs to be a good sample source for RT-PCR testing, especially as they can usually be collected relatively non-invasively, as mentioned earlier.

If collecting FNAs for RT-PCR, it is good practice to consult the diagnostic laboratory for information on how they would like samples prepared and/or preserved for submission, to ensure optimal sensitivity. For example, the laboratory may request that several FNA aspirates are added to a sample tube containing a small amount of saline until the solution becomes cloudy, crudely indicating an adequate presence of cells in the sample to be submitted. Others may require you to submit FNAs in an RNA transport medium.

Positive FCoV RT-PCR tests on samples from abnormal tissues, with consistent cytology, may be adequate to make FIP a very likely diagnosis (Figure 12), enabling the start of trial treatment for FIP, now that effective antiviral treatments such as GS-441524 [20,24] are increasingly available (see Section 10 on Treatment of FIP).

##### FCoV RT-PCR on CSF Samples

Samples of CSF can be submitted for FCoV RT-PCR. Studies have described the use of FCoV RT-PCR on CSF samples, but sensitivity has been poor, at only 30% [280], 31% [35], 41% [233] or 50% [106] in cats with FIP. However, not all cats included in these studies had neurological signs, as CSF was collected at post-mortem examination independent of presenting signs [106,233,280], such that the population tested does not necessarily reflect a population of cats with neurological signs that would have had CSF samples collected for diagnostic purposes. Indeed, in one study [233], the sensitivity of RT-PCR rose from 41% to 86% when only cats with neurological and ophthalmological signs of FIP were considered. The same group found similar findings in a larger number of cats [280], where the sensitivity of RT-PCR was only 30% when both neurological and non-neurological FIP cases were included, but this rose to 83% when only cats with neurological FIP were included in statistical analysis.

The specificity of FCoV RT-PCR on CSF samples is good, with values of 100% (i.e., no false-positives) reported in two studies of 15 [233] and 29 [280] cats without FIP. However, FCoV RNA has occasionally been found in the CSF of cats without FIP—in 1 (of 87 cats) without FIP that had disseminated lymphoma [254] and in 2 persistently infected FCoV carrier cats without FIP (Diane Addie, personal communication).

In one study [319], all CSF samples with a CSF FCoV antibody titre of greater than 640 that were tested for FCoV RNA were found to be positive by RT-PCR. This study was limited by the fact that FIP was not confirmed in all cats, but it does suggest an association between high CSF FCoV antibody titres (see Section on Antibody Testing on CSF Samples) and positive CSF FCoV RT-PCR.

Thus, FCoV RT-PCR on CSF appears to be a useful additional test in cats with neurological signs, as a positive result highly supports a very likely diagnosis of FIP, but a negative result does not rule out FIP.

##### FCoV RT-PCR on Aqueous Humour Samples

Positive FCoV RT-PCR results have been reported on aqueous humour samples in cats with FIP [106,255], though samples were collected at post-mortem examination. One study also described positive results in two cats on samples collected ante-mortem [283]. A further study [305] documented positive FCoV RT-PCR on aqueous humour samples from 11 of 31 (36%) cats with confirmed FIP and none of 27 control cats without FIP. Again, these samples were collected at post-mortem examination and, interestingly, only 4 of the 31 cats with FIP had ocular signs of uveitis, and only 2 of these 4 cats were FCoV RT-PCR aqueous humour-positive. Although FCoV RT-PCR had a specificity of 100% in this study, its sensitivity was low, at 36%. Further studies are required on aqueous humour samples collected in vivo in cats with ocular signs consistent with FIP.

##### FCoV RT-PCR on Faecal Samples

FCoV RT-PCR can be performed on faecal samples or rectal swabs, although faecal samples are preferred due to their higher FCoV load for RT-PCR detection compared to swabs [94]. Faecal RT-PCR is sometimes used to identify cats that are shedding FCoV for the management of infection in a multi-cat household but should never be used to diagnose FIP as it is known that the faeces of cats with FIP are not always FCoV RT-PCR-positive and those of cats without FIP in multi-cat households are commonly positive (Table 1).

In studies, the percentage of cats with FIP with positive faecal FCoV RT-PCR results have varied: from 33% (on day 0 of a treatment study, although this rose to 61% when all three faecal samples from the first three days of the study were included in the analysis) [94] to 35% [112], 65% [106], 81% [107] and 87% [166]. The percentages of cats without FIP with positive FCoV RT-PCR results on faecal samples have also varied: in only 1 of 5 (20%) ill UK cats [166], 56% of 50 healthy cats in USA shelters [83], 60% of 10 ill cats without FIP in the UK [107], 71% of 82 healthy cats from German catteries [135] and 77% of 179 cats from German breeding catteries [123].

Although one study [106] showed that cats with FIP were more likely to be shedding FCoV in their faeces than cats that were euthanised due to diseases other than FIP, in an individual cat, faecal RT-PCR is not useful for the diagnosis of FIP.

#### 7.5.3. Molecular Techniques Characterising FCoV Spike (S) Gene Mutations following Positive RT-PCR for FCoV RNA

Following the detection of FCoV RNA in a sample by RT-PCR, varied molecular techniques can then be used to derive or deduce sequence data for the S gene of the FCoV detected. As described earlier in Section 2.4 on FCoV Pathotypes and Genome Mutations, the S gene codes for the spike protein, which mediates host-receptor recognition and membrane fusion. It has been a target for distinguishing FIP-associated FCoV from less-virulent FCoV [67,91].

Methods to determine FCoV sequences include sequencing methods, such as pyrosequencing and Sanger sequencing, most often used in research, and methods designed to detect and quantify specific mutation sequences, such as the commercially available PCR that uses allelic discrimination to detect M1058L and S1060A mutations. However, it is known that it is not a single, nor just a few, mutations that define the FIP pathotype; many are likely to be involved in the development of FIP [10]. Thus, a test based on the identification of one, or just a few mutations, can never be diagnostic for FIP.

Sequence determination methods are described elsewhere [54,320], but, in brief, the sequencing methodologies used in FCoV mutation assays are:

- Sanger sequencing: a DNA sequencing approach that uses the dideoxy chain termination method to sequence a segment of the S gene of FCoV [301].
- Pyrosequencing: a DNA sequencing approach to the S gene that is based on the sequencing-by-synthesis principle [316].
- Allelic discrimination: an approach available commercially that uses probes in a qPCR to determine if two specific mutation SNPs (M1058L and S1060A; Figure 1) are present in the FCoV S2 fusion domain.

However, these techniques are not always successful in deriving the FCoV sequence data in FCoV RT-PCR-positive samples. One reason for this is that the FCoV levels can be too low to allow sequence analysis, particularly in cats without FIP where FCoV levels are typically low [254]. Alternatively, sequencing techniques that target the detection of particular sequences (e.g., allelic discrimination) might not be able to generate sequence data due to mismatches or sequence variability in the FCoV S-gene sequences in the sample, and some sequence analysis methods only detect S-gene mutations in type I FCoV, and not type II FCoV [67,106]. Indeed, studies using sequencing to derive S1/S2 sequence data in cases of FIP have shown novel mutations [109,110], different mutations within the same cat [55], or novel mutations in the S2 fusion domain [94], highlighting the variability in sequence that can be present in the S gene, which limits application of methods targeting a specific sequence.

Sequence analysis has usually focused on the S2 subunit fusion-domain region of the S gene of type I FCoV, in which specific mutations (M1058L and S1060A) were found in the FCoV in tissues from cats with FIP but not in the FCoV in the faeces of healthy cats without FIP [67,91,94] (Figure 1). Sanger sequencing studies found evidence of S-gene mutations in the effusion and blood samples of cats with FIP but not in their faeces, nor in the faeces of companion cats that lived with the cats with FIP [94]. Other studies [106,107] analysed the FCoV of both tissue and faecal samples from cats with FIP and cats without FIP (confirmed as having diseases other than FIP by histopathology) by pyrosequencing, followed by Sanger sequencing if pyrosequencing was not successful. These studies [106,107] found that these spike M1058L and S1060A gene mutations were also found in the FCoV in tissues from cats without FIP, leading the authors to conclude that these mutations are associated with systemic FCoV infection, rather than FIP, per se. However, other studies have failed to find mutations in tissue samples from FCoV-infected cats without FIP. For example, Sanger sequencing studies on a selection of samples from cats without FIP that had tested FCoV RT-PCR-positive found no evidence of S-gene mutations in any of 16 samples tested [254]. Although this might be expected in samples derived from the intestinal tract (including faeces), eight of the samples were from the mesenteric and popliteal lymph nodes and the kidneys, where one might expect to find the mutations if they were associated with systemic spread of non-FIP-associated FCoV [254].

One study described a cat with neurological FIP [109] in which histological changes of FIP were found only in the CNS. Upon sequencing, the FCoV in the CNS had S-gene mutations (including a functionally relevant R793M mutation in the S1/S2 cleavage site), whereas the FCoV found systemically in other organs did not. Another study, sequencing the same S1/S2 area of the S gene [55], reported the presence of both mutated and non-mutated FCoV within the same tissues of cats with FIP. Additionally, a study of seven cats that remained healthy following experimental infection with FCoV [321] was set up to document the presence or absence of S-gene mutations in samples of tissue (primarily colon, liver, thymus) and faeces obtained from these cats; however, S-gene sequences could only be obtained in five samples (four colonic, one liver) from four of the seven healthy cats, and none of these contained the targeted S-gene mutations of M1058L or S1060A.

Overall, the results from these sequencing studies have varied. Although S-gene mutations are likely to be important in the development of FIP, as they can occur in FIP-associated FCoV, the variability of the presence of S-gene mutations in samples from cats with and without FIP suggest that other viral (including other mutations) and host factors can allow effective and sustained replication in monocytes, as well as the activation of infected monocytes, in cats that develop FIP following systemic FCoV infection [293]. One study [10] suggested that because multiple mutations were believed to be involved in the development of FIP, future diagnostic tests may evaluate a combination of sites within the S gene and generate a ‘risk-score’ assessment to aid in the diagnostic process for FIP (e.g., the more mutations identified, the higher the likelihood of FIP development). No such tests are yet available. A further discussion of the results, including sensitivity and specificity, of mutation analysis in different feline sample types, occurs in the next two sections: Diagnostic Use of S-Gene-Mutation Analysis on Tissue Samples and on Diagnostic Use of S-Gene-Mutation Analysis on Effusion and Other Fluid (e.g., CSF, Aqueous Humour) Samples.

##### Diagnostic Use of S-Gene-Mutation Analysis on Tissue Samples

An extensive study [106], which included 260 tissue samples from 57 cats with FIP and 258 tissue samples from 45 cats without FIP, calculated that S-gene-mutation analysis using pyrosequencing (with or without Sanger sequencing) on tissues, as an additional step in the detection of FCoV RNA alone by RT-qPCR, only slightly increased specificity for the diagnosis of FIP—from 93% to 95% (this difference was equivalent to five tissues)—but moderately decreased sensitivity from 90% to 81% (difference equivalent to 20 tissues). The decrease in sensitivity was because of the detection of non-mutated FCoV in cats with FIP (four samples), the presence of type II FCoV in cats with FIP (which was not detected by the mutation analysis assays used that relied on finding the specific S-gene mutations seen in type I FCoV by targeted analysis) (12 samples), and an inability to sequence the FCoV S gene due to only low FCoV copy numbers being present (four samples). The increase in specificity was due to the detection of non-mutated FCoV in cats without FIP (two samples) and an inability to sequence the FCoV S gene due to low FCoV copy numbers (three samples).

Another study [317], which performed S-gene-mutation analysis using a commercially available allelic discriminative assay on pooled tissue samples (5 per cat) from 34 cats with FIP and 30 cats without FIP, reported a much higher specificity of 100% for S-gene-mutation analysis. In this study, only 3 of the 30 cats without FIP were FCoV RT-PCR-positive, and in none of these was S-gene-mutation analysis successful. Thus, it is important to note that the specificity value of 100% was not based on detecting non-mutated FCoV in cats without FIP. The sensitivity of S-gene-mutation analysis [317] was moderate at 71%, as only 24 of the 34 FIP cases had mutations successfully detected.

One further study [255] performed S-gene-mutation analysis using the commercially available allelic discrimination assay on FNAs and incisional biopsies of popliteal and MLNs, liver, spleen, omentum, and kidneys in 20 cats with FIP confirmed by IHC. FCoV containing S-gene mutations was present in at least one sample from each cat, but there was variation in which sample was positive. FCoV with mutations in the S gene was most frequently found in effusions (64%), followed by in incisional biopsies of the spleen, omentum, and kidney (50%), then in MLN incisional biopsies and FNAs (45%), and finally in FNAs of spleen and liver and liver incisional biopsies (40%). There was a loss in sensitivity in all tissues when compared to RT-PCR for FCoV alone, without mutation analysis.

Another study by the same group [254] performed the same commercially available S-gene-mutation analysis using allelic discrimination on FNAs and incisional biopsies from popliteal and MLNs, liver, spleen, omentum, kidneys, lung, intestines (duodenum, jejunum, ileum, colon) and fluid (e.g., CSF, aqueous humour, effusion or lavage fluid), and faecal samples from 21 of 87 cats without FIP that had generated positive FCoV RT-PCR results in at least one sample; 14 of the 21 cats showed mutated FCoV on the commercial allelic discrimination assay, whilst the remaining 7 samples had FCoV RNA loads that were too low to generate results. The group went on to sequence a number of these mutated FCoV samples by Sanger sequencing and, remarkably, none of them contained the S-gene mutations [254]. The results of this study suggest that the commercial allelic discrimination assay is incorrectly identifying FCoV with mutations in samples that do not contain the mutation, markedly questioning the assay’s performance.

Another mutation-analysis study using sequencing [256] on tissues (MLN, spleen, small intestine and lung) in 10 cats with confirmed FIP and eight cats with diseases other than FIP, reported a sensitivity of 70% (seven of 10 cats with FIP had mutations) and specificity of 88% (one of eight cats without FIP had a mutation) compared to values of 91% and 50% respectively for RT-PCR alone.

Finally, a Canadian study [322] that also used sequencing to deduce FCoV S-gene segment sequences, documented that only nine of the 20 (45%) S-gene sequences that could be obtained from 69 tissue samples showing typical histopathological findings of FIP possessed the S-gene mutations; a further 15% contained a novel S-gene mutation and 40% had no mutations at all in the S-gene region sequenced. Sensitivity and specificity were not calculated. The lack of finding of S-gene mutations in tissues from cats with FIP in this study highlights a possible sensitivity issue with mutation detection or that the FCoV associated with FIP in this Western Canada study had additional or alternative virulence sites that were not identified in the ‘traditional’ region of the S gene targeted by sequencing in the study.

##### Diagnostic Use of S-Gene-Mutation Analysis on Effusion and Other Fluid (e.g., CSF, Aqueous Humour) Samples

S-gene mutation analysis has also been performed on effusions in multiple studies using different methods with variable sensitivities of 40% (mutation analysis performed by sequencing) [256], 60% (mutation analysis by pyrosequencing and sequencing) [316], 65% (mutation analysis by sequencing) [312] and 69% (mutation analysis by allelic discrimination) [290]. One study [68] of samples from cats with suspected FIP found the M1058L S-gene mutation in 89 of the 94 (95%) samples in which mutation analysis was possible.

A study [106] that evaluated 51 fluid samples (primarily effusions but also included CSF and aqueous humour) from 57 cats with FIP and 47 fluid samples from 45 cats without FIP calculated that S-gene-mutation analysis (via pyrosequencing and sequencing), performed in addition to (i.e., following) the detection of FCoV alone by RT-qPCR, did not increase specificity (it stayed at 98% for both RT-qPCR for FCoV alone and for RT-qPCR for FCoV followed by S-gene-mutation analysis) for the diagnosis of FIP, but markedly decreased sensitivity from 78% (RT-qPCR alone) to 60% (RT-qPCR for FCoV followed by S-gene-mutation analysis). Another study [290] that carried out the same calculations on effusion samples, described an increase in specificity from 88% to 96% for S-gene-mutation allelic discrimination analysis over FCoV RT-PCR alone, whilst sensitivity decreased from 97% to 69%. However, only effusions from three cats without FIP were FCoV RT-PCR-positive and in only one of these was S-gene-mutation analysis successful in generating a result (and a mutated virus was detected in this one effusion). Thus, the improvement in specificity was not based on the better detection of non-mutated FCoV in non-FIP samples but by the methodology not being successful in sequencing non-FIP FCoV samples and thus not being able to detect mutated FCoV in them. Another study [301] documented several S-gene mutations (eight including M1058L and S1060A) by Sanger sequencing in the majority of effusion samples from cats with FIP, but sensitivity and specificity calculations were not reported for mutation analysis.

Another study [305] using commercially available allelic discrimination analysis, evaluating aqueous humour samples by FCoV RT-PCR with subsequent mutation analysis, concluded that mutation analysis was not helpful for the diagnosis of FIP; in this study, of 11 aqueous humour samples that were FCoV RT-PCR-positive in cats with FIP, only 4 yielded successful results for mutation analysis (3 had a mutated virus detected and 1 had mixed mutated and non-mutated viruses detected).

Similarly, researchers using the commercially available allelic discrimination assay to evaluate CSF samples by FCoV RT-PCR with subsequent mutation analysis also concluded that mutation analysis was not helpful for the diagnosis of FIP [280]. In this study, of nine CSF samples that were FCoV RT-PCR-positive in cats with FIP, only three yielded results for mutation analysis (all three were positive for the presence of S-gene mutations) [280]. In this study, the sensitivity of mutation analysis in cats with FIP was only 10% (rising to only 17% when only cats with neurological FIP were considered). The specificity of mutation analysis could not be calculated as none of the cats without FIP yielded positive FCoV RT-PCR results upon which to subsequently perform mutation analysis by allelic discrimination.

Overall, these data show the variability in results detecting S-gene mutations using the different studies and methods. This makes it very difficult to rely on S-gene-mutation analysis for the confirmation of FIP, especially when the commercially available allelic discrimination assay is used, and caution is urged in the interpretation of results. Ahead of any S-gene-mutation analysis, one should remember that, as described above in Section 7.5.2 on Detection of FCoV RNA by RT-PCRs, a positive RT-qPCR result for FCoV RNA is not specific for FIP, but high FCoV RNA viral loads do highly support a diagnosis.

#### 7.5.4. Detection of FCoV RNA by In-Situ Hybridisation (ISH)

One study has described the use of ISH to detect FCoV sequences in fixed samples prepared from enucleations of cats with FIP [245]; the technique had a similar diagnostic performance to IHC and RT-PCR. More studies are required to evaluate the use of ISH in the diagnosis of FIP.

**Summary of Section 7: Diagnosis of FIP; Section 7.5: Direct Detection of FCoV** FCoV antigen detection by immunostaining Immunostaining exploits the binding of antibodies to host-**cell-associated FCoV antigens**, which are subsequently visualised by enzymatic or immunofluorescent reactions producing a colour change in a process called **immunohistochemistry (IHC) on biopsies** or **immunocytochemistry (ICC) or immunofluorescence (IF) on cytology samples** (such as effusion and fine-needle aspirate [FNA] sample smears). The histopathological and cytological changes associated with FIP are typically **pyogranulomatous**. **Definitive diagnosis of FIP** relies on consistent **histopathological changes in affected tissues in addition to FCoV antigen immunostaining by IHC**. Consistent **cytological changes** in affected tissues in addition to **FCoV antigen immunostaining by ICC or IF** is also **highly supportive of a diagnosis**. Although positive FCoV antigen immunostaining can usually be used to confirm the diagnosis, a **negative result does not exclude FIP** as FCoV antigens can be variably distributed within lesions and might not be detected in all samples prepared from FIP-affected tissues or samples (e.g., if an effusion is cell-poor and/or the FCoV antigen is masked by FCoV antibodies in the effusion). It is important for clinicians to be aware of **variations in immunostaining techniques** and to be familiar with the specificity of the methodology employed by their local laboratory, as well as confirmation of the inclusion of negative controls in testing, when interpreting positive results. Differential diagnoses for pyogranulomatous inflammation include other infections (mycobacteria, toxoplasmosis, actinomyces, nocardia, rhodococcus, bartonella, pseudomonas and fungi) as well as idiopathic sterile pyogranulomatous disease. The **sample sites most likely to be useful are those that are affected by the FIP disease**, and inference of this can be gained from the clinical signs as well as results of diagnostic testing (e.g., ascites, neurological signs, imaging results, pyogranulomatous inflammation on FNA cytology). **Biopsy samples of affected tissues** (e.g., liver, kidney, spleen, mesenteric lymph nodes) can be collected by laparotomy, laparoscopy or ultrasound-guided tru-cut for **histopathology and immunostaining,** whereas **effusions, FNAs** (e.g., of mesenteric lymph nodes), cerebrospinal fluid (**CSF**) and **aqueous humour samples** can be collected for **cytology and immunostaining**. It is wise to **consult the diagnostic laboratory before submitting samples for ICC or IF** as their preferences for how samples should be prepared before sending vary. FCoV RNA detection by reverse-transcriptase polymerase chain reaction (RT-PCR) **FCoV RT-PCR** assays can be used to detect FCoV RNA in **blood, effusion, tissue** (including samples obtained by **FNAs**), **CSF**, or **aqueous humour** samples. The RT-PCR assays used should be **quantitative** and report the FCoV load (amount) present in the analysed sample. The load is helpful because **the systemic FCoV infection that can occur in healthy cats and cats without FIP have lower FCoV viral loads than in cats with FIP**. Thus, a positive FCoV RT-PCR result on a sample is not totally specific for FIP, but positive results with a high FCoV load on samples from cats with signs consistent with FIP are very supportive of a diagnosis of FIP, and often this is adequate evidence upon which to start a cat with antiviral FIP treatment. However, a negative result cannot rule out a diagnosis of FIP since the levels of FCoV in samples can be too low or have too variable a distribution (and thus not present in the sample analysed) to be detectable by PCR. It is wise to **consult the diagnostic laboratory before submitting samples for RT-PCR**, as their preferences for how samples should be prepared before sending vary (e.g., centrifugation of effusions, preservation advice). Recent studies using RT-PCR on blood samples have shown more promising results than previously, with high levels of FCoV RNA detectable, suggesting that **blood samples could be revisited as a diagnostic sample to support a diagnosis of FIP**. **RT-PCR analysis of effusion samples in cats with FIP is often positive** (72–100% of samples) for FCoV RNA, and cats without FIP are usually RT-PCR-negative, and the presence of **FCoV RNA, particularly in high levels, in an effusion that also has cytological and biochemical features suggestive of FIP, is highly supportive of a diagnosis of FIP**. Whilst tissue biopsy samples obtained from affected tissues in cats with FIP usually show high levels of FCoV RNA in them, as determined by RT-PCR, such samples, if collected, should ideally be submitted for histopathology and IHC, as this allows for a definitive diagnosis of FIP. **FNAs are a good sample type for FCoV RT-PCR, with the advantage of relatively easy collection**. The sample site should be guided by where pathology is likely based on clinical signs and other diagnostic investigations, but **promising results on FNAs collected from mesenteric lymph nodes from cats with FIP that did not have effusions have been obtained**. **CSF and aqueous humour FCoV RT-PCR in cats with neurological signs or ocular signs, respectively, can also be helpful**. RT-PCR on faecal samples is only useful to identify cats shedding FCoV for the management of FCoV in multi-cat households. **Faecal RT-PCR is not useful for the diagnosis of FIP** as many healthy cats without FIP shed FCoV. Characterising FCoV spike (S)-gene mutations following positive RT-PCR for FCoV RNA Following the detection of FCoV RNA in a sample by RT-PCR, varied molecular techniques (e.g., pyrosequencing and Sanger sequencing often used in research, or methods that detect and quantify specific FCoV mutation sequences, such as the commercially available allelic discrimination assay) can be used to **derive S-gene sequence data for the FCoV present**. **Such techniques are only successful at determining the FCoV sequence present when high loads of FCoV RNA are present**, so **successful S-gene-mutation analysis at least suggests that the sample contained high levels of FCoV RNA**, which is **highly supportive of a diagnosis of FIP**. However, research has shown great variability in results when detecting S-gene mutations using the different methods, making it difficult to rely on S-gene-mutation analysis as being confirmative for FIP, especially when the commercially available allelic discrimination assay is used.

### 7.6. Indirect Detection of FCoV

#### 7.6.1. FCoV Antibody Testing

##### Antibody Testing on Blood Samples

Serum FCoV antibody tests are usually ELISAs, indirect immunofluorescence antibody (IFA) tests or rapid immunomigration tests [134]. The porcine coronavirus TGEV, or FCoV, can be used in these tests as antigen substrates, both being able to detect serum FCoV antibodies; indeed, using TGEV as a substrate in one study [75] showed higher sensitivity in the detection of serum FCoV antibodies than when using FCoV as a substrate. However, false-positives have been found to be slightly more likely in some assays based on the TGEV antigen [134].

A positive FCoV antibody test indicates that the cat has encountered FCoV (by natural infection or FCoV vaccination, although this vaccine is rarely used) and has developed antibodies; seroconversion typically occurs around 7 to 28 days following natural infection [85,131,203]. Although cats with FIP tend to have higher FCoV antibody titres than cats without FIP [94], there is much overlap, with no difference between median FCoV antibody titres in healthy and suspected FIP cases, so using the value in an individual cat to distinguish cats with FIP is very limited [323].

It has been suggested that a negative serum-FCoV antibody result in a suspected FIP case that does not have an effusion is more useful to rule out a diagnosis of FIP than in a cat with an effusion [46,134,259]. However, negative results have been reported in three of seven cats with neurological FIP without effusions [238], although in that study, the method of FCoV antibody testing was not described. It is important that the FCoV antibody assay used has adequate sensitivity; otherwise, false-negative results can occur [134]. In most tests, antibody titres are determined in multiples of serum dilutions. FCoV antibody testing that begins with a dilution of the sample of 1 in 100, or 1 in 400, is commonly insensitive, missing titres lower than the starting dilution (i.e., those less than 100 or less than 400). Only tests with a starting dilution of 1 in 25 or less are recommended.

Opinions on the usefulness of antibody testing in cats suspected to have FIP vary, but there is no ‘FIP antibody test’; all that can be measured is antibody against FCoV. It is never correct to believe that a positive FCoV antibody test in an otherwise healthy cat indicates a diagnosis of FIP.

##### Antibody Testing on Effusion Samples

FCoV antibody tests, including in-house rapid tests [324], can be performed on effusion samples; 70% of 28 ascitic samples from FIP cats were positive, compared to none of 15 samples from cats without FIP [324]. Titred testing can also be performed. In one study, some cats with FIP (although the diagnosis was not confirmed in all cases) had unexpectedly low FCoV antibody titres in their effusions [325] and an inverse correlation between FCoV RNA load, measured by RT-qPCR, and FCoV antibodies were found in some samples, suggesting that FCoV can bind antibodies, rendering them unavailable as a ligand in the antibody test [325]. False-negative results for FCoV antibodies on effusions can be a problem particularly with rapid immunomigration/immunochromatography tests [134,325]. However, other studies [301,326] found no evidence of an inverse correlation between FCoV RNA loads and antibody titres in effusions from cats with suspected FIP. Both studies concluded that a combination of both FCoV RT-PCR and antibody testing would be more helpful to support a diagnosis of FIP compared to either test alone [301,326]. Similarly, one study described the success of combining a newly developed FCoV N-gene IFA test with FCoV RNA RT-PCR testing on effusions for the diagnosis of FIP [69].

##### Antibody Testing on CSF Samples

FCoV antibody testing has been performed on CSF samples in cats with FIP with varied results. One study [35] reported it to be useful in diagnosing FIP, with the comparison of serum and CSF FCoV titres suggesting intrathecal FCoV antibody production was occurring, although no controls were included in this study. Another study [282] found a significant correlation between serum and CSF FCoV antibody titres, suggesting that any CSF FCoV antibodies detected were derived from blood, and thus their detection was not additionally useful for the diagnosis of FIP. Soma et al. [319] suggested that a CSF FCoV antibody titre of greater than 640 might be useful for the diagnosis of FIP, although the diagnosis of FIP was not histopathologically confirmed in the cats in this study. Thus, it may well be that a combination of both FCoV RT-PCR and antibody testing would be most helpful to support a diagnosis of FIP compared to either test alone, although the small volumes of CSF obtained from cats for diagnostic purposes may preclude antibody analysis.

**Summary on Section 7: Diagnosis of FIP; Section 7.6: Indirect Detection of FCoV:** ** Serum FCoV antibody tests**, performed on blood, are usually enzyme-linked immunosorbent assays (**ELISA**), **indirect immunofluorescence antibody tests** or **rapid immunomigration tests**. A **positive FCoV antibody test indicates that the cat has been infected with FCoV and has developed antibodies**. Although cats with FIP tend to have higher FCoV antibody titres than cats without FIP, there is much overlap, **so there is little value in an individual cat undergoing serum FCoV antibody testing**. In addition, negative serum-FCoV antibody results cannot rule out FIP, as cats with confirmed FIP can be FCoV antibody-negative. There is no ‘FIP antibody test’; all that can be measured is antibody against FCoV.

## 8. Epidemiological Considerations in the Management of Cats following a Diagnosis of FIP

### 8.1. Does a Cat with FIP Pose a Threat to Other Cats in Its Household?

Often the question arises whether it is dangerous to bring a cat with FIP back into a household with other cats. The short answer is no, it is not. In-contact cats have been likely exposed to the same FCoV isolate that originally infected the cat that has FIP. Still, the key question remains whether mutated virus associated with the switch from enteric infection to systemic infection and the development of FIP could be transmitted from cat to cat. To answer this question, some facts concerning FCoV epidemiology need to be considered.

According to different studies (Table 1), 33 to 100% of cats with FIP shed FCoV in their faeces [94,106,107,112,166]. One study by Chang et al. in 2010 [112] on cats from the same household, which had either no clinical signs of FCoV infection or had FIP, revealed that 11 of 17 cats with FIP had no detectable intestinal FCoV and had seemingly cleared their primary FCoV infection [112]. In those cats with FIP that did have detectable intestinal FCoV, sequence analysis focusing on the FCoV 3c gene revealed that in all but one cat, the virus was different to the FCoV associated with FIP lesions, and thus seemed to have been acquired by FCoV superinfection from other cats in the household, resulting in renewed FCoV shedding [112]. The authors concluded that if a cat with FIP restarts shedding, this is likely due to a new FCoV superinfection and not the original FCoV that resulted in FIP [112]. Suggestion of a similar FCoV intestinal superinfection and repeat shedding has been reported in cats successfully treated with oral GS-441524 [94,219], with one study sequencing the faecal FCoV in the treated cats [94]. This study [94] also evaluated faecal samples from companion cats that lived with the cats with FIP and found that all of the sequencing results derived from faecal samples showed an absence of the S-gene mutations, in contrast to those FCoV found in the effusion and blood samples that were successfully sequenced from the cats with FIP. In the original study by Chang et al. in 2010 [112], the one cat with FIP that was shedding a FCoV strain in its faeces similar to the FCoV strain found in its ascitic fluid was believed to have been doing so due to leakage of systemic virus into the intestines due to, for example, an intestinal granuloma.

In another study, it was reported that faecal FCoV from cats with FIP can carry the same S-gene mutations as FCoV found systemically [106], and one study found that the full genomic RNA sequences of field FCoV strains isolated at post-mortem examination from the jejunum and the liver of a cat with FIP revealed 100% nucleotide identity between the enteric (jejunum)- and non-enteric (liver)-derived viral RNA sequences, suggesting that FIP-associated FCoV can be shed under some circumstances [327]. However, even if FIP-associated FCoV is shed in the faeces of cats with FIP, it likely cannot cause FIP following transmission to another cat, as one study demonstrated that the faeces of cats with FIP did not cause FIP in another cat [99].

The current understanding is that the horizontal transmission of FIP, via an FIP-associated FCoV strain, is a very unlikely occurrence, although FIP outbreaks are occasionally reported (see Section 4 on Pathogenesis). As mentioned earlier (Section 3.1 on Transmission of FCoV), the possibility of mechanical vectors being involved in the transmission of a highly virulent strain of FCoV has been suggested during the early investigation of a large outbreak of FIP in Cyprus [98].

Therefore, as previously stated, it is likely safe to take a cat with FIP back into a household with cats that have already been in-contact with it, as these cats are likely to be already FCoV-infected. It is, however, not recommended that the cat with FIP has contact with any ‘naïve’ FCoV-uninfected cat, because if the cat with FIP is shedding FCoV, it could infect any naïve cats with FCoV. However, of course, such FCoV infection in the naïve cat will not usually result in the development of FIP.

In households where a cat with FIP has been euthanised, with no remaining cats in the household, it is recommended that the owner waits for two months before obtaining new cats, because FCoV might preserve its infectivity for days to a few weeks, depending on environmental conditions [53], such as in desiccated faeces. Thorough vacuuming and steam cleaning can also diminish environmental FCoV load considerably; this will reduce the chance of any new cats, if they are not already FCoV-infected, becoming infected with FCoV when introduced into the household. However, as highlighted in Table 1, the prevalence of FCoV-infection is high amongst many groups of cats, so many cats are already FCoV-infected before rehoming.

### 8.2. Management of Cats with FIP in the Veterinary Practice

Cats with FIP in a veterinary practice or hospital should be handled and housed like other cats, with routine infection-control measures, as any hospitalised cat is a potential source of FCoV infection [328]. There is no benefit in isolating the cat with FIP and it is not necessary to keep cats with FIP in isolation wards. Routine infection-control measures also help protect these cats from secondary infections, as their immunity is likely to be suppressed and many are lymphopenic [18,43].

**Summary on Section 8: Epidemiological Considerations in the Management of Cats Following a Diagnosis of FIP** It is likely **safe to take a cat** that has been diagnosed **with FIP back into a household with cats that have already been in contact with it**, as these cats are likely to be already FCoV-infected following **exposure to the same FCoV isolate that originally infected the FIP cat**. In the cat that has developed FIP, the infecting FCoV has likely undergone mutations to result in FIP-associated FCoV infection, and the understanding is that the **horizontal transmission of FIP, via an FIP-associated FCoV strain, is a very unlikely occurrence.** In households where a cat with FIP has been euthanised, with no remaining cats in the household, it is recommended that the **owner waits for two months before obtaining new cats**, as it has been suggested that FCoV might preserve its infectivity for days to a few weeks. Cats with FIP in a veterinary practice should be handled and housed like other cats, with routine infection-control measures, as any cat is a potential source of FCoV infection. There is **no need to keep cats with FIP in infectious disease isolation wards**.

## 9. General Prognosis for FIP

Before effective antiviral treatments became available (Section 10 on Treatment of FIP), occasionally, cats with FIP did survive for several months or years after a diagnosis was made [37,43,224,250,329,330]. These reports described that cats had received variable treatments (e.g., non-steroidal anti-inflammatory drugs [NSAIDs], polyprenyl immunostimulant, recombinant feline IFN-omega and/or glucocorticoids), although the influence of these treatments on survival was not proven. Historically, cats with FIP usually died or were euthanised within a few weeks of presentation [27].

In a prospective study of 43 cats with FIP with effusions given systemic (2 mg/kg/day) or intracavitatory glucocorticoids, the median survival time after definitive diagnosis was only eight days [37]. Another study of cats with FIP reported a median survival time of 21 days after presentation in cats with effusions and 38 days in cats without effusions [27]. The disease progression between the onset of clinical signs and death is variable, but it appears to be shorter in younger cats and cats with effusions than in older cats and cats without effusion [9]. In two studies [27,37], high bilirubin concentration, poor general condition, low platelet count, low lymphocyte count, low haematocrit, low sodium concentration, low potassium concentration, high AST activity, and a large volume of effusion indicated a poor prognosis. Seizures can also be considered a poor prognostic sign, since they occur significantly more frequently in animals with a marked extension of inflammatory lesions in the forebrain [240]. More recently, the use of prednisolone (as well as other glucocorticoid treatments) has been suggested by some to be associated with a poorer outcome of FIP when used with other treatments [43]; in this study, prednisolone had been given to only 44% (11/25) of cats that recovered from FIP (varied other treatments were given such as recombinant feline IFN-omega and nucleoside analogues [see later]), whereas it had been given to 92% (11/12) of cats that ultimately did not recover. In a study based on owner-reported survey data describing 393 cats treated with unlicensed, mainly injectable GS-441524, high success rates were reported (88% of owners reported an improvement in clinical signs within a week of treatment and 97% of cats were still alive at the time of the survey) despite steroids being used in 38% of cats [23]. However, the reliability of these data is less compared to those derived from veterinary records, and there may have been a bias to participation by owners whose cats had undergone successful treatment. Further studies on the effect of glucocorticoids on the recovery of FIP in the presence of antivirals are required.

**Summary on Section 9: General Prognosis for FIP** Before effective antiviral treatments became available, cats with FIP usually died or were euthanised within a few weeks. **Occasionally, cats with FIP did survive for several months or years after diagnosis, with variable treatments,** although the influence of treatment on survival was not proven. Disease progression seems to be quicker in younger cats and cats with effusions than in older cats and cats without effusions.

## 10. Treatment of FIP

The recent availability of predictably effective antiviral treatments (Table 2), notably the nucleoside analogue GS-441524 [19,23,24,31,38,39,40,43,174,219,331], for FIP has totally changed the landscape of this disease for both owners and veterinary teams. There is now an alternative to euthanasia, and FIP is frequently curable. The new antiviral treatments, which act quickly, also allow for the trial treatment of cats—for example, those in which FIP is very likely (Figure 11, Figure 12 and Figure 13)—without absolute confirmation of diagnosis, due to the clinical improvement seen within a few days of treatment. A rapid and sustained positive response to antiviral treatment is a means of supporting a diagnosis of FIP. However, treatment is often expensive, not licensed and not available legally in many countries, which complicates access to care. Despite this, much published evidence exists for the efficacy of antivirals for FIP, as described in the Section 10.1 on Antiviral Treatments for FIP.

As antivirals are often expensive, care should be taken in discussions with owners regarding the costs of treatment and monitoring, as well as diagnostics, so that the cat’s care plan can be adapted to preserve funding for treatment if needed. However, costs will still preclude treatment in some cats, so veterinary teams and owners must understand that sometimes euthanasia is the only, and appropriate, option for an ill cat in that situation. No other treatments are as effective as the antivirals for FIP, although mefloquine, IFN and/or the NSAID meloxicam have been used palliatively.

### 10.1. Antiviral Treatments for FIP

Table 2 outlines antiviral agents that have been used for the treatment of FIP.

#### 10.1.1. GS-441524, a Nucleoside Analogue

The introduction of the adenosine nucleoside analogue, GS-441524, the active component of remdesivir, has revolutionised FIP treatment [38,331].

Nucleoside analogues act as an alternative substrate for viral RNA synthesis, resulting in RNA chain termination during viral RNA transcription via the inhibition of the viral RNA-dependent RNA polymerase. Although licensed preparations of GS-441524 do not currently exist for animals nor humans, veterinarians in some countries (including the UK [45,332] and Australia [17], and some other countries legally allowing their importation) have access to veterinary compounded ‘special’ formulations of GS-441524, which can be used legally, as no other licensed products exist. In the UK and Australia, where these formulations are manufactured, they are closely regulated, quality-assured (content and purity) and of known stability. Evolving protocols have emerged for the use of these compounded products in the treatment of FIP [17,45]. In other countries, only illegal preparations are available, which many owners obtain themselves to treat their cats [23]. Veterinarians whose clients are using illegal preparations might need to contact their professional regulatory bodies for guidance on their legal position in dealing with such cases, as prescribing or administering such treatments may be forbidden [333,334,335]. However, the provision of veterinary-led supportive care to cats undergoing treatment (see Section 10.3 on Supportive Treatments for FIP, including Anti-Inflammatories and Drainage) and their owners in these circumstances is highly recommended [43], and this was believed to be an important component of the excellent responses to therapy with oral GS-441524 in a prospective study [24].

**Table 2:** Antiviral drugs that have been suggested for use in cats with FIP. PO indicates orally, SC indicates subcutaneously, IV indicates intravenously, ALT indicates alanine aminotransferase. Note: For all antiviral treatments, it is important to ensure the dose given to the cat preserves appropriate dosage if/when weight gain occurs as a result of recovery, e.g., in growing kittens and adults that have had weight loss. If the dose is not adjusted, underdosage occurs, which may be associated with disease relapse [17]. Accurate weight recording is also important to monitor response to treatment [38].

Drug	Comments	ABCD Recommendation in FIP
GS-441524	Nucleoside analogue that terminates the RNA chain of viral RNA-dependent RNA polymerase. Given PO or by SC injection. Injections SC often sting. Very promising results in vitro, in one in vivo experimental study [331], and in several in vivo field studies, although FIP was not confirmed in all cases [20,38,40,43]. Survival rates of 81% mainly in cats with effusions [38], 82% in cats with effusions [19], 85% in cats with ‘mixed’ effusive/non-effusive FIP [31] and 94% in cats with FIP without effusions [31] have been reported. An improvement rate of 88% (many cats were still on treatment at time of writing) [23] has also been reported. More recent reports are using GS-441524 PO, which facilitates compliance. A prospective study showed 100% efficacy in 18 cats with FIP treated with PO GS-441524 [24]. Often expensive. Most studies have used 84-day treatment courses [19,31,332] but shorter courses may be effective [20,39,43]. Non-clinically significant transient adverse effects include elevations in ALT (although this may not be an adverse effect of GS-441524 [17] but due to the FIP), lymphocytosis and eosinophilia. Reports of GS-441524 urolithiasis are emerging but published reports are required (Séverine Tasker, personal communication). Optimal frequency of PO administration (i.e., q 24 h or q 24 h) has not been confirmed, although the doses needed for higher dosages (e.g., for neurological disease) are usually given divided q 12 h.	No licensed product available. Available as a compounded ‘special’ formulation for veterinary use in UK and Australia (and some other countries allowing importation). Owners often obtain illegal preparations in countries in which legal sources are not available. Excellent curative results. Standard dosages of 10–12 mg/kg q 24 h have been used. Higher dosages used for cats with ocular (15 mg/kg q 24 h) or neurological (10 mg/kg q 12 h) signs, but controlled studies are lacking. Many give 84 days of treatment [19,31,330], and treatment length can be extended and/or dosage increased (by 5 mg/kg/day) if clinical signs and serum biochemistry do not normalise [17]; some have suggested treating for 14 days beyond normalisation [17]. Courses shorter than 84 days may be effective [20,39,43]. PO route usually favoured (round up to nearest half 50 mg tablet if possible) due to painful SQ injections. PO tablets usually given on an empty stomach, at least 30 min before food. However, should the cat vomit the pills, then giving food can prevent vomiting (Diane Addie, personal communication).
Molnupiravir (EIDD 2801)	Nucleoside analogue given PO; promising results as a first-line and rescue (following GS-441524 treatment in cases that relapse) treatment for FIP with few adverse effects (folded ears [which may be due to the disease rather than an adverse effect], broken whiskers and severe leucopenia at very high dosages) [44].	Licensed preparation available for use in humans in some countries. Designated as an antimicrobial reserved for human use only in European Union in 2023 [336]. Use in cats shows excellent promise. Suggested dosage is 12–15 mg/kg q 12 h for 84 days.
Remdesivir (GC-5734)	Nucleoside analogue and prodrug of GS-441524. Given SC or IV. No controlled studies on efficacy yet, but current descriptive studies [17,41,45,332,337] suggest favourable results. Induction remdesivir treatment (SC or IV) followed by maintenance SC remdesivir or PO GS-441524 was associated with 86% survival at six months in 28 cats with effusive or non-effusive FIP [17]. A combination of remdesivir (IV and/or SC) subsequently treated with oral GS-441524 (24 cats), or without GS-441524 (six cats), was associated with survival of 96%, and 33%, respectively of the 30 treated cats with effusive or non-effusive FIP [45]; 84-day treatment protocols were used. Non-clinically significant transient adverse effects may include elevations in ALT [45] but SC injections are often very painful. Expensive.	Licensed preparation available for use in humans in some countries. Available as a compounded ‘special’ formulation for veterinary use in UK and Australia (and some other countries allowing importation). Compliance problematic due to painful SC injections. More field and comparative studies required. Dosages of 6–20 mg/kg q 24 h SC or IV reported (induction 10–15 mg/kg, maintenance 8–15 mg/kg, with the higher dosages for ocular or neurological FIP [17,45]) with 20 mg/kg q 24 h of remdesivir administered as 10 mg/kg q 12 h. PO GS-441524 usually favoured over injectable remdesivir unless remdesivir is the only antiviral available and/or the cat is unable to tolerate oral medication.
GC376	Inhibits 3C-like protease. Promising results in vitro and in one in vivo experimental study, especially in cats with effusions [136]. Six of twenty cats [338] and one of one cat [43] survived in field studies, although FIP was not confirmed in all cases.	Not commercially available yet but hopefully will be available as a licensed product for treatment of FIP in the future as one company has advised it is pursuing licensing. Further controlled field studies required.
Recombinant feline IFN-omega (rfIFN-ω)	Inhibits FCoV replication in vitro and reduced FCoV shedding in 9/11 cats without FIP in a shelter [339]. In one uncontrolled study, 4/12 treated cats survived over 2 years and another 4/12 experienced remission, but FIP was not confirmed in all cases [329]. However, rfIFN-ω was not effective in one placebo-controlled study; here the cats with FIP with effusion were concurrently given high dose glucocorticoids [37], which may have impacted results. In an uncontrolled study, rfIFN-ω was associated with a positive response in seven cats in which glucocorticoids were either not used (two cats), or tapered within a few weeks (five cats) [43]. Has been used following antiviral therapy for FIP to maintain remission [39,43] but controlled studies are needed to confirm efficacy of, and need for, rfIFN-ω as many studies have shown excellent survival following nucleoside analogue (including GS-441524) treatment without follow-up rfIFN-ω to prevent FIP relapse [17,19,24,31,38,40,45].	Licensed for cats in some countries. Further studies without concurrent glucocorticoid treatment needed. Studies required to evaluate if useful to maintain remission of FIP after other antiviral treatment. Dosages in Section 10.1.6. on Interferons.
Mefloquine	Effectively inhibits FCoV replication in vitro as a small molecule inhibitor [340] and acts as a nucleoside analogue [341], but full mechanism of antiviral effects not known. Hepatic metabolism studied in vitro [342] and pharmacokinetics studied in healthy cats [343]. Its plasma protein binding properties have been studied in the blood of cats with and without FIP, and a simple high performance liquid chromatography assay developed to measure mefloquine [344]. Causes vomiting if not given with food but generally appears safe in healthy cats. Used in Australia as adjunct treatment for FIP and/or to maintain remission (Richard Malik, Sally Coggins and Jacqueline Norris, personal communication) but no published studies yet. Affordable.	Licensed preparation available for use in humans. Field studies required both alone and in combination with other drugs/antivirals for FIP treatment and to evaluate if useful to maintain remission. Has been used where other more effective antivirals cannot be used due to cost or availability. Suggested dosing is 62.5 mg/cat PO 2–3 times a week (three times for large cat) or 20–25 mg/cat PO q 24 h, with food.
Cyclosporine A and non-immunosuppressive derivatives (e.g., alosporivir)	Inhibits cyclophilins and thereby blocks replication of FCoV in vitro [345,346]. Associated with a reduction in blood FCoV viral load in three cats with suspected FIP, and reduced pleural effusion FCoV viral load and volume in one cat that survived 264 days after presentation before dying (see supplementary data in Tanaka et al. 2015 [347]). Can lead to immunosuppression, depending on the cyclosporine A derivative.	Further field studies needed.
Curcumin	Curcumin-encapsulated chitosan nanoparticles decreased expression of pro-inflammatory cytokines during infection of cell cultures with FIP-associated FCoV and inhibited viral replication in vitro [348]. Enhanced bioavailability as curcumin-encapsulated chitosan nanoparticles over curcumin in pharmacokinetic analysis in healthy cats [348]. Not effective as a small molecule inhibitor of FCoV replication in vitro [340]. Anti-inflammatory properties.	Further studies needed.
Chloroquine	Inhibits endocytosis following attachment of FCoV to host-cell membrane [341]. Has anti-inflammatory effects in vivo [349]. Can increase liver enzyme activities. Effective as a small molecule inhibitor of FCoV replication in vitro [340] but reported as being too toxic for cats [340,349]	Not recommended.
Hydroxychloroquine	Inhibits endocytosis following attachment of FCoV to host-cell membrane [341]. Inhibits type I and II FCoV replication in vitro, with less evidence of cytotoxicity than chloroquine [350]; addition of rfIFN-ω increased its antiviral action against type I FCoV replication in vitro.	Not recommended until further studies available.
Itraconazole	Inhibits cholesterol transport in type I FCoV in vitro [351] and thus, inhibits FCoV replication. Also said to inhibit endocytosis following attachment of FCoV to host-cell membrane [341]. Synergism of itraconazole with GS-441524 shown with type I FCoV in vitro [352]. Used in a very small uncontrolled study of cats with experimentally induced FIP alongside anti-human-TNF-α antibody treatment [190], in which two of three cats with FIP improved, and in one field case alongside prednisolone where the cat initially improved but relapsed and was euthanised at 38 days [353]. Reduced faecal virus load but failed to eliminate FCoV infection [132]. Associated with anorexia and vomiting in cats without FIP [354].	Not recommended. More effective treatments now available.
Nelfinavir	Acts as protease inhibitor that showed synergistic effects against FCoV in vitro with Galanthus nivalis agglutinin [355]. No in vivo data available.	Not recommended until further studies available.
Ribavirin	Acts as a nucleoside analogue [341]. Inhibits FCoV replication in vitro, but very toxic in cats [356,357,358].	Not recommended.
Vidarabine	Inhibits polymerases and reduces FCoV replication in vitro, but in vivo efficacy unknown [359]. Toxic to cats if given systemically.	Not recommended.
Galanthus nivalis agglutinin	Binds to FCoV-glycosylated envelope glycoproteins, thereby inhibiting viral attachment to the host cell and showed synergistic effects against FCoV with nelfinavir in vitro [355]. No in vivo data available.	Not recommended until further studies available.
Indomethacin	Acts as cyclopentenone cyclooxygenase metabolite with activity against several RNA viruses, including canine coronavirus [360]. No data on efficacy against FCoV in vitro or in cats with FIP available. Safety in cats is unknown.	Not recommended until further studies available.

##### Initial Studies on GS-441524

The first published studies on the efficacy of GS-441524 for the treatment of FIP used an injectable preparation of GS-441524 and tended to administer it for shorter periods and at lower dosages than are used now. These studies initially excluded cats with neurological or ocular signs [38,331].

GS-441524 was shown to be non-toxic in vitro and effectively inhibited the replication of FIP-associated FCoV strains and field FCoV isolates in two different cell culture systems [331]. In 10 young cats with experimentally induced FIP, GS-441524 (applied subcutaneously [SC] q 24 h at either 2 mg/kg or 5 mg/kg) caused a rapid reversal of clinical signs and return to a clinically healthy status within two weeks of treatment in all 10 cats. Two of the ten treated cats had recurrences of clinical signs (the nature of which was not described) at four weeks and six weeks post-treatment [331]—one from each dosage group. These two cats responded to a second treatment of two weeks of GS-441524. All 10 cats remained clinically healthy until the time of publication more than eight months post-infection [331]. No adverse effects were noted other than a transient ‘stinging’ injection reaction in some cats (e.g., unusual posturing, licking at the injection site, vocalisations) directly after administration [331].

GS-441524 treatment was then evaluated in a field clinical trial of 31 cats with naturally occurring FIP [38]. Cats were diagnosed with FIP based on signalment, history, clinical examination, prior test results, repeat testing and/or effusion analysis, with FCoV RT-PCR performed on effusions in eight cats. A more definitive diagnosis of FIP was desirable but not essential, and tissue IHC for FCoV antigens was performed in only five cats that died or were euthanised and underwent post-mortem examination. Cats with neurological or ocular signs were discouraged from the trial due to concerns of poor penetration of GS-441524 into the brain and/or eye from previous studies [331] (although subsequent studies have described the effective treatment of neurological or ocular FIP with higher dosages of GS-441524 [24,39,40,45], see below).

Of the 31 cats with FIP recruited into the field clinical trial [38], 5 had no evidence of effusion. Cats had a mean age of 14 months (range 3–73 months). The cats were started with a first treatment course of GS-441524 at a dosage of 2 mg/kg SC q 24 h, usually for at least 84 days (i.e., 12 weeks, with longer treatment given if serum protein levels remained elevated). The dosage was increased to 4 mg/kg SC q 24 h for subsequent treatments in the trial when cats relapsed or when a treatment course of longer than 84 days was deemed necessary.

The study [38] did not include an untreated control group due to ethical concerns. Five of the thirty-one cats died, or were euthanised, within 26 days of the first treatment. The remaining 26 cats completed 84 days or more of GS-441524 treatment and showed rapid clinical improvement within 14 days. Of these 26 cats, 18 remained healthy, while 8 had FIP relapses (these were non-neurological in nature in 6 cats and neurological in 2 cats) at a mean (range) of 23 (3–84) days after treatment stopped. Four of the eight cats with relapses were treated again with GS-441524 at 2 mg/kg SC q 24 h; one of these four cats relapsed with neurological FIP two weeks into the second treatment and was euthanised, whilst two cats responded but then relapsed and were treated with GS-441524 at a higher dosage of 4 mg/kg SC q 24 h. The remaining cat was changed from 2 mg/kg to 4 mg/kg SC q 24 h of GS-441524 as treatment was extended due to a lack of complete response. The remaining four of the eight cats with relapses were given the higher dosage of 4 mg/kg SC q 24 h immediately, and all responded. Of the original 31 cats in the field clinical trial [38], 25 (81%) were classified as long-term survivors after successful treatment; 1 of these cats was subsequently euthanised due to presumed unrelated heart disease, while the other 24 remained healthy at the time of publication [38], confirming the efficacy of SC GS-441524. Only one cat in this study was thought to have shown evidence of drug resistance following SC GS-441524 treatment, although this was not confirmed [38].

Subsequently, a small case series describing GS-441524 treatment at higher dosages of 5–10 mg/kg SC q 24 h for at least 84 days in four cats with neurological and ocular signs of FIP was published [40]. Three of the four cats were alive and off treatment at the time of publication, 354–528 days after treatment had started; two cats had received 5 mg/kg SC q 24 h and one cat an escalating dosage of 10 mg/kg SC q 24 h. This provided evidence that FIP associated with neurological and ocular signs could also be successfully treated, albeit at higher dosages of GS-441524. The remaining cat was euthanised 216 days after starting treatment; this cat had not shown complete remission (at 5 mg/kg SC q 24 h), and rapid clinical deterioration occurred when treatment was stopped. Additionally, local skin reactions and discomfort around SC injections were cited as a reason for euthanasia.

##### Subsequent Studies on GS-441524

The problems encountered when injecting GS-441524 SC meant that formulations that could be administered per os (PO) were attractive. The first study documenting the PO treatment of a cat with FIP was a case report published in 2020 [39]. The cat had ocular signs of FIP in the absence of effusions and was successfully treated with an unregulated PO preparation of a nucleoside analogue; the preparation used was a brand for which the manufacturers did not stipulate the identity of the active agent in the preparation, although it was subsequently confirmed to be GS-441524 by independent analysis of the formulation in another study [24].

In this case report [39], the GS-441524 preparation was administered PO for 50 days at a higher dosage (believed to be 8 mg/kg/day), based on the manufacturer’s recommendation that a higher dosage was required to penetrate the eye and brain. However, the actual dosage and dose given to the cat was probably a lot more than believed, as subsequent independent analysis of the amount of GS-441524 present in the unregulated preparation used showed it to contain twice to three times more GS-441524 than stated by the manufacturer [132,174]. This discrepancy makes it very difficult to confirm the amount of GS-441524 given in studies using unregulated preparations. Nevertheless, within two weeks of starting treatment, the cat showed a marked increase in weight and improvement in ocular signs (return of vision) and multiple haematological (e.g., normalisation of haematocrit) and biochemical (e.g., marked reductions in AGP and globulin measurements and an increase in the A:G ratio) measurements. The cat was also given anti-inflammatory prednisolone (at around 2 mg/kg PO) for six days (before starting the GS-441524 preparation) followed by topical ocular steroids for uveitis treatment. Recombinant feline IFN-omega (rfIFN-ω) PO was also started after finishing 50 days of GS-441524 treatment [43]. This cat is still alive and well three years later, having received rfIFN-ω treatment for 7 months in total (Diane Addie, personal communication). No adverse effects of GS-441524 treatment were noted, although the cat did have increased symmetric dimethylarginine concentrations during treatment (but baseline pre-treatment levels were not measured), which decreased following the discontinuation of the GS-441524 preparation. The cat was also given the hepatoprotectant S-adenosyl L-methionine (SAMe) supplementation, alongside the GS-441524, as a precaution.

Since this case report was published [39], evidence has accumulated that oral GS-441524 is very effective [19,24,31,43]. Administration PO is usually less traumatic for the cat and owner, due to the often-painful nature of SC GS-441524 injections, which means compliance for the long treatment course is improved. It is not known if the route of administration of the GS-441524 influences the outcome of treatment. In a recent study of 26 cats that experienced FIP treatment relapses, 23 had been treated SC rather than PO with GS-441524 [44], but, unfortunately, no control group of treated cats without relapses was presented, so it is unknown if the reason for the relapses was related to the route of administration. Additionally, the earlier studies that used GS-441524 SC did show excellent efficacy [38,40], as was reported in a study using owner-reported data [23].

A prospective field study in 2021 described the successful treatment of 18 of 18 cats with confirmed, or highly suspected, FIP, with an oral unregulated preparation of GS-441524 [24]. It was known that GS-441524 was the main component of the unregulated preparation used, as this was confirmed by mass spectrometry. Sixteen of the eighteen cats had effusions and two did not; both these cases had ocular signs and one had concurrent neurological signs.

This study used dosages of either 5 mg/kg/day or 10 mg/kg/day, depending on the absence, or presence, of neurological and/or ocular signs, respectively, with a treatment course lasting 84 days; these dosages were those recommended by the manufacturers. It was assumed that the dose of the active component in the tablets was as described on the package inserts. However, as described earlier, subsequent analysis of the tablets used to treat the 18 cats showed them to contain more than twice the dose of GS-441524 stated by the manufacturer [174], meaning that the positive responses seen in the original study [24] were associated with dosages of GS-441524 far higher than the reported 5 mg/kg/day or 10 mg/kg/day used in the published study. Other independent analyses of the GS-441524 content of unregulated preparations have similarly shown them to contain more GS-441524 than stated by the manufacturers [132]. Some of the unregulated preparations also contain additional ingredients to the GS-441524, such as silymarin and other herbal compounds, but their effect in the treatment of FIP is not proven, especially as treatment with GS-441524 alone is known to be effective [17,38,40,45,331].

In two studies documenting treatment with oral GS-441524 [17,24], the GS-441524 was given on an empty stomach, with food 30 min later, as recommended by the manufacturers. In one of the studies, GS-441524 tablets were followed by 3–5 mL of water or a tablespoon of wet food before food 30 min later [17]. However, no studies exist confirming the need for administration without food, although this protocol has been effective.

The curative response seen in all 18 cats treated with oral GS-441524 was remarkable, with the shortest follow-up being 99 days after the completion of the 84-day treatment course [24]. All cats were hospitalised during the first eight days of treatment, and the intensive veterinary supportive care provided (e.g., intravenous [IV] fluid therapy, appetite stimulants, anti-emetics, analgesia [Section 10.3 on Supportive Treatments for FIP, including Anti-Inflammatories and Drainage]) might have contributed to the high success rate, highlighting the importance of veterinary involvement in the care of sick cats with FIP. Additionally, no serious adverse effects were seen with GS-441524 treatment; an increase in liver enzymes (11/18 cats; only 2 were given hepatoprotectants), lymphocytosis (14/18 cats) or eosinophilia (11/18 cats) were documented, in the absence of clinical signs. Although a raised ALT may be an adverse effect of GS-441524, one group found that it could resolve during treatment, or persist after treatment had stopped, making an adverse effect less likely in their opinion [17]. Additionally, eosinophilia might be a marker of successful treatment [17], rather than an adverse effect, as has been reported in human patients recovering from COVID-19 [361]. No renal adverse effects were reported [24].

Another study [94] by the same group who documented the successful treatment in 18 cats [24] used samples from the 18 treated cats to show decreasing FCoV loads in their faeces, blood and effusions during treatment with oral GS-441524. The viral RNA loads in the blood and effusions were correlated, but those in the faeces were not [94]. Levels of blood FCoV RNA fell quickly, with all cats yielding negative RT-PCR results by day 14 of treatment [94].

These 18 cats were subsequently monitored extensively, every 12 weeks, for up to one year after their GS-441524 treatment had been started [219]. Follow-up data were available for all 18 cats at 24 weeks (i.e., 12 weeks after completion of the 84-day course of GS-441524 treatment), for 15/18 cats at 36 weeks, and for 14/18 at 48 weeks. No confirmed relapses of FIP were found in any of the 18 cats, suggesting effective treatment of FIP with 84 days of oral GS-441524.

Laboratory parameters remained stable after the end of the GS-441524 treatment, as did undetectable blood FCoV loads (in all but one cat on one occasion). The recurrence of faecal FCoV shedding was detected in five cats [219].

Two cats developed mild neurological signs (neither had neurological signs at initial presentation with FIP), compatible with feline hyperaesthesia syndrome (attacks of excessive licking and twitching of the skin in the lumbar region) in weeks 36 and 48, respectively; however, FCoV-RNA remained undetectable in the blood and faeces and no increase of FCoV antibody titres was observed, suggesting that the signs were not due to FIP, although one cat did receive GS-441524 treatment sourced by the owner. The neurological signs resolved in one cat and improved markedly in the other. Delayed neurological signs could be a long-term adverse effect of the treatment or associated with a ‘long FIP syndrome,’ but further evaluation is required [219].

Interestingly, 12 of the 18 cats showed abdominal lymphadenomegaly during the follow-up period, and in 4 cats this was present constantly during treatment and the follow-up period [219]. The reason for this lymphadenomegaly is not known but could be due to an exaggerated, genetically determined, immune response associated with recovery or the presence of residual virus in the abdomen. However, one of cats with lymphadenomegaly died as a result of a road traffic accident at week 35, i.e., 161 days after finishing the 84-day course of GS-441524 [174]. This cat underwent post-mortem examination and no evidence of FIP was found on histopathology, nor was FCoV antigen nor RNA found in any tissues, demonstrating the elimination of FCoV following the successful treatment of FIP with oral GS-441524 [174], despite the lymphadenomegaly. Severe generalised follicular lymphoid hyperplasia was found on the histopathology of the abdominal lymph nodes of this cat. It is possible that the lymphadenomegaly is an adverse effect of the GS-441524. This was seen in one other FIP case treated with oral GS-441524 (Diane Addie, personal communication). A drug reaction with generalised lymphadenomegaly to phenobarbitone has been reported [362].

A very large retrospective case series documented the successful treatment of 116 of 141 (82%) pet cats with suspected FIP and effusions using an unregulated GS-441524 preparation [19] at a dosage believed to be 5 mg/kg/day (see earlier for disparity in the GS-441524 dose in this preparation compared to that stated by the manufacturers), but the frequency of administration was not stipulated; the remaining 25 cats died despite treatment. An 84-day treatment course was given, and most cats received PO rather than SC therapy; only a few cats received SC therapy, and this was said to be feasible for only a short period during early treatment and used when PO administration was not possible due to ‘disease progression’. Statistical comparison between PO and SC GS-441524 was not possible due to the small numbers treated SC [19]. Of the 116 survivors, 3 cats relapsed in the four weeks after stopping of the oral GS-441524 treatment but were said to be responding to a higher dosage of GS-441524 at the time of publication. Although the method of confirmation of diagnosis was not stated (most cats [139/141] were FCoV RT-PCR-positive on effusion samples), the study gave valuable information on which parameters might be useful to monitor to predict response to oral GS-441524 treatment. At assessment, before the start of treatment, the cats that were still alive at follow-up had had significantly better appetite and activity scores, and interestingly higher mean temperatures (39.0 °C compared to 37.9 °C), than those that had died. Additionally, survivors had had significantly lower mean bilirubin concentrations (16.1 µmol/L compared to 53.2 µmol/L); indeed, the likelihood of survival was correlated with bilirubin concentrations (Table 3).

Another large case series by the same authors [31], again evaluating 84 days of a GS-441524 preparation for suspected FIP, reported the outcome of 161 cats that were described as having ‘mixed’ FIP (i.e., with signs of both effusive and non-effusive disease)*,* and 163 cats with only non-effusive FIP (i.e., only signs of non-effusive disease). The dosages used varied depending on the ‘stage’ of FIP that the cat was allocated to, which appeared to correspond to the clinical signs of FIP present. They ranged from 7.5 mg/kg to 10 mg/kg q 24 h (although, as described earlier, there is disparity in the GS-441524 dose in these preparations). The study reported successful treatment of 137 of the 161 (85%) ‘mixed’ signs cats and 153 of the 163 (94%) cats with only non-effusive FIP [31]. Most (262/324; 81%) cats received their GS-441524 PO, with a small number of cats given GS-441524 SC at the start of treatment; only one cat was treated exclusively with SC GS-441524. GS-441524 was only administered SC when PO administration was ‘difficult owing to gastrointestinal dysfunction or FIP onset, such as an inability to absorb nutrients’ [31]. Many of the cats given SC GS-441524 died, which was said to be due to clinical deterioration, but further details were not given. Statistical comparison between PO and SC GS-441524 outcomes was not possible due to the small numbers treated SC [31], as in their previous study [19].

Similar to the last study [19], the cats in this study [31] that were alive at the completion of the 84-day treatment (termed survivors) had significantly better appetite and activity scores, and higher mean temperatures (38.9 °C in both the cats with ‘mixed’ FIP and those with non-effusive FIP) before the start of treatment than those that did not survive (37.5 °C in the cats with ‘mixed’ FIP and 37.0 °C in those with non-effusive FIP). Additionally, as before, survivors had lower bilirubin concentrations (significantly in the ‘mixed’ effusive/non-effusive FIP group and non-significantly in the non-effusive FIP group) than non-survivors [31]. Neurological signs carried a significantly poorer prognosis in this study amongst both the cats with ‘mixed’ FIP and those with non-effusive FIP [31]; combining the data in these groups revealed that 61.8% (21/34) of cats that did not survive had neurological signs, compared to only 23.4% (68/290) of cats that survived. Seizures have previously been considered a poor prognostic sign [240]. In contrast, ocular signs were not associated with survival for both the cats with ‘mixed’ FIP and those with only non-effusive FIP [31]; combining the data in these groups revealed that 11.8% (4/34) of cats that did not survive had ocular signs, compared to 12.8% (37/290) of cats that survived.

In both of the studies [19,31], body weight, haematocrit, A:G ratio and SAA levels all normalised after 84 days of GS-441524 treatment. Following the completion of the treatment, 11 cats relapsed with FIP (with mixed clinical signs such as anorexia, hypoactivity, fever, neurological signs, ascites, and pleural effusion). These cats were treated with an additional 42-day course of GS-441524 at a dosage of 10 mg/kg, but no further details of the response of these cats were given.

Another smaller retrospective study of the varied treatment of 42 cats with confirmed or suspected FIP documented the successful use of serum AGP measurements in differentiating cats that fully recovered from FIP (26 cats, at least 13 of which received GS-441524 preparations) from those did not (16 cats, none of which were given GS-441524) [43]. In this study, other varied treatments were given, but an AGP concentration of less than 0.5 mg/mL was associated with (full) recovery from FIP and was more reliable to track than the resolution of lymphopenia or hyperglobulinaemia (the hyperglobulinaemia was slower to resolve), suggesting that serum AGP concentration could be used as an indicator to stop antiviral treatment with nucleoside analogues. Further prospective studies are required to confirm this, but it is likely that AGP measurement, if available, is useful to document response to treatment. Additionally, in that study [43], some cats recovered with as few as seven to eight weeks of oral GS-441524 treatment, suggesting that shorter courses of GS-441524 may be effective and that the length of treatment could be determined by the time taken to obtain a normal AGP measurement. The study recommended that two consecutively normal AGP measurements at least a week apart were required to confirm recovery from FIP [43].

Unsurprisingly, FCoV antibody concentrations are not useful to track response to treatment. Antibody titres were found to remain elevated, even years later, in almost all cats that had recovered from FIP [43,132]. In the prospective study evaluating GS-441524 treatment [24], it was found that FCoV antibody titres did decline in 14/18 treated cats, in some cats as early as 28 days after starting treatment, whilst in others it was 56 or 84 days after starting treatment that a decline was detected. However, in the follow-up study of these cats, serum FCoV antibodies were still present in all 18 cats at the first recheck at week 24; in 14/15 at week 36; and in 13/14 at week 48. In four cats (all were free roaming or had companion cats at home), an intermediate short-term rise in FCoV antibody titres was detected, despite all cats remaining in remission for FIP [219].

A large owner-derived data retrospective study documented 393 owner (mostly in the USA) questionnaire responses on the use of unlicensed GS-441524-like treatment (mostly SC but a few with PO, or initial SC and then PO, treatment) for at least 84 days on their own cats for the treatment of FIP [23]. Of 393 owners, 88% saw an improvement in clinical signs in their cats within a week of starting the SC ‘GS-441524′ treatment. Furthermore, 54% said that their cats had been cured of FIP, whilst 43% said their cats were alive and well but still in a post-treatment monitoring period. Overall, only 13% of cats showed a relapse of FIP-associated clinical signs, whilst 3% had died despite treatment. These figures may represent an underreport of failures since owners whose cats died might have been less likely to have completed the questionnaire [23]. Varied unlicensed unstandardised preparations believed to contain GS-441524 compounds were used in the study.

The diagnosis of FIP was based on the owners’ individual veterinarian’s opinion and was not confirmed in the study, but the signalment and clinical signs reported were very suggestive of FIP, with most cats (57%) having effusions; around 43% had neurological and/or ocular signs too. Reported complications of treatment were similar to those reported previously [40,331] such as vocalisation and pain on injection [23].

Remarkably, in this study, only 9% of owners received help from their veterinarian for treatment of their cat, and most learnt about FIP treatment online [23]. It may be that the response rates could have been even higher with the aid of supportive care from veterinarians. Treatment was expensive with the average cost per cat being USD 4920 (2021 publication). Although the authors were not advocating the unauthorised use of the GS-441524-like compounds, the study is valuable for describing the experiences of owners and efficacy of such formulations. The dosages used varied greatly and were significantly higher at the end of treatment courses compared to the beginning. It is difficult to be sure of the true dosages used due to the lack of any documented quality control of GS-441524 or other compounds, nor their concentration/stability, in the preparations used.

Another retrospective study of cats with suspected FIP [20] reported that 23 of 24 (96%) cats treated with GS-441524 at 2–4 mg/kg/day for at least 28 days were cured. Unfortunately, the route of administration was not documented. Again, the true composition of the preparations administered in this study was not confirmed, but it does again suggest that shorter treatment courses may be effective.

Overall, a good appetite and/or activity level, a higher temperature, a lower bilirubin concentration [19,31] and the normalisation of AGP [43] appear to be prognostically useful to predict survival with GS-441524 treatment of FIP. Weight gain has been cited as a simple long-term measure of treatment efficacy with GS-441524 [19,31,38] and is easy to measure using paediatric weighing scales, allowing for the appropriate increased dose to be calculated to maintain the appropriate dosage. Weighing every one to two weeks is recommended.

Oral GS-441524 has also been used to eliminate FCoV shedding in cats [39], and further details on this can be found below in Section 12.3 on Elimination of FCoV Shedding.

#### 10.1.2. Remdesivir, a Nucleoside Analogue

Remdesivir, GS-5734, has been suggested as a treatment in humans for COVID-19 due to SARS-CoV-2, although clear evidence for its beneficial effect is lacking [363,364] and there have been concerns over toxicity [365,366]. In some countries, remdesivir preparations are licensed for use in humans [367], and these might be available to prescribe for use in cats. At least in the European Union [368] and the UK, veterinarians are allowed to legally prescribe remdesivir for cats with FIP if no other drugs are licensed for the treatment of FIP in cats and no other effective drugs are available to treat FIP that are licensed for treating other diseases in cats or in other animal species. However, national prescribing rules can then influence access to remdesivir.

Remdesivir is a prodrug of GS-441524 [369], yielding active GS-441524 after intracellular conversion [370,371]. Remdesivir is injected intravenously [IV] or SC as it is said to be inactive PO [372]. However, one study [373], evaluating the pharmacokinetics of remdesivir in cats, reported that oral remdesivir administration might be feasible, and oral remdesivir has apparently been used as successful treatment in cats with FIP in some countries (Sally Coggins, personal communication).

Remdesivir has been used to treat FIP in cats [17,41,45,332,337]. Descriptive case series exist of its use in Australia and the UK [17,45], where a veterinary compounded ‘special’ formulation of injectable remdesivir is available. These studies often use treatment protocols that transition from injectable remdesivir to oral GS-441524, for ease. They use injectable (IV or SC) remdesivir initially (e.g., for 1 to 14 days, sometimes with a higher induction dosage of remdesivir, see Table 2 and Table 4), usually followed by oral GS-441524 [17,45,332], or sometimes continued remdesivir (when a drop to a maintenance dosage of remdesivir [17] is instigated) to complete the 84-day treatment course.

Comparative studies between remdesivir and GS-441524 treatment are needed.

In one case series describing the treatment outcome of 32 cats with effusive (25 cats) or non-effusive (7 cats) FIP in the UK, 30 received remdesivir [45]. This comprised remdesivir alone (6 cats) or remdesivir transitioned to PO GS-441524 (24 cats). Four of the six cats given remdesivir alone died, whereas only one of the twenty-four cats given combination treatment died. However, three of the four cats that died on remdesivir alone did so within two days of starting treatment; the fourth died after 13 days of treatment. Overall, 26 of 32 cats (81.3%) in the study were alive and in clinical and biochemical remission at the end of the 84-day treatment period. In this study, a poor prognosis appeared to be associated with hypoglycaemia and the Ragdoll breed [45]. Non-clinically significant transient adverse effects can include elevations in ALT [45]. A comparison between treatment protocols was not possible due to the retrospective nature of the study and because drug availability dictated the protocol used [45].

Another retrospective case series in Australia [17] described 28 FIP cats (23 with effusions) that were treated with either remdesivir alone (15 cats) or remdesivir transitioned to PO GS-441524 (13 cats; possible once oral GS-441524 became available) for at least 84 days. Of the 28 cats, 24 (86%) survived to six months. Three cats died within two days, and, of the twenty-five cats that survived at least two days, 96% (24) survived to six months. Interestingly, 10 of these 25 cats needed an extension of the 84-day treatment and 5 cats also needed a dosage increase (5 mg/kg of remdesivir or GS-441524) when relapse of FIP was suspected (based on clinical signs and/or diagnostic testing). In three cats, a second 84-day treatment course was given [17]. The authors recorded a 30% relapse rate in the first cats treated; these had received remdesivir alone (as it was the only legally available antiviral at that time) at an induction dosage of 10 mg/kg q 24 h (as a slow IV infusion on days 0, 1, 2 and 3) followed by a maintenance dosage of 6 mg/kg (or 10 mg/kg if ocular or neurological signs present) SC q 24 h for a minimum of 84 days. Thereafter, subsequently treated cats were given higher dosages of remdesivir according to Table 4 below. Cats treated with this higher remdesivir dosage protocol, or cats given remdesivir before being transitioned to PO GS441524, did not relapse. This suggests the need for a higher dosage of remdesivir to effect a ‘cure’ and/or that a more favourable response is seen when PO GS-441524 is used in the FIP treatment protocol.

This case series from Australia [17] also provided information on what might be useful markers to monitor for treatment response. The resolution of pyrexia and inappetence was achieved within 1 week, and the resolution of icterus, effusions, and ophthalmic changes within 2 to 4 weeks of starting treatment. Monitoring body weight (every two weeks in this study) was important to assess response to treatment and ensure an appropriate dose is given, despite weight gain. Thus, regular weighing is recommended. Hyperbilirubinaemia, hyperproteinaemia and leucocyte abnormalities normalised within 2–3 weeks, but hyperglobulinaemia took around 5 weeks to normalise; the authors suggested that if hyperglobulinaemia persists beyond 42 days of treatment, a dosage increase in antiviral should be considered. Plasma albumin concentration and PCV took longer to improve and remained slightly below their reference intervals at treatment completion in some cats. The authors also noted that in some effusive FIP cases, a pattern of a drop in body weight, a serum globulin concentration spike and PCV drop occurred when an effusion was resorbed a couple of weeks into treatment; this is an important observation for veterinarians to be aware of, as it is not indicative of treatment failure, and cats usually respond well to ongoing treatment. The pattern is believed to be due to systemic protein resorption from the effusion and transient haemodilution due to body cavity fluid shifts [17].

Remdesivir IV (administered slowly over 30–60 min with a syringe driver) has been mostly used at the start of 84-day treatment courses for very sick cats that are unable to tolerate oral medication, such as those that are obtunded [17,45] or have severe malabsorption, when there are concerns over hydration status or SC injections are not tolerated, and when oral GS-441524 formulations are not available.

Remdesivir SC injections often cause local skin reactions and pain (as is also the case for injectable GS-441524), although one published case report described successful treatment of confirmed FIP with IV remdesivir for three days, followed by SC remdesivir for another 77 days, without problems [41]. This cat remained in remission at the 7-month follow-up timepoint [41]. However, in another study [17], transition from parenteral remdesivir to PO GS-441524 occurred as a result of injection site discomfort; the discomfort was described as severe in 2, and mild in 13, of the 25 cats in the study that survived 84 days of treatment. A similar need to transition from injectable remdesivir to PO GS-441524 due to painful injections was reported in another case series [45]. If SC remdesivir injections are problematic and a switch to oral GS-441524 is not possible, pre-injection oral gabapentin, rotating injection sites, room-temperature remdesivir, the use of topical local anaesthesia, oral transmucosal buprenorphine and positive behavioural engagement at the time of injection, can help tolerance [17,45,332].

Problems with injecting and cost (of injecting as well as the drug itself, as remdesivir is often more expensive than oral GS-441524) were the main reasons for switching from injectable remdesivir to oral GS-441524 in one study [45]. Indeed, many cats are treated successfully with only oral GS-441524.

#### 10.1.3. Molnupiravir, a Nucleoside Analogue

Molnupiravir, also known as EIDD-2801, has been used in humans for the treatment of COVID-19 [374,375]. In vitro studies with serotype II FCoV showed it to have promising antiviral action [376]. Its active metabolite is EIDD-1931 [44].

The use of unlicensed formulations of molnupiravir was described using data from questionnaires completed by owners whose cats with FIP had received molnupiravir treatment [44]. The molnupiravir was given either as first-line treatment in 4 cats or as rescue treatment in 26 cats that had received an initial treatment for suspected FIP with unlicensed GS-441524, or a drug combination including unlicensed GS-441524, as the main drug. Thirteen of the cats were treated with injectable GS-441524 only, three cats were treated with oral GS-441524 only, and an additional seven were treated with a combination of injectable and oral GS-441524 throughout the duration of therapy. Two were treated with a combination of unlicensed GS-441524 and unlicensed protease inhibitor antiviral GC376 (see Section 10.1.4 on GC376, a protease inhibitor), whilst one cat was treated with injectable and oral GS-441524, injectable GC376 and molnupiravir. Sixteen cats had received one initial treatment course based on GS-441524 before receiving molnupiravir, seven had received two courses, and three cats had received three courses [44]. The reported apparent (as the dose content of the preparations is not known) starting dosages for the unlicensed GS-441524 used in the cats ranged from 2 mg/kg to 10 mg/kg; with the most common dosages being 5–6 mg/kg (eight cats) and 10 mg/kg (seven cats). Most (21) cats were dosed q 24 h, four were dosed q 12 h, and one cat was initially dosed q 12 h for one week before switching to q 24 h. The median duration of GS-441524-based therapy was 84 days before starting molnupiravir. Thus, the initial treatment course dosages were very varied.

The reason for the apparent failure of GS-441524 treatment was unknown but could have been due to inappropriate dosages, as unlicensed preparations of GS-441524 were used, or due to the emergence of resistance to GS-441524. A dosage range of 12–15 mg/kg PO q 12 h of molnupiravir for 12–13 weeks was reported as being successful, with the higher end of the dosage range used to treat cats with neurological signs. Of the 30 cats in the report, 28 (including 24 of the 26 cats that received molnupiravir as rescue treatment) were living disease-free at the time of publication. Very few adverse effects were reported, but included, rarely, and at very high dosages of 23 mg/kg PO q 12 h, broken whiskers and severe leucopenia [44]. A previous pharmacokinetic study of 10 mg/kg of oral molnupiravir in cats had reported nausea post-administration [373], but this was not reported in the retrospective study.

No licensed products of molnupiravir are available for use in cats, but a human-licensed product exists for the treatment of COVID-19, with low rates of resistance to SARS-CoV-2 reported in in vitro studies [375]. Although the human-licensed products cannot be used in cats legally in all countries (e.g., molnupiravir is restricted to human use only in the European Union [336]), this antiviral shows much promise for the treatment of FIP. The molnupiravir sources that owners have obtained for treatment have been a lot cheaper than GS-441524 [44], although licensed or legal products will need to be available for use in cats in the future.

#### 10.1.4. GC376, a Protease Inhibitor

Protease inhibitors prevent viral replication by selectively binding to viral proteases and blocking the proteolytic cleavage of protein precursors needed for the production of infectious viral particles. Inhibitors that target the 3C-like protease with broad spectrum activity against human and animal coronaviruses have been created [377].

One 3C-like protease inhibitor, GC376, showed strong activity against FCoV in vitro [136] and was effective in treating FIP in an experimental setting; of eight cats with experimentally induced FIP, six remained healthy for an eight month follow-up period [136], although one of these six cats subsequently succumbed to neurological FIP [338].

In a subsequent field trial of natural FIP [338], a cohort of 20 client-owned cats were treated with GC376 at 15 mg/kg SC q 12 h; this was a higher dosage than that used in the original experimental study [136] due to treatment failure in the first cat enrolled in the field trial. Some adverse effects occurred, including injection site reactions and retarded development or the abnormal eruption of permanent teeth [338]. There were no untreated controls in this study and FIP was not confirmed in all cats.

Nineteen of twenty treated cats in the field trail given GC376 at 15 mg/kg SC q 12 h regained health within two weeks of treatment [338]. However, clinical signs recurred one to seven weeks after initial treatment. Relapses no longer responsive to treatment occurred in 12/19 cats within one to seven weeks of initial or repeat treatments. Most of these relapsed cats developed neurological FIP. At the time of study publication, 7 of the 20 treated cats were in remission [338], although this had decreased to 6 at the time of another publication by the same group [38]. Most of the cats that were in remission had presented with effusive FIP at a young age. Cats presenting with neurological signs were excluded from the study due to GC376 not penetrating the CNS [338].

Published retrospective data of cats with suspected FIP [20] mentioned that some cats had been treated successfully with GC376, at 6–8 mg/kg/day for at least four weeks, with or without a nucleoside analogue, but full data on the route of administration, composition of the preparations used, and response to treatment were not provided. Successful treatment with GC376 was also mentioned in one cat in another retrospective study [43], in combination with other agents including rfIFN-ω. Additionally, 5 of the 26 cats that were given molnupiravir as rescue treatment for FIP relapse had received GC376 previously, in combination with GS-441524 [44].

Resistance to GC376 in type II FIP-associated FCoV has been reported [378]; in this in vitro study, resistance was mediated by mutations in the FCoV RNA-dependent RNA polymerase hydrolysis site. Mutated and non-mutated type II FCoV were then used to induce FIP in an experimental in vivo model; the cats infected with the mutated FCoV were resistant to GC376 treatment with two of the three cats dying, where all three cats infected with non-mutated FCoV were successfully treated with GC376 [378]. The two cats that died were positive for FCoV-RNA in all organs sampled, with the highest viral loads in the kidney, followed by the liver and then the cerebellum/pons, i.e., the cats that died following infection with GC376-resistant mutated FCoV did not have a predominant neurological FIP.

In the field GC376 treatment study [338], the 3C-like protease gene sequences of the FCoV infecting a number of cats were compared pre- and post-treatment (samples obtained at post-mortem examination after euthanasia for persistent or relapse of FIP); only one cat showed a change in its 3C-like protease gene sequence. This cat had shown a relapse in FIP with an effusion 30 weeks after starting treatment that comprised two courses totalling 16 weeks. The three gene changes/mutations found in the FCoV infecting that cat were then studied for resistance to GC376 in vitro [379]; one of the mutations conferred a small reduction in susceptibility to GC376, suggesting resistance.

Despite these reports of resistance, protease inhibitors such as GC376 could still offer promise for the treatment of FIP if used in antiviral drug combinations to help avoid the development of resistance [378]. It is hoped that GC376 will be licensed for the treatment of cats with FIP by a USA company within the next few years.

#### 10.1.5. General Considerations for Use of Nucleoside Analogues and Protease Inhibitors

Efficacy in field studies of cats with naturally occurring FIP appears greater with GS-441524 [38,40] than with GC376 [338], as only 6 of 20 cats treated with GC376 remain in remission (quoted in [38]) compared to 25 of 31 cats treated with GS-441524 [38], and 3 of the 4 cats with neurological and ocular signs treated with the higher dose of GS-441524 also went into remission [40]. Both GC376 and GS-441524 treatments cause similar injection site reactions and appear to be relatively safe, although GC376 interfered with the development of permanent teeth in younger kittens. However, GS-441524 can be given orally, in contrast to GC376, and thus, injection reactions can be avoided. The efficacy of GC376 might have been better if all original 20 cats had been treated initially for 84 days [338], rather than with progressively longer treatment courses given following the poor response to two weeks of treatment at the start of the trial.

Drug resistance can occur to antiviral agents, especially with prolonged use and high viral mutation rates [341]. As mentioned above for GC376 treatment, it would be useful to evaluate different types of antiviral drugs, such as protease inhibitors and nucleoside analogues, in combination, to further improve efficacy and/or reduce the possibility of the development of resistance. This is performed for HIV infection and hepatitis C in humans [38]. However, synergistic effects of FCoV antivirals in vitro have not always been demonstrated. In one large study, certain combinations of antiviral drugs showed additive activities against type II FCoV in vitro, but none of the combinations showed more efficacy than GS-41524 or GC376 used as in vitro monotherapies [376]. However, other in vitro studies have suggested synergy between certain antivirals against type II FCoV in vitro, e.g., GC376 and remdesivir [373]. Another in vitro study showed evidence of antiviral synergy between GS-441524 and itraconazole, especially against a type I FCoV [352]. More work is needed in this field and it is likely that a ‘one size fits all’ monotherapy approach for the treatment of FIP is oversimplistic [373].

It has been suggested that the PO, rather than injectable, administration of a nucleoside analogue is more efficacious because it allows the antiviral to go straight to the site of major FCoV replication, and that the inadequate penetration of the gut could lead to drug-resistant FCoV mutants [43,132], although studies confirming the distribution of antivirals based on route of administration have not been published. One study, based on owner-reported survey data, described a high success rate (97% of cats were still alive at the time of publication) in 393 cats treated with unlicensed GS-441524, despite 72% of these cats receiving injectable GS-441524 [23].

There has been concern regarding inducing a relapse of FIP in successfully treated cats with stress that may be associated with neutering or vaccination. Although no published evidence yet exists for the safety of these procedures in previously treated and recovered cats, one of the authors has documented successful vaccination in 23 cats and successful neutering in 21 cats without ill effect (Samantha Taylor and Séverine Tasker, personal communication). In a further two cases, these procedures were performed during GS-441524 treatment, again without ill effect. Similarly, a recent study [17] documented that two cats underwent successful surgery (neutering and enucleation) during remdesivir or GS-441524 treatment. A cost–benefit assessment needs to be performed to decide whether vaccination (including which vaccines [380,381,382]) and neutering are required in an individual cat according to risk, but these procedures do appear to be safe. Feline-friendly handling and methods (including appropriate analgesia for neutering for example) are recommended [383].

Concerns regarding antiviral resistance are behind the reluctance of some to use antivirals in healthy FCoV-infected cats [94], as described below in Section 12.3 on Elimination of FCoV Shedding.

#### 10.1.6. Interferons

Interferons (IFN) have been used in cats with FIP.

Type I IFN is an important cytokine for host defence against viruses, and FCoV has been shown to inhibit its production [384,385], with type II FCoV inhibiting IFN production more strongly than type I FCoV [386]. Recombinant feline IFN-omega (rfIFN-ω), which is licensed in many European countries, is a commercially available monomeric glycoprotein distantly related in structure to IFN-alpha (IFN-α) and IFN-beta (IFN-β) but unrelated to IFN-γ. It has antiviral properties, stimulates natural killer cell activity, and enhances the expression of major histocompatibility complex class I (but not class II) antigens [387]. rfIFN-ω inhibits FCoV replication in vitro [388], but did not abrogate the FCoV shedding in 9 of 11 cats (without FIP) in a shelter [339].

Preliminary results on IFN treatment of cats with FIP were obtained in one uncontrolled trial [329]. Four of twelve cats with FIP treated with rfIFN-ω survived for over 2 years and another four experienced remission, but FIP was not confirmed in any of the cases that survived, although it was confirmed in the cats that died [329]. In a randomised placebo-controlled double-blind treatment trial in 36 cats with confirmed effusive FIP and 1 cat without effusion, rfIFN-ω was used along with systemic (2 mg/kg/day) or intracavitatory glucocorticoids [37]. This study concluded that rfIFN-ω was not effective. However, in a later non-controlled observational study in which glucocorticoids were either not used, or where glucocorticoid dosages were more rapidly tapered, with meloxicam used as an alternative to glucocorticoids to mitigate inflammation, seven cats recovered and other cats experienced prolonged remission following rfIFN-ω treatment [43]. This suggested that rfIFN-ω might have some beneficial effects when used without glucocorticoids, but further controlled studies are required.

As mentioned earlier, in a successfully treated single FIP uveitis case [39], GS-441524 was discontinued after 50 days, when serum AGP had returned to normal, and at this point daily oral administration of 100,000 units of rfIFN-ω was started as follow-up treatment. This cat continued in remission and remains healthy after 3 years having received rfIFN-ω treatment for seven months (Diane Addie, personal communication). The use of both oral and SC rfIFN-ω, usually in combination with, or after, other treatments for FIP, including nucleoside analogues, has been described in a retrospective series of cats with confirmed or suspected FIP in an attempt to avert relapses after stopping the nucleoside analogue treatment [43]. However, many studies have shown excellent survival following nucleoside analogue (including GS-441524) treatment without follow-up rfIFN-ω [17,19,24,31,38,40,45], so the need for rfIFN-ω treatment is still in question.

When used, dosages of rfIFN-ω have varied [39,43,332]. It is sold in vials of 10 million (10^7^) units, and one vial is reconstituted with 1 mL of saline diluent; 0.1 mL of the diluted stock solution then contains 1 million units of rfIFN-ω. Once diluted, rfIFN-ω maintains its potency in the fridge for up to 3 weeks, so the rest of stock solution should be frozen if not in use, where it can be kept frozen for up to 6 months. Two dosage regimes for rfIFN-ω have been reported:

- One million (10^6^) units/kg SC or PO q 48 h for up to five doses, and then twice a week until rfIFN-ω treatment is stopped [43].
- 100,000 (10^5^) units per cat SC or PO q 24 h until rfIFN-ω treatment is stopped [39,43]; 0.1 mL of previously diluted stock solution containing 1 million units of rfIFN-ω a is diluted again with 4.9 mL of saline diluent. Hence, 0.5 mL of the total 5 mL of the new stock solution now yields 100,000 units.

An in vitro study evaluating the effect of rfIFN-ω with hydroxychloroquine found increased antiviral activity of hydroxychloroquine against type I, but not type II, FCoV infection of cell cultures with rfIFN-ω [350], suggesting that combination treatment could be considered, although in vivo studies are needed.

Feline fibroblastic IFN-β also inhibits FCoV replication in cell culture [389], but no in vivo studies exist.

Human IFN-α was effective against an FIP-associated FCoV strain in vitro [390] but in a placebo-controlled treatment study of 74 specific pathogen-free cats in which FIP was induced experimentally, neither the prophylactic, nor therapeutic, administration of high doses (10^4^ or 10^6^ IU/kg) of IFN-α, feline IFN-β (10^3^ IU/kg), the immunomodulator *Propionibacterium acnes* (0.4 mg/cat or 4 mg/cat), or a combination, significantly reduced mortality in treated versus untreated cats [358]. However, in the cats treated with 10^6^ IU/kg IFN-α in combination with *P. acnes*, the mean survival time was prolonged, but only by a short amount [358]. As an explanation for the limited efficacy of IFN-α, it has been suggested that ORF-7-encoded accessory protein 7a of FIP-associated FCoV strains can act as a type I IFN antagonist and counteract the IFN-α-induced antiviral response [385]. When human IFNs are injected (i.e., SC as per dosage regimes above) into cats, antibodies are raised against them, limiting their longer-term usefulness, although when administered PO, no antibody formation occurs (but systemic efficacy is likely limited). A cat with panuveitis and skin lesions due to FIP treated with human IFN-α and prednisolone survived 10 weeks before euthanasia [225].

#### 10.1.7. Anti-Malarial Compounds

Several anti-malarial drugs have been investigated for their antiviral effects. An anti-malarial Chinese herbal extract of unknown identity was found to inhibit the in vitro growth of FIP-associated FCoV [391]. Chloroquine is too toxic for cats [340,349], and hydroxychloroquine, although used in in vitro studies only, has been suggested as a less-toxic alternative to chloroquine [350].

Another agent that shows promise in cats is mefloquine, and although the full mechanism of its antiviral action is not known [340,343], it is believed to act as a nucleoside analogue [341]. Studies have been published on mefloquine’s hepatic metabolism using an in vitro model [342], its pharmacokinetics in healthy cats [343], and its plasma protein-binding properties in the plasma of healthy cats and cats with FIP [344]. Although studies are needed on its efficacy in cats with FIP, veterinarians in Australia (Richard Malik, Sally Coggins and Jacqueline Norris, personal communication) are using oral mefloquine to treat cats with FIP when finances prohibit the use of a full course of, or increased dosage of, more effective antivirals, such as GS-441524, as mefloquine is more affordable [332]. Used dosages are shown in Table 2. However, mefloquine is probably only effective as adjunct treatment and it can cause vomiting if not given with food, but it generally appears safe in healthy cats.

#### 10.1.8. Cyclosporine A

Cyclosporine A can act as an antiviral drug as it binds to cellular cyclophilins thereby inhibiting calcineurin, which is required by many viruses for replication [345,346]. Cyclosporine A inhibits FCoV replication in vitro [345] and was also associated with a reduction in pleural fluid volume and a decrease in viral load in a cat with FIP [347] (Table 2), but the cat succumbed to FIP 264 days after treatment initiation. Thus, cyclosporine A might be an option in combination with other therapeutic agents, but more studies are needed.

#### 10.1.9. Curcumin

Curcumin, a derivative of turmeric, has antiviral and anti-inflammatory properties. Curcumin-encapsulated chitosan nanoparticles (Cur-CS), created to increase the bioavailability of curcumin, were evaluated in vitro and found to decrease the expression of pro-inflammatory cytokines TNF-α, interleukin- (IL-) 6, and IL-1β produced during infection of cell cultures with an FIP-associated FCoV as well as to inhibit viral replication [348]. The same study confirmed the enhanced bioavailability of Cur-CS over curcumin in pharmacokinetic analysis in healthy cats. However, another in vitro study failed to find any inhibitory effect of curcumin on FCoV proliferation [340]. Thus, further studies on this agent are required.

#### 10.1.10. Miscellaneous Antiviral Treatments

Some other drugs have only been investigated in vitro, and in vivo efficacy is unknown, such as vidarabine [359], which inhibits polymerase activity; nelfinavir, a commercially available protease inhibitor of human immunodeficiency virus [355]; *Galanthus nivalis* agglutinin, a carbohydrate-binding agent that binds to FCoV-glycosylated envelope glycoproteins, thereby inhibiting viral attachment to the host cell [355,392]; K31, a novel compound that binds and alters the conformation of the FCoV nucleocapsid protein [393]; plant-derived flavonoids (including isoginkgetin) [394]; and ERDRP-0516, a non-nucleoside inhibitor of the RNA-dependent RNA polymerase [395]. One study has shown evidence of antiviral synergy between GS-441524 and itraconazole in vitro, especially against a type I FCoV [352]. Some drugs are effective in vitro, but are too toxic for cats, such as ribavirin [356,357,390].

Promising experimental approaches include inhibition of the binding of FCoV spike protein to receptors on the host-cell membrane that mediates fusion of the viral envelope with host-cell membranes [377,396], circular triple helix-forming oligonucleotide RNA targeting viral RNA [397], cholesterol synthesis and transport inhibitors inducing cholesterol accumulation in cells and thereby inhibiting FCoV replication [351,352,398,399] and small interfering RNAs (siRNA) leading to RNA interference and thus, inhibition of virus replication [341,400,401].

### 10.2. Immunomodulatory Drugs for FIP

Immunomodulators are often used in cats with FIP. The idea behind these treatments is generally stimulation of the immune response towards a CMI response to FCoV in FIP. An early effective T cell response has been suggested as protecting from the development of FIP [192]. However, this is a complex area and, whilst there is a lack of documented efficacy in well-controlled studies [402,403], general recommendations cannot be made. Some old case reports suggest some effect through immunomodulator treatment (e.g., tylosine, promodulin, acemannan) but FIP was not always confirmed [12,402,404,405,406,407].

Table 5 shows immunomodulatory treatments that have been used for FIP.

#### Polyprenyl Immunostimulant

Polyprenyl immunostimulant (PI) is a drug that has shown promise for the immunomodulation treatment of FIP. PI is a commercially available oral agent that is given three times a week and is thought to act by upregulating Th-1 cytokines and CMI by upregulating innate immunity via toll-like receptors [330,410,414].

Although licensed for the treatment of feline herpesvirus (FHV) [415], it has been used off-label to treat FIP at higher dosages. In a case series of three non-effusive FIP cats (confirmed by histopathology in only one of the three cats), PI was associated with prolonged survival [410]. In a field study, treatment with PI was evaluated in 60 cats that were suspected to have FIP without effusion by primary care and specialist veterinarians, but confirmation of FIP was not established in all cats and no untreated controls were included [330]. Of the 60 treated cats, 16 survived over 100 days; of these, 8 survived over 200 days, including 4 who survived over 300 days. Veterinarians of treated cats that survived over 30 days reported improvements in clinical signs and behaviour. The survival times were significantly longer in cats that were not treated with systemic glucocorticoids concurrently, although topical ophthalmic glucocorticoids did not appear to affect survival like systemic glucocorticoids [330].

PI was used (amongst other treatments) in three cats that recovered from FIP and one who succumbed in a descriptive study [43]. Another study [212], which evaluated 29 cats with FIP (86% did not have effusions) that had received PI for at least 365 days, reported a mean survival time of around eight years. Here, diagnosis of FIP was based on a positive FCoV IHC or RT-PCR result, i.e., a diagnosis of FIP was confirmed or very likely [211]. A low haematocrit and/or low A:G ratio (or which stayed low with treatment) was a negative prognostic indicator for response to PI [212]. Prednisolone was only given to two cats in the study, but both of these cats survived for less than the mean survival time. Some surviving cats stayed on PI treatment throughout the study (some moved onto a maintenance dosage after a year of treatment) whilst others had their PI treatment stopped once their haematocrit and A:G ratio had normalised [212].

The dosages of PI used in different studies [43,212,330,410] have generally comprised 3 mg/kg PO three times a week or q 48 h. In one study [212], the dosage was reduced to 3 mg/kg once or twice a week only as a maintenance dosage at about a year after diagnosis.

Thus, treatment with PI might hold some promise for cats with FIP but controlled studies to compare PI and antivirals, and/or PI given as adjunct treatment to antivirals, are required. PI enhances CMI and therefore is more likely to be effective in cats without effusions which have better CMI than cats with effusions that have more impaired T cell immunity [212].

### 10.3. Supportive Treatments for FIP, including Anti-Inflammatories and Drainage

Table 5 outlines agents that have been used in the supportive treatment of FIP. It is important to realise that supportive treatment should be tailored to the needs of the individual cat. The remarkable response seen in the cats with FIP treated with oral GS-441524 in one study [24] was partly attributed to the intensive supportive care provided during hospitalisation of the cats over the first eight days of treatment, comprising IV fluid therapy, appetite stimulants, anti-emetics and analgesia. Good reviews on supporting the inappetant hospitalised cat [409], as well as minimising stress in hospitalised cats [383,408], are available. Rehydration and the maintenance of fluid balance are important in dehydrated inappetant cats, necessitating fluid therapy. Vitamin supplementation (particularly B12) can be given [23,43,332], although the value of supplementation in the absence of hypocobalaminaemia is not known [409]. In cats that are hypoglycaemic, dextrose supplementation may be indicated [45].

The importance of supportive care also highlights the need for veterinarians to be involved in the treatment of cats with FIP. As described earlier, the use of illegally obtained antivirals by owners often means that veterinarians are not involved in the care of cats undergoing treatment for FIP [23] due to legislative and legal fear in veterinarians if they deal with these cats. This creates a disconnect between owners and veterinarians. However, it is possible for vets to give supportive care to cats in this situation for welfare reasons, as long as documentation is created to confirm there has been no veterinary involvement in the advising, obtaining or prescribing of the illegal drugs.

Although glucocorticoids, for inflammation and/or immune-mediated pathology, have commonly been used to treat FIP palliatively in the past, a positive effect has not been substantiated. Two separate double-blind controlled studies that evaluated rfIFN-ω [37] and propentofylline [411] as treatments for FIP gave all of the cats (both those in the treatment and the control groups) glucocorticoid treatment. The cats given the additional drugs did not survive any longer than those given glucocorticoids only but of importance is that those on glucocorticoids only survived for a median of eight days, confirming a poor outcome with glucocorticoid treatment alone in these cats [369]. In another study, cats without effusion treated with both systemic glucocorticoids and the immunomodulator PI had poorer survival than those treated with PI alone [330], again suggesting a negative effect of glucocorticoids, although a definitive diagnosis of FIP was not established in all cats. In the study based on owner-reported survey data, describing 393 cats treated with unlicensed, mainly injectable, GS-441524, high success rates were reported despite steroids (presumed glucocorticoid; but not specified in the owner survey) being used in 38% of cats [23]. However, concern over the use of systemic glucocorticoids to treat FIP has been raised, and discontinuation of glucocorticoids within one to two weeks of starting remdesivir, with or without transition to GS-441524, has been reported [17] and a preference stated for NSAIDs, over glucocorticoids, if an anti-inflammatory treatment is required [43]. The latter recommendation was made due to a better success rate (92%; 11/12) in cats that were not given prednisolone compared to those that were (44%; 11/25); various other treatments were also given to these cats. Despite this, adverse effects of NSAIDs have to be considered, i.e., blood pressure and kidney function have to adequate, and the cat should be eating before receiving NSAIDs. In some countries, such as Germany, metamizole is used in place of NSAIDs as an analgesic with anti-fever and anti-inflammatory properties [24]. In the study [45] describing 32 cats with effusive or non-effusive FIP treated with a combination of remdesivir and GS-441524, only 3 cats in total received systemic steroids (two hydrocortisone, one prednisolone), with 2 of these surviving; the surviving cat given prednisolone had received a decreasing dosage from 0.8 mg/kg/day over 16 days.

If uveitis is present, it is important to regularly monitor intraocular pressure (feline reference range 15–25 mmHg) as although low in uveitis, an increase could signify the development of secondary glaucoma, necessitating specific treatment [416]. If anisocoria is present, this can be due to potential posterior synechiae, raising the risk of secondary glaucoma in the face of prolonged (and low-grade) uveitis (Ursula Dietrich, personal communication). FIP antiviral treatment will help control intraocular inflammation by the treatment of the underlying disease [39,330], but there is believed to be an immune-mediated component to the uveitis that may require topical anti-inflammatory treatment (e.g., prednisolone acetate drops) [416]. One case report has described the use of topical ocular prednisolone acetate in a cat with uveitis receiving antiviral GS-441524 [39]. When needed, this can be used alongside antivirals, and it can be tapered to a maintenance dosage over time. Miosis may require atropine treatment. The cause of uveitis in FIP is thought to be a result from granuloma formation, pyogranulomatous vasculitis and possibly immune complex deposition, so only immunosuppressive topical corticosteroids usually work in those severe fibrinous uveitis cases seen in FIP, rather than topical NSAIDs (Ursula Dietrich, personal communication). Residual scarring can occur following chronic uveitis and if the eye is non-painful with a normal intraocular pressure, enucleation is not required. However enucleation might be required due to pain and glaucoma [416].

If a pleural effusion results in dyspnoea, drainage is indicated to provide some relief to the cat. Drainage does not tend to produce long-term relief for ascitic patients but is indicated if the degree of abdominal effusion is compromising respiration.

If indicated, cats can also be treated with broad-spectrum antibiotics (e.g., if bacterial translocation is suspected) and supportive therapy (e.g., fluids if dehydrated) [417].

Pentoxyfylline or propentofylline has been given to cats with FIP because they can down-regulate pro-inflammatory cytokines which are thought to increase vasculitis. However, in a placebo-controlled double-blind study in cats with confirmed FIP, there was no significant difference in survival time, quality of life, or any clinical or laboratory parameter in cats treated with propentofylline versus cats receiving placebo. However, all cats received glucocorticoids in this study [411], and further studies without glucocorticoids would be valuable.

A thromboxane synthetase inhibitor (ozagrel hydrochloride) that inhibits platelet aggregation and cytokine release was used in two cats in an uncontrolled study, with some improvement of clinical signs [413], but a follow-up study was unsuccessful [387].

A placebo-controlled study in a small number of cats (three treated, three placebo) in an experimental model of FIP found a possible beneficial effect of treatment with antibodies acting against feline TNF-α [412]. In that study, progression to FIP was prevented in two of the three cats treated with these antibodies, whereas all three cats developed FIP in the placebo group. TNF-α is thought to be involved in FCoV replication in macrophages [186] and contributes to development of clinical signs in cats with FIP. An uncontrolled very small study used anti-human-TNF-α antibody treatment alongside itraconazole [190]; only 3 of the 10 cats inoculated in this experimental study developed FIP, and 2 of these 3 treated cats improved with anti-human-TNF-α antibody and itraconazole treatment. No field studies have been conducted so far.

**Summary of Section 10: Treatment of FIP** The **availability of effective curative antiviral treatments for FIP**, particularly the nucleoside analogue **GS-441524**, has totally changed the landscape of this previously fatal disease. These treatments **act quickly**, allowing for the diagnostic **trial treatment** of cats in which **FIP is very likely**. However, treatment is **often expensive, not licensed and not available legally in many countries**, which **complicates its use**. Some countries have access to veterinary compounded GS-441524 products. In others, owners source antivirals themselves online, but the quality, purity, and concentration of active ingredients in these preparations is unknown, although they are clearly effective, based on published studies. **Success rates of 81% to 100%** have been reported in cats treated with different preparations of compounds believed, or known, to contain **GS-441524**. In initial studies, GS-441524 was administered by subcutaneous (SC) injection, which was often painful, but **oral** preparations are now available, which are very effective, are cheaper and better tolerated that SC injections. Most studies have used **84-day treatment courses, but shorter courses might be also effective**. Non-clinically significant transient adverse effects of GS-441524 can include elevations in ALT (hepatoprotectants are sometimes given but are unlikely to be needed), lymphocytosis, and eosinophilia. **Weight gain** has been cited as a simple long-term measure of treatment efficacy too as it is easy to measure using paediatric weighing scales, every one to two weeks, allowing for an increased dose to be calculated to maintain the appropriate dosage despite weight gain during recovery. Hyperbilirubinaemia, hyperproteinaemia and leucocyte abnormalities typically normalise within a few weeks, but hyperglobulinaemia might take longer to normalise. Overall, a **good appetite** and/or **activity level**, a **higher temperature**, a **lower bilirubin concentration** and fast **normalisation of α1-acid glycoprotein (AGP)** appear to be prognostically useful to predict survival with GS-441524 treatment. FCoV antibody concentrations are not useful to track response to treatment. **Abdominal lymphadenomegaly** has been reported following effective GS-441524 treatment but does not signify FIP relapse. **Remdesivir** is a nucleoside analogue and the prodrug of GS-441524. A human-licensed preparation is available, as well as a veterinary compounded product in some countries. Remdesivir is injected, either intravenously or SC, but SC administration is painful. Most veterinarians thus favour oral GS-441524 treatment for FIP, unless remdesivir is the only antiviral available and/or the cat is unable to tolerate oral medication (e.g., due to being very sick). No comparative controlled studies currently exist on the efficacy of remdesivir and GS-441524. **Molnupiravir** is another oral nucleoside analogue. It has shown promising results as a **first-line agent and a rescue agent for cases that relapse**. A human-licensed preparation is available, but rules vary in different countries as to whether it may be used in cats. More comparative studies are required. **Protocols** have emerged on how nucleoside analogues are used to treat FIP; these usually include recommendations for higher dosages in cats with ocular or neurological signs. **Vaccination and neutering** have both been performed during, or following, successful treatment of FIP with nucleoside analogues, in cats in which these procedures have been deemed necessary. No relapse of FIP has been recorded although employment of feline-friendly methods is recommended to minimise stress. **GC376** is an injectable protease inhibitor that has been used successfully for the treatment of FIP. Dentition adverse effects were reported. No legal preparations are currently available although it is hoped that a cat licensed product will be available in the future. Some veterinarians have used **mefloquine**, recombinant feline interferon-omega (**rfIFN-ω**) and/or **polyprenyl immunostimulant** (PI) for the treatment of FIP, although none of these are as effective as the nucleoside analogues. **Oral mefloquine** is an affordable human-licensed product that has been used anecdotally for FIP, but no published FIP treatment studies exist. It might be useful as adjunct treatment or in cases where other more effective antivirals cannot be used due to cost or availability. It is given with food to avoid vomiting as a side effect. **rfIFN-ω** is licensed for use in cats in some countries and, for FIP, it has been used most recently following antiviral therapy with GS-441524 to prevent relapse. However, controlled studies are needed to confirm efficacy of, and need for, rfIFN-ω, as many studies have shown excellent survival following nucleoside analogue (including GS-441524) treatment without follow-up rfIFN-ω. **PI** might be helpful in the treatment of FIP without effusions although response to treatment is slow, over several months. It has been found that concurrent systemic glucocorticoid treatment should be avoided with PI, as this worsens prognosis. Although more studies are needed, **systemic glucocorticoids should probably be avoided in the treatment of FIP**, although **topical steroids for uveitis are permitted**. **Veterinary supportive care** (e.g., intravenous fluids, appetite stimulants, anti-emetics, analgesia, vitamin B12, non-steroidal anti-inflammatories) is very **important in the recovery of cats that are very sick due to FIP**. However, veterinary support is often not sought by owners who have obtained antiviral drugs illegally for their cats as veterinarians are unable to advise, obtain or prescribe illegal drugs, leading to a disconnect between owners and veterinarians. It is possible for vets to give supportive care to cats in this situation for welfare reasons, as long as documentation is created to confirm there has been no veterinary involvement in the illegal drug procurement or administration. Further details on antiviral, immunomodulatory and supportive treatments for FIP (including dosages) are given in Table 2 and Table 5, which should be used in conjunction with this summary.

## 11. Vaccination

### 11.1. Efficacy of FIP Vaccines

At present, there is one intranasal FIP vaccine commercially available in the USA and in some European countries. It contains a temperature-sensitive mutant of the type II FCoV strain DF2. Type I coronaviruses are, however, more prevalent in the field in most countries [5,64,75]. The vaccine aims to induce local mucosal immune responses through the induction of IgA and CMI. However, it also induces the development of systemic antibodies against FCoV, although usually with low titres.

The efficacy of this vaccine is in question and its use is not recommended by ABCD. Results from experimental studies have been inconsistent, with preventable fractions between 0 and 75% reported [418,419,420,421,422,423,424]. Results from field studies have been equally inconsistent [425,426,427]. No difference in the development of FIP between the vaccinated and placebo groups was found during the first 150 days after vaccination when the vaccine was used in Persian breeding colonies [425]. However, after 150 days, significantly fewer cases of FIP occurred in the vaccinated cats compared to the placebo group [425]. In another trial, a preventable fraction of 75% was found when the vaccine was tested in a large cat shelter in the USA [427], although the published study description was very short, making it difficult to interpret the study fully. In this study, all kittens were antibody-negative prior to vaccination. The conclusion is that the vaccine is likely not effective in antibody-positive cats that have already been exposed to FCoV. The ADE of infection that was a feature of some experimental vaccine trials [422,423,424], where more vaccinated than control cats developed FIP, has not been observed in field studies, suggesting that the vaccine can be considered safe [425,426,427]. However, ADE is still of concern in the development of vaccines.

### 11.2. Use of FIP Vaccines

The ABCD considers the presently available FIP vaccine to be non-core, with questionable efficacy [59]. There is no benefit in the use of this vaccine in FCoV antibody-positive cats, which severely limits its use, as many cats are already FCoV antibody-positive at the age (at least 16 weeks) that the vaccine can be administered (Table 1). FCoV antibody-negative kittens could potentially benefit from vaccination, particularly if they subsequently enter a FCoV-endemic environment and thus would be at risk of developing FIP. The fact that in multi-cat environments most kittens are already infected at the age of 16 weeks is a major limiting factor [3,122,126].

#### 11.2.1. Primary Course

If vaccination is to be given, the first dose should not be given before 16 weeks of age, with a second dose being given three weeks after the first dose.

#### 11.2.2. Booster Vaccinations

If primary vaccination has been performed, annual boosters can be considered, and, although studies on duration of immunity are lacking, it is thought to be short-lived [64].

**Summary of Section 11: Vaccination:** An **intranasal vaccine** for FIP is available in some countries for cats **aged 16 weeks or over**. However, it should only be given to cats that have not yet encountered FCoV infection, which is difficult as FCoV infection is widespread in cat populations. Additionally, its **efficacy has been questioned**. Its use is **not recommended by ABCD**.

## 12. Control of FCoV Infection and FIP

### 12.1. Reducing FCoV Transmission

Since FCoV is transmitted predominantly via the faecal-oral route, hygiene is the mainstay of FCoV (and therefore FIP) control in any multi-cat environment. FCoV infection is maintained in a household by continual cycles of infection and re-infection [64,128], with the source of infection usually being faeces in the litter tray. Rarely is FIP a problem amongst cats leading an indoor–outdoor lifestyle or in stray cats that bury their faeces outside, unless these cats originate from multi-cat environments [18]. Indeed, in multivariable analysis, Italian stray colony cats were found to be less likely to be FCoV antibody-positive compared to owned cats [147].

The goal in every cat household must be to reduce the FCoV infection pressure and risk of transmission. This can be achieved by keeping cats in small well-adapted groups (not more than three per room has been suggested), observing strict hygiene, and providing outdoor access if possible [46,59]. If outside access is not possible, enough litter trays should be provided, i.e., one more than the number of cats present, in the areas that the cats have access to. Litter trays should be positioned in different rooms away from food and water bowls. Litter trays should have faeces removed at least twice a day and be completely emptied at least weekly and cleaned using detergent. Utensils should be cleaned daily. One study [428] suggested that a clumping bentonite-based Fuller’s earth cat litter, which tracked minimally, was associated with a reduced FCoV load in a multi-cat household compared to another type of Fuller’s earth litter. This effect was believed to be due to a binding effect of the clay in the litter as well as its non-tracking property, helping reduce spread. Further studies are required.

Although FCoV is only rarely shed in the saliva, food and water bowls should still be cleaned daily using detergent or in a dishwasher at a cycle of at least 60 °C, because of the risk of indirect transmission from cat litter-contaminated fomites.

### 12.2. Managing FCoV Shedders

FIP is especially a problem of cats kept in larger groups, particularly in breeding catteries and rescue situations [429], due to the high prevalence of FCoV infection in multi-cat environments (Table 1).

Type I coronavirus faecal shedding in cats occurs over several months or is sometimes lifelong, especially in multi-cat households [5,64,126]. Cats can be identified as shedders by the submission and analysis of faeces (preferable) or a rectal swab by FCoV RT-qPCR. The laboratory performing the testing should provide the FCoV load present (or a RT-PCR cycle threshold value) and an interpretation of the results. However, a universally accepted protocol for the identification of shedders does not exist. Testing faecal samples collected weekly on four occasions [59,133] has been recommended for the detection of shedders, although other reports have described testing at least three faecal samples collected at between 5- and 28-day [123,124], or 30-day, intervals [135].

The identification of cats that are shedding, or are inferred to be shedding, FCoV, and their separation from cats believed to be non-shedders, has been suggested as a method for reducing transmission rates [59,430]. However, one must remember that the results of screening for FCoV shedders gives only a temporary picture and results can change over time, necessitating retesting. Additionally, the recommendations on how to reduce the FCoV infection pressure, described above under Section 12.1 on Reducing FCoV Transmission, should be strictly enforced. Additionally, FCoV-infected cats should not be exposed to stressful situations to try and help reduce viral load; it is known that FCoV shedding increases in cats after entering a shelter, which is believed to be due to stress [431].

The use of FCoV antibody testing on blood samples at a single time point, instead of repeated faecal sampling for RT-qPCR, is less useful for identifying FCoV shedders [135], despite the finding of a positive correlation between antibody titres and both the likelihood and frequency of faecal FCoV shedding and faecal viral load [5,135]. The following data help illustrate this: 15 of 64 antibody-positive cats did not have FCoV RNA in any of their 4 sequentially (at intervals of 5–30 days) collected faecal samples, whilst 2 of the 18 antibody-negative cats had FCoV RNA in all of their 4 sequentially collected faecal samples [135]. Similarly, another study identified FCoV RNA in 10 of 81 faecal or rectal swab samples collected from antibody-negative cats [5]. Thus, serum antibody status cannot reliably predict faecal shedding. In some circumstances, this lack of antibodies in shedding cats might be explainable by early FCoV infection, as antibody development can take 7 to 28 days post-infection [85,203].

### 12.3. Elimination of FCoV Shedding

Using antivirals, such as GS-441524, to clear FCoV infection in cats without FIP [132,432] is very controversial for two reasons: one is the potential risk that doing so will cause drug-resistant escape mutant viruses to develop, and the second is the concern that clearing a household of FCoV is difficult to achieve and maintain.

Regarding the appearance of drug-resistant escape mutant viruses: such mutant viruses have been demonstrated in vitro with the protease inhibitor GC376 [378] but have yet to be demonstrated in vivo in cats treated with oral GS-441524. Possible indirect evidence for resistance to GS-441524 occurred in 1 of 26 cats with FIP that was treated with a very low dosage (2 mg/kg) of injectable GS-441524 SC; in this cat, FCoV RNA levels did not decrease over 26 days (in ascitic fluid over 9 days, sample type thereafter not stipulated), which the authors attributed to a ‘failure to halt virus replication’ [38]. Drug resistance can develop in situations where drugs are used for a long duration [433]. Another explanation for the failure of this cat to respond to the GS-441524 treatment was that the dosage could have been too low to cross the blood–brain barrier; indeed, the cat developed ocular and neurological signs before dying, which supports this hypothesis.

The second objection to using antivirals in cats without FIP is the concern that eradication of FCoV from multi-cat households is difficult and complex and cannot be maintained because the virus is ubiquitous and so infection can be easily re-introduced. Indeed, in an FCoV eradication study, one household (which failed to meet the inclusion criteria for the study, but in which FCoV eradication was performed) had FCoV re-introduced with the introduction of many FCoV-infected kittens. Therefore, this is a real risk, even though other households did successfully introduce previously FCoV-infected cats through quarantine, isolation and GS-441524 treatment, thus preventing re-infection of their other cats [132].

For people wishing to eradicate FCoV from their household, the authors would urge them to bear in mind that FCoV infection is often self-limiting in households of fewer than ten cats [167]. In some situations, FCoV eradication from a household for FIP prevention can be achieved by excellent hygiene, quarantine and testing prior to introducing cats or kittens into households. The FCoV load can also be reduced in multi-cat households using clumping bentonite-based Fuller’s earth cat litter [428] and avoiding stress [431], as described earlier. Itraconazole [132] and rfIFN-ω [339] reduced, but unfortunately did not abrogate, coronavirus shedding. Whether mefloquine can halt faecal FCoV shedding is currently unknown.

### 12.4. Further Considerations in Breeding Catteries

Breeding catteries are those households in which the reduction of FCoV infection pressure is of particular importance. A study of 37 breeding catteries in Germany, which performed RT-PCR on faecal samples collected from cats in the catteries, did not find any FCoV-free catteries [123], showing how highly prevalent FCoV is in such environments. In this study, in which all of the breeding households had at least five cats, using multivariable analysis, they found that only having cats of less than one year of age was associated with an increased risk of FCoV shedding; management and husbandry measures (e.g., thoroughness of cleaning, number of litter trays, cleaning and disinfection frequency), surprisingly, were not associated with prevalence of faecal shedding [123]. A subsequent study [124] that evaluated some of the same cats from the original 37 breeding catteries in Germany [123] reported that 125/222 (56%) cats were RT-PCR-positive on faeces for FCoV RNA in at least 3 of the 4 faecal samples taken, i.e., these cats were believed to be persistent shedders, albeit with the understanding of the limitation of testing the cats for up to four months only. The same study [124] reported that 55/222 (24%) cats had all four faecal samples testing negative for FCoV RNA, i.e., these cats were deemed to be non-shedders. Multivariable analysis found that persistent FCoV shedding was significantly associated with breed (Persians were at increased risk) and increased frequency of cleaning of litter trays per day. Conversely, non-shedding status was also significantly associated with breed (Birman and Norwegian Forest more likely to be non-shedders), as well as having fewer cats in the household and with a lower frequency of disinfection of litter trays per month. These results are difficult to explain as one would expect that more frequent cleaning, and increased disinfection, of litter trays would be associated with a reduction in FCoV shedding. It may be that FCoV shedders are more likely to have diarrhoea, as has been reported [154], stimulating more frequent cleaning or disinfection of litter trays, but faecal scoring was not performed in this study and so this cannot be confirmed or refuted [124].

Keeping no more than three well-adapted cats per room (and keeping such cat groups stable) and providing outdoor access if possible [46] is also helpful.

Special measures in kittens can be considered. FIP usually occurs after the kittens have left the breeder and are in a new household [434]. It has been suggested that most kittens are considered protected from FCoV infection by maternally-derived antibodies until they are five to six weeks [119] or even up to 10 weeks of age [128]. In some studies, FCoV transmission has been prevented by isolating pregnant queens two weeks before birth and then moving their kittens away to a clean environment away from other cats when they are five to six weeks old and maintaining them there until they go to a new home [3,181]. For this method to succeed, the breeder is required to follow strict quarantine hygiene methods. However, the procedure failed in another study in which kittens were found to shed FCoV already as early as at the age of two weeks [122]. Veterinary behaviourists also advise against early weaning due to socialisation problems arising in these kittens [435,436,437], and this, together with the laborious nature of early weaning, makes it unpopular amongst most veterinarians [59].

Although commercial PCR tests are available which purport to detect cats that are resistant to FIP (e.g., feline IFN-γ gene SNPs discussed briefly in Section 5 on Immunity), they are not recommended as a basis for breeding decisions. Positive selective breeding for ‘resistance to FIP’ in a colony of laboratory cats was shown to decrease the survival of the offspring after intraperitoneal inoculation with FIP-associated FCoV [173]. The diminished resistance to FIP in these cats was associated with decreased genomic heterozygosity.

### 12.5. Further Considerations in Rescue Facilities, Shelters and Boarding Catteries

Preventing FCoV infection in rescue facilities, shelters, and boarding catteries is extremely difficult. In catteries and shelters with multiple cats, FCoV infection is virtually always present [143] (Table 1). Incoming cats should be quarantined for a minimum of three weeks to allow for the emergence of any incubating infections. As mentioned previously, after entry into a shelter, the shedding of FCoV increases dramatically within one week amongst cats that were already infected at entry; this was believed to be due to stress because more than one-half of initially FHV-negative cats began shedding FHV a week later, and latent FHV recrudescence occurs due to stress [431]. Stress reduction is of particular importance, as stress can lead to increased virus production and a risk of the development of FIP. Cats who develop FIP frequently have a history of stress prior to presenting with FIP [18,36,248], probably due to the immunosuppressive effects of stress, allowing increased virus production.

Strict hygiene protocols for care workers, cleaning and disinfection must be enforced to reduce FCoV contamination and viral spread. Special care should be given to cleaning litter trays with boiling water or steam between use in different cats, having litter trays and scoops dedicated to each cat pen, and avoiding fomite transmission on cleaning utensils, such as brushes.

Ideally, cats should be kept in small groups of three or fewer cats per room [46] and with limited exchange of animals between groups. New catteries should be designed with infectious disease control and stress reduction as priorities [438,439,440]. More information on control of infectious diseases in shelters can be found in the ABCD guidelines on prevention and management of feline infectious diseases [441] and infectious diseases in shelter situations and their management [442].

### 12.6. Management of FCoV-Infected Cats without Clinical Signs

Stress (e.g., surgery, boarding, adoption) [18,36,248] or immunosuppression caused by co-infection with immunosuppressive viruses (e.g., FIV or FeLV) [81,443] or any treatment inducing immunosuppression [134] might increase the risk of FIP development in FCoV-infected cats. However, cats might have diseases that require immunosuppressive treatment despite the presence of FCoV infection. The minimisation of stress and avoidance of secondary infections are therefore important to prevent the development of FIP in FCoV-infected cats.

As mentioned in Section 11.1 on Efficacy of FIP Vaccines, FIP vaccination is not useful in FCoV-infected cats.

The question has been raised whether FCoV-infected cats should receive other vaccinations, since vaccination was identified as a stressor preceding onset of FIP in one study [18]. However, no evidence exists to support that FCoV-infected cats should be vaccinated less often than uninfected cats. Therefore, until the contrary has been demonstrated, FCoV-positive cats not showing any signs of illness should receive vaccination similarly to uninfected cats, with a cost–benefit assessment performed to decide whether vaccination (including which vaccines) [380,381,382] are required.

### 12.7. Maintaining a FCoV-Negative Status

If a household has achieved a FCoV-negative status, efforts should be made to keep it FCoV-free [132] by testing new cats and kittens for FCoV infection preferably before bringing them into the household or, if that is not possible, by keeping them in quarantine until they are FCoV-free (see Section 12.2 on Managing FCoV Shedders and Section 12.6 on Management of FCoV-Infected Cats Without Clinical Signs). Certain geographical areas, such as the Falkland Islands [140], have been maintained as FCoV-free using these principles.

**Summary of Section 12: Control of FCoV and FIP** As FCoV is transmitted predominantly via the faecal–oral route, **hygiene is the mainstay of FCoV** (and therefore FIP) **control. FCoV infection is maintained in households by** continual cycles of infection and re-infection **and is less of a problem amongst cats with access outdoors that bury their faeces outside. A reduction of FCoV infection pressure can also be helped by** not keeping more than three well-adapted (consistent) cats per room and providing outdoor access. **If outside access is not possible**, the number of litter trays **should be one more than the number of cats present. Litter trays should be positioned in different rooms, away from food and water, have faeces removed twice a day and completely cleaned once weekly.** Non-tracking clumping bentonite-based Fuller’s earth cat litter **can be helpful to reduce FCoV spread**. **The identification and separation of FCoV shedders** can be helpful for reducing transmission rates of FCoV in a household. **No universally accepted protocol for identification of shedders exists,** and testing results represent the situation at only that timepoint, with changes in results occurring over time. Although positive correlation exists between FCoV serum antibody titres and the likelihood and the frequency of faecal FCoV shedding, as well as the FCoV faecal viral load, this relationship is not straightforward. Serum antibody-negative cats can be positive for FCoV RNA in faeces and serum antibody-positive cats can be negative for FCoV RNA in faeces. The use of nucleoside analogues, such as **GS-441524, to eliminate FCoV shedding** in cats without FIP is very **controversial**. Some suggest there is a potential risk of development of drug-resistant escape mutant FCoVs, and are concerned that clearing a household of FCoV is difficult to achieve and maintain, due to the high prevalence of FCoV infection in cat populations. Those wishing to eradicate FCoV from their household should be reminded of the importance of both **hygiene and keeping cats in small groups**, as well as other measures to reduce FCoV load (e.g., non-tracking litter, avoiding stress) and the use of **quarantine and testing prior to introducing cats or kittens into households**. The commercially available **genetic PCR tests** that purport to detect cats that are resistant to FIP are **currently not recommended** as a basis for breeding decisions as they are not accurate in identifying resistant cats. Stress experienced by FCoV-infected cats (e.g., due to surgery, boarding, adoption) or immunosuppression caused by infections, e.g., FIV or FeLV, can predispose cats to developing FIP, so the **minimisation of stress and immunosuppression are important to prevent the development of FIP in FCoV-infected cats**. The FIP vaccine is not useful in FCoV-infected cats.

## 13. Conclusions

While FIP can present at any age, it presents typically in young cats, and effusions, fever, anorexia, and weight loss are common presenting signs. The sampling of effusions or abnormal tissues (by FNA) for cytology and FCoV analysis (either RT-qPCR for FCoV RNA load and/or immunostaining for FCoV antigen) can aid diagnosis. Definitive diagnosis depends on histopathological changes in affected tissues containing FCoV antigen within macrophages detected by immunostaining. Antiviral compounds, especially nucleoside analogues such as oral GS-441524, although not yet licensed for FIP treatment, are now available and are very effective curative treatments. However, treatment is often costly. Trial treatments of cases without a definitive diagnosis of FIP, but in which a diagnosis is very likely, might be warranted, as the response to effective antivirals is usually rapid; this can provide a diagnostic treatment trial. Without effective antiviral treatment, FIP has a very poor prognosis.

These guidelines will continue to be updated regularly on the ABCD website FIP section (www.abcdcatsvets.org [444]) as new data become available. A previous version of the ABCD guidelines was published in 2009 [46]; the current guidelines are a major update of the previous version, reviewing the large body of research published in the field to give a comprehensive review with summary information.

**Summary of Section 13: Conclusions** FIP typically occurs in young cats, and effusions, fever, anorexia, and weight loss are common presenting signs. The sampling of effusions or abnormal tissues by fine-needle aspirates for cytology and FCoV analysis (either RT-qPCR for FCoV RNA load and/or immunostaining for FCoV antigen) can aid diagnosis. Antiviral compounds, especially nucleoside analogues such as oral GS-441524, are effective curative treatments, although treatment is often costly. Trial treatment of cases might be warranted if a diagnosis is very likely, as response to effective antivirals is usually rapid. Without effective antiviral treatment, FIP has a very poor prognosis. Guidelines will continue to be updated regularly on the ABCD website FIP section (www.abcdcatsvets.org [441]) as new data become available.

## Figures and Tables

**Table 1:** Prevalence of FCoV in various countries from selected studies using either serum FCoV antibody or faecal FCoV RNA detection.

Country	Method Used for Prevalence Determination *	Number of Cats	Prevalence	Year of Study [Reference]
Australia	Antibodies	49 feral cats 306 owned cats	0% 34%	2006 [144]
Australia	FCoV RNA in faeces	289 cats with diarrhoea including: 80 shelter cats with diarrhoea	40% 54%	2019 [151]
Austria	Antibodies	159 cats without FIP	71%	2001 [152]
Croatia	Antibodies	106 pet cats	42%	2021 [153]
Czech Republic	FCoV RNA in faeces	70 shelter cats	63%	2022 [129]
Falkland Islands	Antibodies	10 feral cats 95 pet cats	0% 0%	2012 [140]
France	FCoV RNA in faeces	88 healthy cats	17%	2013 [63]
Galapagos Islands	Antibodies	34 pet and 18 feral cats	0%	2008 [139]
Germany	FCoV RNA in faeces	82 cats from 19 breeding catteries	71%	2020 [135]
Germany	Antibodies	82 cats from 19 breeding catteries	78%	2020 [135]
Germany	FCoV RNA in faeces	179 cats from 37 breeding catteries	77% †	2020 [123]
Germany	FCoV RNA in faeces	12 cats in contact with FIP cats 18 cats with FIP	66% 33% ‡	2022 [94]
Germany	FCoV RNA in faeces	All pedigree breeding catteries: 211 cats without diarrhoea 23 cats with diarrhoea 234 total cats	59% 87% 62%	2022 [154]
Greece	Antibodies ¥	267 client-owned cats 156 stray cats 21 cattery cats	10% 15% 19%	2023 [148]
Iran	Antibodies	248 pet cats presenting to a referral hospital	7%	2012 [155]
Israel	Antibodies	68 feral cats 54 shelter cats 33 pet cats	60% 83% 21%	1999 [156]
Italy	Antibodies	24 healthy pet cats 11 FCoV exposed cats 32 cats with FIP	25% 36% 91%	2004 [157]
Italy	Antibodies	120 cattery or shelter cats	82%	2008 [158]
Italy	Antibodies	81 stray colony cats 60 shelter cats 77 owned cats	19% 30% 51%	2022 [147]
Japan	Antibodies	2815 pedigree cats 14,577 non-pedigree domestic shorthair cats	67% 31%	2012 [146]
Republic of Korea	Antibodies	212 (107 pet and 105 feral cats), both sick and healthy in nature	14%	2011 [70]
Republic of Korea	FCoV RNA in faeces	212 (107 pet and 105 feral cats, both sick and healthy in nature)	7%	2011 [70]
Malaysia	Antibodies	24 cats in 4 breeding catteries	100%	2004 [141]
Malaysia	FCoV RNA in faeces	24 cats in a Persian cattery 20 cats in a rescue cattery	96% 70%	2009 [141]
The Netherlands	Antibodies	21 FIP cases 45 in-contact cats 69 cats presented for Non-FIP conditions 109 specific pathogen-free cats	100% 91% 16% 0%	1977 [159]
The Netherlands	FCoV RNA in faeces	17 FIP cats 170 apparently healthy	35% 16%	2010 [112]
Sweden	Antibodies	142 non-pedigree domestic cats 64 pedigree cats	17% 65%	2006 [160]
Taiwan	Antibodies	760 healthy cats 73 cats with FIP	28% 100%	2014 [66]
Turkey	Antibodies	100 healthy cats comprising 79 pet and 21 shelter cats	21%	2009 [161]
Turkey	Antibodies	169 ill cats	37%	2015 [162]
UK	Antibodies	136 of 155 pet cats	88%	2001 [5]
UK	FCoV RNA in faeces	136 cats from 20 multi-cat and 9 single-cat households known to have endemic FCoV	Viral RNA detected in 97% cats at least once	2001 [5]
UK	Antibodies	2207 cats in rescue shelters including: 1173 that were tested within 5 days of admission to the shelter	26% 24%	2004 [143]
UK	Antibodies	131 pedigree cats at cat shows	84%	1992 [163]
UK	Antibodies	516 stray cats	22%	2002 [164]
UK	FCoV RNA in faeces	48 cats with FIP 35 cats without FIP	65% 20%	2017 [106]
UK	FCoV RNA in faeces	1088 cats with diarrhoea 437 pedigree cats 631 domestic cats	57% 79% 42%	2014 [165]
UK	FCoV RNA in faeces	16 cats with FIP 10 cats without FIP	81% 60%	2014 [107]
UK	FCoV RNA in faeces	8 cats with FIP 3 cats without FIP	100% 33%	1996 [166]
USA	FCoV RNA in faeces	50 healthy shelter cats	56%	2018 [83]

* Antibodies indicate serological assay for FCoV antibody detection in blood (serum). The faecal collection method for FCoV RNA detection by reverse-transcriptase polymerase chain reaction (RT-PCR) is not specified, but it is known that faecal samples have higher FCoV loads than rectal swabs [94] the so collection method may influence the prevalence figures obtained. Only studies on faecal samples are included, and an analysis of samples collected directly from the intestines (e.g., at post-mortem examination) is not included. † 77% derived when all four faecal samples that were analysed per cat were included; in contrast, when only the last single, only the last two, or only the last three faecal samples analysed per cat were included, the percentage of FCoV RT-PCR-positive results dropped to 62%, 69%, and 74%, respectively. ‡ 33% represents 6 of 18 cats with FIP that were FCoV RT-PCR-positive in faeces on day 0 before starting treatment for FIP; however, when samples from the first three days of the treatment study were included in the analysis (i.e., days 0–2), 11 of the 18 cats (61%) were faecal FCoV RT-PCR-positive. ¥ In this study, only 15 of the 438 cats for whom the breed was known were pedigree.

**Table 3:** Likelihood of surviving suspected effusive FIP following antiviral treatment with GS-441524 was associated with the serum bilirubin concentration; from Katayama et al. 2021 [19].

Total Serum Bilirubin (µmol/L)	Number of Surviving Cats over Total Number in Category	Survival %
≤8.6	28/29	97
>8.6–17.1	24/27	89
>17.1–34.2	15/20	75
>34.2–68.4	9/18	50
>68.4	1/7	14

**Table 4:** Remdesivir higher dosage treatment protocol used in the study by Coggins et al. 2023 [17].

FIP Presentation	Induction Dosage of Remdesivir	Maintenance Dosage of Remdesivir
Effusive	10 mg/kg q 24 h IV or SC for 4 days	8–10 mg/kg q 24 h SC to 84 days
Non-effusive	15 mg/kg q 24 h IV or SC for 4 days	10–12 mg/kg q 24 h SC to 84 days
Neurological and/or ocular signs present	15 mg/kg q 24 h IV or SC for 4 days	12–15 mg/kg q 24 h SC to 84 days

**Table 5:** Immunomodulatory and supportive treatments that have been used in cats with FIP. SC; subcutaneously, PO; orally, IM; intramuscularly, CRI; constant rate infusion, NSAID; non-steroidal anti-inflammatory drug, rfIFN-ω; recombinant feline IFN-omega.

Drug	Comments	ABCD Recommendation in FIP
Meloxicam	Meloxicam, a NSAID licensed for use in cats, was associated with long-term survival in one cat [224], and in three cats in which it was used alongside rfIFN-ω [43].	Worthy of further studies. In some countries metamizole used in place of NSAIDs. Do not use in dehydration or hypotension and care in renal disease or anorexic cats. Was associated with worsening acute kidney injury in one cat with FIP [45].
Gabapentin	Anxiolytic/analgesic/sedative which can help if SC injections (e.g., remdesivir) are needed (when oral GS-441524 cannot be given) that cause pain. Not licensed.	No prospective studies in cats with FIP but has been used successfully [17,332]. Adverse effects can include sedation and ataxia. Typically give 50 or 100 mgs (can be up to 200 mg if required but start at low dose initially) per cat PO [408] around two hours before SC injection.
Mirtazapine	Appetite stimulant/anti-nausea. For prevention and treatment of vomiting and nausea and as appetite stimulant; can be given as a trial if nausea suspected.	No published studies in cats with FIP. Has been used in anorexic cats before and during treatment [43,45]. Please note that efficacious antiviral treatment e.g., GS-441524 usually causes a rapid return of appetite. Given at 2 mg/cat PO or as transdermal q 24 h (q 48 h if renal/hepatic involvement of FIP) [409].
Maropitant	For prevention and treatment of vomiting and nausea. SC injection can be painful.	No published evaluation studies in cats with FIP, although used as supportive treatment [24,45]; use if indicated. Dosage is 1 mg/kg SC, IV or PO q 24 h [409].
Metoclopramide	Prevention and treatment of nausea and vomiting, and management of ileus and delayed gastric emptying.	No published evaluation studies in cats with FIP but use if indicated. Dosage 0.25–0.5 mg/kg IV, IM, SC or PO q 8h or 1–2 mg/kg IV over 24 h as a CRI; CRI can be more effective than bolus dosing [409].
Ondansetron	For prevention and treatment of vomiting and nausea refractory to other agents such as maropitant, mirtazapine and metoclopramide. Expensive. Injectable or oral formulations available.	No published evaluation studies in cats with FIP, although used as supportive treatment [45]; use if indicated. Dosage is 0.1–1 mg/kg IV (slowly), IM, SC, or PO q 6–12 h [409].
Hepatoprotectants such as S-Adenosylmethionine (SAMe)	Various preparations exist. Sometimes used during antiviral (especially GS-441524) treatment of FIP if hepatocellular enzymes (ALT) become elevated, or when these are normal by some [43], as protection against hepatic damage; however, ALT normalisation usually occurs rapidly during, or following cessation, of antiviral treatment without the use of hepatoprotectants, so their need is not proven.	No published evaluation studies in FIP but has been used during treatment [24,43] without problems. This is an option if clinical concerns exist regarding hepatotoxicity. Might not be needed
Prednisolone/ dexamethasone	Acts as anti-inflammatory or immunosuppressant depending on dosage used. No controlled studies available. Median survival time of FIP cats treated with prednisolone was only eight days [37]. Does not cure FIP. Cats treated with systemic glucocorticoids along with polyprenyl immunostimulant (PI) had a shorter survival than those treated with PI alone [330].	Not recommended although can be used as palliative treatment and topical glucocorticoid treatment can be used for the treatment of ocular FIP with uveitis if needed. Suggested that glucocorticoid treatment is associated with a poorer FIP outcome when used concurrently with other treatments (e.g., such as rfIFN-ω [43], polyprenyl immunostimulant [330]).
Polyprenyl immunostimulant (PI)	Shows promise in the treatment of FIP without effusions, especially in cats with haematocrit and/or A:G ratios that are normal or that increase with treatment [212]. Takes a long time for response; reported normalisation times are ~182 days for haematocrit and ~375 days for the A:G ratio [212]. Reversal of lymphopenia with treatment [212].	Do not use with systemic glucocorticoids [330] but topical glucocorticoid treatment can be used with PI in ocular FIP uveitis [330]. Dosage 3 mg/kg PO three times a week or q 48 h [43,212,330,410]. Some cats are changed to a maintenance dosage of 3 mg/kg PO once or twice a week after one year of treatment [212].
Pentoxyfylline/ propentofylline	Aim at treating vasculitis. One placebo-controlled double-blind study on propentofylline showed no efficacy (but all cats were also given glucocorticoids) [411]	Controlled field studies without glucocorticoids required.
Anti-TNF-α antibody	Blocks TNF-α that is involved in exacerbating clinical signs of FIP [186,187,188]. Some efficacy in a placebo-controlled study including a few cats (three treated, three placebo) with experimentally induced FIP [412]. Used in an uncontrolled very small study of cats with experimentally induced FIP alongside itraconazole treatment [190] in which two of three cats with FIP improved.	Controlled field studies required.
Azathioprine	Aims to immunosuppress (and to lower the prednisolone/dexamethasone dose). No published studies available.	Not recommended due to toxicity in cats.
Chlorambucil	Aims to immunosuppress (and to lower the prednisolone/dexamethasone dose). No published studies.	Not recommended.
Cyclophosphamide	Aims to immunosuppress (and to lower the prednisolone/ dexamethasone dose). No published studies.	Not recommended.
Ozagrel hydrochloride	Inhibits thromboxane synthesis leading to reduced platelet aggregation and cytokine release. Used in two cats with some improvement of clinical signs [413] but unsuccessful in other cases [387].	Not recommended.

## References

[1] Pedersen N.C., Boyle J.F., Floyd K., Fudge A., Barker J. (1981). An enteric coronavirus infection of cats and its relationship to feline infectious peritonitis. Am. J. Vet. Res..

[2] Hayashi T., Watabe Y., Nakayama H., Fujiwara K. (1982). Enteritis due to feline infectious peritonitis virus. Jpn. J. Vet. Res..

[3] Addie D.D., Jarrett O. (1992). A study of naturally occurring feline coronavirus infections in kittens. Vet. Rec..

[4] Kipar A., Kremendahl J., Addie D.D., Leukert W., Grant C.K., Reinacher M. (1998). Fatal enteritis associated with coronavirus infection in cats. J. Comp. Pathol..

[5] Addie D.D., Jarrett O. (2001). Use of a reverse-transcriptase polymerase chain reaction for monitoring the shedding of feline coronavirus by healthy cats. Vet. Rec..

[6] Kipar A., May H., Menger S., Weber M., Leukert W., Reinacher M. (2005). Morphologic features and development of granulomatous vasculitis in feline infectious peritonitis. Vet. Pathol..

[7] Kipar A., Meli M.L., Baptiste K.E., Bowker L.J., Lutz H. (2010). Sites of feline coronavirus persistence in healthy cats. J. Gen. Virol..

[8] Mustaffa-Kamal F., Liu H., Pedersen N.C., Sparger E.E. (2019). Characterization of antiviral T cell responses during primary and secondary challenge of laboratory cats with feline infectious peritonitis virus (FIPV). BMC Vet. Res..

[9] Pedersen N.C. (2014). An update on feline infectious peritonitis: Virology and immunopathogenesis. Vet. J..

[10] Zehr J.D., Kosakovsky Pond S.L., Millet J.K., Olarte-Castillo X.A., Lucaci A.G., Shank S.D., Ceres K.M., Choi A., Whittaker G.R., Goodman L.B. (2023). Natural selection differences detected in key protein domains between non-pathogenic and pathogenic feline coronavirus phenotypes. Virus Evol..

[11] Pesteanu-Somogyi L.D., Radzai C., Pressler B.M. (2006). Prevalence of feline infectious peritonitis in specific cat breeds. J. Feline Med. Surg..

[12] Robison R.L., Holzworth J., Gilmore C.E. (1971). Naturally occurring feline infectious peritonitis: Signs and clinical diagnosis. J. Am. Vet. Med. Assoc..

[13] Rohrbach B.W., Legendre A.M., Baldwin C.A., Lein D.H., Reed W.M., Wilson R.B. (2001). Epidemiology of feline infectious peritonitis among cats examined at veterinary medical teaching hospitals. J. Am. Vet. Med. Assoc..

[14] Worthing K.A., Wigney D.I., Dhand N.K., Fawcett A., McDonagh P., Malik R., Norris J.M. (2012). Risk factors for feline infectious peritonitis in Australian cats. J. Feline Med. Surg..

[15] Norris J.M., Bosward K.L., White J.D., Baral R.M., Catt M.J., Malik R. (2005). Clinicopathological findings associated with feline infectious peritonitis in Sydney, Australia: 42 cases (1990–2002). Aust. Vet. J..

[16] Soma T., Wada M., Taharaguchi S., Tajima T. (2013). Detection of ascitic feline coronavirus RNA from cats with clinically suspected feline infectious peritonitis. J. Vet. Med. Sci..

[17] Coggins S.J., Norris J.M., Malik R., Govendir M., Hall E.J., Kimble B., Thompson M.F. (2023). Outcomes of treatment of cats with feline infectious peritonitis using parenteral remdesivir, with or without transition to oral GS-441524. J. Vet. Intern. Med..

[18] Riemer F., Kuehner K.A., Ritz S., Sauter-Louis C., Hartmann K. (2016). Clinical and laboratory features of cats with feline infectious peritonitis—A retrospective study of 231 confirmed cases (2000–2010). J. Feline Med. Surg..

[19] Katayama M., Uemura Y. (2021). Therapeutic Effects of Mutian(^®^) Xraphconn on 141 Client-Owned Cats with Feline Infectious Peritonitis Predicted by Total Bilirubin Levels. Vet. Sci..

[20] Yin Y., Li T., Wang C., Liu X., Ouyang H., Ji W., Liu J., Liao X., Li J., Hu C. (2021). A retrospective study of clinical and laboratory features and treatment on cats highly suspected of feline infectious peritonitis in Wuhan, China. Sci. Rep..

[21] Pedersen N.C. (2009). A review of feline infectious peritonitis virus infection: 1963–2008. J. Feline Med. Surg..

[22] Felten S., Hartmann K. (2019). Diagnosis of Feline Infectious Peritonitis: A Review of the Current Literature. Viruses.

[23] Jones S., Novicoff W., Nadeau J., Evans S. (2021). Unlicensed GS-441524-Like Antiviral Therapy Can Be Effective for at-Home Treatment of Feline Infectious Peritonitis. Animals.

[24] Krentz D., Zenger K., Alberer M., Felten S., Bergmann M., Dorsch R., Matiasek K., Kolberg L., Hofmann-Lehmann R., Meli M.L. (2021). Curing Cats with Feline Infectious Peritonitis with an Oral Multi-Component Drug Containing GS-441524. Viruses.

[25] Sweet A.N., Andre N.M., Stout A.E., Licitra B.N., Whittaker G.R. (2022). Clinical and Molecular Relationships between COVID-19 and Feline Infectious Peritonitis (FIP). Viruses.

[26] Sparkes A.H., Gruffydd-Jones T.J., Harbour D.A. (1991). Feline infectious peritonitis: A review of clinicopathological changes in 65 cases, and a critical assessment of their diagnostic value. Vet. Rec..

[27] Tsai H.Y., Chueh L.L., Lin C.N., Su B.L. (2011). Clinicopathological findings and disease staging of feline infectious peritonitis: 51 cases from 2003 to 2009 in Taiwan. J. Feline Med. Surg..

[28] Baek S., Jo J., Song K., Seo K. (2017). Recurrent pericardial effusion with feline infectious peritonitis in a cat. J. Vet. Clin..

[29] Lewis K.M., O’Brien R.T. (2010). Abdominal Ultrasonographic Findings Associated With Feline Infectious Peritonitis: A Retrospective Review of 16 Cases. J. Am. Anim. Hosp. Assoc..

[30] Dunbar D., Kwok W., Graham E., Armitage A., Irvine R., Johnston P., McDonald M., Montgomery D., Nicolson L., Robertson E. (2019). Diagnosis of non-effusive feline infectious peritonitis by reverse transcriptase quantitative PCR from mesenteric lymph node fine-needle aspirates. J. Feline Med. Surg..

[31] Katayama M., Uemura Y. (2023). Prognostic Prediction for Therapeutic Effects of Mutian on 324 Client-Owned Cats with Feline Infectious Peritonitis Based on Clinical Laboratory Indicators and Physical Signs. Vet. Sci..

[32] Ziolkowska N., Pazdzior-Czapula K., Lewczuk B., Mikulska-Skupien E., Przybylska-Gornowicz B., Kwiecinska K., Ziolkowski H. (2017). Feline Infectious Peritonitis: Immunohistochemical Features of Ocular Inflammation and the Distribution of Viral Antigens in Structures of the Eye. Vet. Pathol..

[33] Crawford A.H., Stoll A.L., Sanchez-Masian D., Shea A., Michaels J., Fraser A.R., Beltran E. (2017). Clinicopathologic Features and Magnetic Resonance Imaging Findings in 24 Cats With Histopathologically Confirmed Neurologic Feline Infectious Peritonitis. J. Vet. Intern. Med..

[34] Kline K.L., Joseph R.J., Averdill D.R. (1994). Feline infectious peritonitis with neurologic involvement: Clinical and pathological findings in 24 cats. J. Am. Anim. Hosp. Assoc..

[35] Foley J.E., Lapointe J.M., Koblik P., Poland A., Pedersen N.C. (1998). Diagnostic features of clinical neurologic feline infectious peritonitis. J. Vet. Intern. Med..

[36] Thayer V., Gogolski S., Felten S., Hartmann K., Kennedy M., Olah G.A. (2022). 2022 AAFP/EveryCat Feline Infectious Peritonitis Diagnosis Guidelines. J. Feline Med. Surg..

[37] Ritz S., Egberink H., Hartmann K. (2007). Effect of feline interferon-omega on the survival time and quality of life of cats with feline infectious peritonitis. J. Vet. Intern. Med..

[38] Pedersen N.C., Perron M., Bannasch M., Montgomery E., Murakami E., Liepnieks M., Liu H. (2019). Efficacy and safety of the nucleoside analog GS-441524 for treatment of cats with naturally occurring feline infectious peritonitis. J. Feline Med. Surg..

[39] Addie D.D., Covell-Ritchie J., Jarrett O., Fosbery M. (2020). Rapid Resolution of Non-Effusive Feline Infectious Peritonitis Uveitis with an Oral Adenosine Nucleoside Analogue and Feline Interferon Omega. Viruses.

[40] Dickinson P.J., Bannasch M., Thomasy S.M., Murthy V.D., Vernau K.M., Liepnieks M., Montgomery E., Knickelbein K.E., Murphy B., Pedersen N.C. (2020). Antiviral treatment using the adenosine nucleoside analogue GS-441524 in cats with clinically diagnosed neurological feline infectious peritonitis. J. Vet. Intern. Med..

[41] Bohm M. (2022). Successful treatment of a South African cat with effusive feline infectious peritonitis with remdesivir. J. S. Afr. Vet. Assoc..

[42] Nekouei O., St-Hilaire S., Hui P.C., Chan K., Chan I.S., Ngan S.Y.L., Chan Y., Chung K.P., Hong S., Chan H.M. (2022). Potential therapeutic effects of GS-441524 and GC376 in cats with feline infectious peritonitis. Vet. Evid..

[43] Addie D.D., Silveira C., Aston C., Brauckmann P., Covell-Ritchie J., Felstead C., Fosbery M., Gibbins C., Macaulay K., McMurrough J. (2022). Alpha-1 Acid Glycoprotein Reduction Differentiated Recovery from Remission in a Small Cohort of Cats Treated for Feline Infectious Peritonitis. Viruses.

[44] Roy M., Jacque N., Novicoff W., Li E., Negash R., Evans S.J.M. (2022). Unlicensed Molnupiravir is an Effective Rescue Treatment Following Failure of Unlicensed GS-441524-like Therapy for Cats with Suspected Feline Infectious Peritonitis. Pathogens.

[45] Green J., Syme H., Tayler S. (2023). Thirty-two cats with effusive or non-effusive feline infectious peritonitis treated with a combination of remdesivir and GS-441524. J. Vet. Intern. Med..

[46] Addie D., Belak S., Boucraut-Baralon C., Egberink H., Frymus T., Gruffydd-Jones T., Hartmann K., Hosie M.J., Lloret A., Lutz H. (2009). Feline infectious peritonitis. ABCD guidelines on prevention and management. J. Feline Med. Surg..

[47] Owens R.A., Flores R., Di Serio F., Li S.F., Pallás V., Randles J.W., Sano T., Vidalakis G., King A.M.Q., Adams M.J., Carstens E.B., Lefkowitz E.J. (2012). Viroids. Ninth Report of the International Committee on Taxonomy of Viruses.

[48] De Groot R.J., Baker S.C., Baric R.S., Enjuanes L., Gorbalenya A.E., Holmes K.V., Perlman S., Poon L., Rottier P.J.M., Talbot P.J., King A.M.Q., Adams M.J., Carstens E.B., Lefkowitz E.J. (2012). Family Coronaviridae. Ninth Report International Committee on Taxonomy of Viruses.

[49] Jaimes J.A., Millet J.K., Stout A.E., Andre N.M., Whittaker G.R. (2020). A Tale of Two Viruses: The Distinct Spike Glycoproteins of Feline Coronaviruses. Viruses.

[50] Haake C., Cook S., Pusterla N., Murphy B. (2020). Coronavirus Infections in Companion Animals: Virology, Epidemiology, Clinical and Pathologic Features. Viruses.

[51] Hosie M.J., Hofmann-Lehmann R., Hartmann K., Egberink H., Truyen U., Addie D.D., Belak S., Boucraut-Baralon C., Frymus T., Lloret A. (2021). Anthropogenic Infection of Cats during the 2020 COVID-19 Pandemic. Viruses.

[52] Addie D.D., Boucraut-Baralon C., Egberink H., Frymus T., Gruffydd-Jones T., Hartmann K., Horzinek M.C., Hosie M.J., Lloret A., Lutz H. (2015). Disinfectant choices in veterinary practices, shelters and households: ABCD guidelines on safe and effective disinfection for feline environments. J. Feline Med. Surg..

[53] Scott F.W. Update on FIP. Proceedings of the 12th Annual Kal Kan Symposium for the Treatment of Small Animal Diseases.

[54] Barker E.N., Tasker S. (2020). Advances in Molecular Diagnostics and Treatment of Feline Infectious Peritonitis. Adv. Small Anim. Care.

[55] Healey E.A., Andre N.M., Miller A.D., Whitaker G.R., Berliner E.A. (2022). Outbreak of feline infectious peritonitis (FIP) in shelter-housed cats: Molecular analysis of the feline coronavirus S1/S2 cleavage site consistent with a ‘circulating virulent–avirulent theory’ of FIP pathogenesis. J. Feline Med. Surg. Open Rep..

[56] Millet J.K., Whittaker G.R. (2015). Host cell proteases: Critical determinants of coronavirus tropism and pathogenesis. Virus Res..

[57] Shi W., Cai Y., Zhu H., Peng H., Voyer J., Rits-Volloch S., Cao H., Mayer M.L., Song K., Xu C. (2023). Cryo-EM structure of SARS-CoV-2 postfusion spike in membrane. Nature.

[58] Terada Y., Matsui N., Noguchi K., Kuwata R., Shimoda H., Soma T., Mochizuki M., Maeda K. (2014). Emergence of pathogenic coronaviruses in cats by homologous recombination between feline and canine coronaviruses. PLoS ONE.

[59] Horzinek M.C., Lutz H. (2000). An Update on Feline Infectious Peritonitis. Vet. Sci. Tomorrow.

[60] Jaimes J.A., Whittaker G.R. (2018). Feline coronavirus: Insights into viral pathogenesis based on the spike protein structure and function. Virology.

[61] Shiba N., Maeda K., Kato H., Mochizuki M., Iwata H. (2007). Differentiation of feline coronavirus type I and II infections by virus neutralization test. Vet. Microbiol..

[62] Herrewegh A.A., Smeenk I., Horzinek M.C., Rottier P.J., de Groot R.J. (1998). Feline coronavirus type II strains 79-1683 and 79-1146 originate from a double recombination between feline coronavirus type I and canine coronavirus. J. Virol..

[63] Le Poder S., Pham-Hung d’Alexandry d’Orangiani A.L., Duarte L., Fournier A., Horhogea C., Pinhas C., Vabret A., Eloit M. (2013). Infection of cats with atypical feline coronaviruses harbouring a truncated form of the canine type I non-structural ORF3 gene. Infect. Genet. Evol..

[64] Addie D.D., Schaap I.A.T., Nicolson L., Jarrett O. (2003). Persistence and transmission of natural type I feline coronavirus infection. J. Gen. Virol..

[65] Lin C.N., Su B.L., Wang C.H., Hsieh M.W., Chueh T.J., Chueh L.L. (2009). Genetic diversity and correlation with feline infectious peritonitis of feline coronavirus type I and II: A 5-year study in Taiwan. Vet. Microbiol..

[66] Wang Y.T., Chueh L.L., Wan C.H. (2014). An eight-year epidemiologic study based on baculovirus-expressed type-specific spike proteins for the differentiation of type I and II feline coronavirus infections. BMC Vet. Res..

[67] Decaro N., Mari V., Lanave G., Lorusso E., Lucente M.S., Desario C., Colaianni M.L., Elia G., Ferringo F., Alfano F. (2021). Mutation analysis of the spike protein in Italian feline infectious peritonitis virus and feline enteric coronavirus sequences. Res. Vet. Sci..

[68] Lin L., Yao D., Wu L., Fan R., Liu Y., Zhou Z. (2022). Molecular epidemiology of type I and II feline coronavirus from cats with suspected feline infectious peritonitis in China between 2019 and 2021. Arch. Virol..

[69] Xu L., Ye S., Ding Y., Xiao Y., Yao C., Wang Z., Cai S., Ou J., Mao J., Hu X. (2023). A Combined Method Based on the FIPV N Monoclonal Antibody Immunofluorescence Assay and RT-nPCR Method for the Rapid Diagnosis of FIP-Suspected Ascites. Transbound. Emerg. Dis..

[70] An D.J., Jeoung H.Y., Jeong W., Park J.Y., Lee M.H., Park B.K. (2011). Prevalence of Korean cats with natural feline coronavirus infections. Virol. J..

[71] Duarte A., Veiga I., Tavares L. (2009). Genetic diversity and phylogenetic analysis of Feline Coronavirus sequences from Portugal. Vet. Microbiol..

[72] Amer A., Siti Suri A., Abdul Rahman O., Mohd H.B., Faruku B., Saeed S., Tengku Azmi T.I. (2012). Isolation and molecular characterization of type I and type II feline coronavirus in Malaysia. Virol. J..

[73] Zhou Q., Li Y., Huang J., Fu N., Song X., Sha X., Zhang B. (2021). Prevalence and molecular characteristics of feline coronavirus in southwest China from 2017 to 2020. J. Gen. Virol..

[74] Hohdatsu T., Okada S., Ishizuka Y., Yamada H., Koyama H. (1992). The prevalence of types I and II feline coronavirus infections in cats. J. Vet. Med. Sci..

[75] Kummrow M., Meli M.L., Haessig M., Goenczi E., Poland A., Pedersen N.C., Hofmann-Lehmann R., Lutz H. (2005). Feline coronavirus serotypes 1 and 2: Seroprevalence and association with disease in Switzerland. Clin. Diagn. Lab. Immunol..

[76] Benetka V., Kubber-Heiss A., Kolodziejek J., Nowotny N., Hofmann-Parisot M., Mostl K. (2004). Prevalence of feline coronavirus types I and II in cats with histopathologically verified feline infectious peritonitis. Vet. Microbiol..

[77] Pedersen N.C., Evermann J.F., McKeirnan A.J., Ott R.L. (1984). Pathogenicity studies of feline coronavirus isolates 79-1146 and 79-1683. Am. J. Vet. Res..

[78] Desmarets L.M., Theuns S., Olyslaegers D.A., Dedeurwaerder A., Vermeulen B.L., Roukaerts I.D., Nauwynck H.J. (2013). Establishment of feline intestinal epithelial cell cultures for the propagation and study of feline enteric coronaviruses. Vet. Res..

[79] Tekes G., Ehmann R., Boulant S., Stanifer M.L. (2020). Development of Feline Ileum- and Colon-Derived Organoids and Their Potential Use to Support Feline Coronavirus Infection. Cells.

[80] Lai M.M., Cavanagh D. (1997). The molecular biology of coronaviruses. Adv. Virus Res..

[81] Poland A.M., Vennema H., Foley J.E., Pedersen N.C. (1996). Two related strains of feline infectious peritonitis virus isolated from immunocompromised cats infected with a feline enteric coronavirus. J. Clin. Microbiol..

[82] Gao Y.Y., Wang Q., Liang X.Y., Zhang S., Bao D., Zhao H., Li S.B., Wang K., Hu G.X., Gao F.S. (2023). An updated review of feline coronavirus: Mind the two biotypes. Virus Res..

[83] Fish E.J., Diniz P.P.V., Juan Y.C., Bossong F., Collisson E.W., Drechsler Y., Kaltenboeck B. (2018). Cross-sectional quantitative RT-PCR study of feline coronavirus viremia and replication in peripheral blood of healthy shelter cats in Southern California. J. Feline Med. Surg..

[84] Kipar A., Baptiste K., Barth A., Reinacher M. (2006). Natural FCoV infection: Cats with FIP exhibit significantly higher viral loads than healthy infected cats. J. Feline Med. Surg..

[85] Meli M., Kipar A., Muller C., Jenal K., Gonczi E., Borel N., Gunn-Moore D., Chalmers S., Lin F., Reinacher M. (2004). High viral loads despite absence of clinical and pathological findings in cats experimentally infected with feline coronavirus (FCoV) type I and in naturally infected FCoV-infected cats. J. Feline Med. Surg..

[86] Borschensky C.M., Reinacher M. (2014). Mutations in the 3c and 7b genes of feline coronavirus in spontaneously affected FIP cats. Res. Vet. Sci..

[87] Bank-Wolf B.R., Stallkamp I., Wiese S., Moritz A., Tekes G., Thiel H.J. (2014). Mutations of 3c and spike protein genes correlate with the occurrence of feline infectious peritonitis. Vet. Microbiol..

[88] Xia H., Li X., Zhao W., Jia S., Zhang X., Irwin D.M., Zhang S. (2020). Adaptive Evolution of Feline Coronavirus Genes Based on Selection Analysis. BioMed Res. Int..

[89] Paltrinieri S., Giordano A., Stranieri A., Lauzi S. (2021). Feline infectious peritonitis (FIP) and coronavirus disease 19 (COVID-19): Are they similar?. Transbound. Emerg. Dis..

[90] Barker E.N., Tasker S., Gruffydd-Jones T.J., Tuplin C.K., Burton K., Porter E., Day M.J., Harley R., Fews D., Helps C.R. (2013). Phylogenetic analysis of feline coronavirus strains in an epizootic outbreak of feline infectious peritonitis. J. Vet. Intern. Med..

[91] Chang H.W., Egberink H.F., Halpin R., Spiro D.J., Rottier P.J. (2012). Spike protein fusion Peptide and feline coronavirus virulence. Emerg. Infect. Dis..

[92] Pedersen N.C., Liu H., Dodd K.A., Pesavento P.A. (2009). Significance of Coronavirus Mutants in Feces and Diseased Tissues of Cats Suffering from Feline Infectious Peritonitis. Viruses.

[93] Chang H.W., Egberink H.F., Rottier P.J. (2011). Sequence analysis of feline coronaviruses and the circulating virulent/avirulent theory. Emerg. Infect. Dis..

[94] Meli M.L., Spiri A.M., Zwicklbauer K., Krentz D., Felten S., Bergmann M., Dorsch R., Matiasek K., Alberer M., Kolberg L. (2022). Fecal Feline Coronavirus RNA Shedding and Spike Gene Mutations in Cats with Feline Infectious Peritonitis Treated with GS-441524. Viruses.

[95] Brown M.A., Troyer J.L., Pecon-Slattery J., Roelke M.E., O’Brien S.J. (2009). Genetics and pathogenesis of feline infectious peritonitis virus. Emerg. Infect. Dis..

[96] Wang Y.T., Su B.L., Hsieh L.E., Chueh L.L. (2013). An outbreak of feline infectious peritonitis in a Taiwanese shelter: Epidemiologic and molecular evidence for horizontal transmission of a novel type II feline coronavirus. Vet. Res..

[97] Graham E.M., Went K., Serra F., Dunbar D., Fuentes M., Mcdonald M., Jackson M.W. (2012). Early molecular diagnosis of an effusive FIP outbreak in antibody-negative kittens. J. Feline Med. Surg..

[98] Attipa C., Gunn-Moore D., Mazeri S., Epaminondas D., Lyraki M., Hardas A., Loukaidou S., Gentil M. (2023). Concerning feline infectious peritonitis outbreak in Cyprus. Vet. Rec..

[99] Pedersen N.C., Liu H., Scarlett J., Leutenegger C.M., Golovko L., Kennedy H., Kamal F.M. (2012). Feline infectious peritonitis: Role of the feline coronavirus 3c gene in intestinal tropism and pathogenicity based upon isolates from resident and adopted shelter cats. Virus Res..

[100] Li F. (2016). Structure, Function, and Evolution of Coronavirus Spike Proteins. Annu. Rev. Virol..

[101] Bosch B.J., van der Zee R., de Haan C.A., Rottier P.J. (2003). The coronavirus spike protein is a class I virus fusion protein: Structural and functional characterization of the fusion core complex. J. Virol..

[102] Tekes G., Hofmann-Lehmann R., Bank-Wolf B., Maier R., Thiel H.J., Thiel V. (2010). Chimeric feline coronaviruses that encode type II spike protein on type I genetic background display accelerated viral growth and altered receptor usage. J. Virol..

[103] Tusell S.M., Schittone S.A., Holmes K.V. (2007). Mutational analysis of aminopeptidase N, a receptor for several group 1 coronaviruses, identifies key determinants of viral host range. J. Virol..

[104] Dye C., Temperton N., Siddell S.G. (2007). Type I feline coronavirus spike glycoprotein fails to recognize aminopeptidase N as a functional receptor on feline cell lines. J. Gen. Virol..

[105] Belouzard S., Millet J.K., Licitra B.N., Whittaker G.R. (2012). Mechanisms of coronavirus cell entry mediated by the viral spike protein. Viruses.

[106] Barker E.N., Stranieri A., Helps C.R., Porter E., Davidson A.D., Day M.J., Kipar A., Tasker S. (2017). Limitations of using feline coronavirus spike protein gene mutations to diagnose feline infectious peritonitis. Vet. Res..

[107] Porter E., Tasker S., Day M.J., Harley R., Kipar A., Siddell S.G., Helps C.R. (2014). Amino acid changes in the spike protein of feline coronavirus correlate with systemic spread of virus from the intestine and not with feline infectious peritonitis. Vet. Res..

[108] Licitra B.N., Millet J.K., Regan A.D., Hamilton B.S., Rinaldi V.D., Duhamel G.E., Whittaker G.R. (2013). Mutation in spike protein cleavage site and pathogenesis of feline coronavirus. Emerg. Infect. Dis..

[109] Andre N.M., Cossic B., Davies E., Miller A.D., Whittaker G.R. (2019). Distinct mutation in the feline coronavirus spike protein cleavage activation site in a cat with feline infectious peritonitis-associated meningoencephalomyelitis. J. Feline Med. Surg. Open Rep..

[110] Ouyang H., Liu J., Yin Y., Cao S., Yan R., Ren Y., Zhou D., Li Q., Li J., Liao X. (2022). Epidemiology and Comparative Analyses of the S Gene on Feline Coronavirus in Central China. Pathogens.

[111] Lewis C.S., Porter E., Matthews D., Kipar A., Tasker S., Helps C.R., SiddelL S.G. (2015). Genotyping coronaviruses associated with feline infectious peritonitis. J. Gen. Virol..

[112] Chang H.W., de Groot R.J., Egberink H.F., Rottier P.J. (2010). Feline infectious peritonitis: Insights into feline coronavirus pathobiogenesis and epidemiology based on genetic analysis of the viral 3c gene. J. Gen. Virol..

[113] Hsieh L.E., Huang W.P., Tang D.J., Wang Y.T., Chen C.T., Chueh L.L. (2013). 3C protein of feline coronavirus inhibits viral replication independently of the autophagy pathway. Res. Vet. Sci..

[114] Vennema H., Rossen J.W., Wesseling J., Horzinek M.C., Rottier P.J. (1992). Genomic organization and expression of the 3’ end of the canine and feline enteric coronaviruses. Virology.

[115] Myrrha L.W., Silva F.M.F., Vidigal P.M.P., Resende M., Bressan G.C., Fietto J.L.R., Santos M.R., Silva L.M.N., Assao V.S., Silva-Ju Nior A. (2019). Feline coronavirus isolates from a part of Brazil: Insights into molecular epidemiology and phylogeny inferred from the 7b gene. J. Vet. Med. Sci..

[116] Herrewegh A.A., Vennema H., Horzinek M.C., Rottier P.J., de Groot R.J. (1995). The molecular genetics of feline coronaviruses: Comparative sequence analysis of the ORF7a/7b transcription unit of different biotypes. Virology.

[117] Andre N.M., Miller A.D., Whittaker G.R. (2020). Feline infectious peritonitis virus-associated rhinitis in a cat. J. Feline Med. Surg. Open Rep..

[118] Pastoret P.P., Henroteaux M. (1978). Epigenetic transmission of feline infectious peritonitis. Comp. Immunol. Microbiol. Infect. Dis..

[119] Addie D.D., Jarrett O. (1990). Control of feline coronavirus infection in kittens. Vet. Rec..

[120] Stranieri A., Probo M., Pisu M.C., Fioletti A., Meazzi S., Gelain M.E., Bonsembiante F., Lauzi S., Paltrinieri S. (2020). Preliminary investigation on feline coronavirus presence in the reproductive tract of the tom cat as a potential route of viral transmission. J. Feline Med. Surg..

[121] Villar M., Fernandez de Mera I.G., Artigas-Jeronimo S., Contreras M., Gortazar C., de la Fuente J. (2020). Coronavirus in cat flea: Findings and questions regarding COVID-19. Parasit Vectors.

[122] Lutz H., Gut M., Leutenegger C.M., Schiller I., Wiseman A., Meli M. Kinetics of FCoV infection in kittens born in catteries of high risk for FIP under different rearing conditions. Proceedings of the Second International Feline Coronavirus/Feline Infectious Peritonitis Symposium.

[123] Klein-Richers U., Hartmann K., Hofmann-Lehmann R., Unterer S., Bergmann M., Rieger A., Leutenegger C., Pantchev N., Balzer J., Felten S. (2020). Prevalence of Feline Coronavirus Shedding in German Catteries and Associated Risk Factors. Viruses.

[124] Felten S., Klein-Richers U., Unterer S., Bergmann M., Zablotski Y., Hofmann-Lehmann R., Hartmann K. (2023). Patterns of Feline Coronavirus Shedding and Associated Factors in Cats from Breeding Catteries. Viruses.

[125] Stoddart M.E., Gaskell R.M., Harbour D.A., Gaskell C.J. (1988). Virus shedding and immune responses in cats inoculated with cell culture-adapted feline infectious peritonitis virus. Vet. Microbiol..

[126] Pedersen N.C., Allen C.E., Lyons L.A. (2008). Pathogenesis of feline enteric coronavirus infection. J. Feline Med. Surg..

[127] Herrewegh A.A., Mahler M., Hedrich H.J., Haagmans B.L., Egberink H.F., Horzinek M.C., Rottier P.J., de Groot R.J. (1997). Persistence and evolution of feline coronavirus in a closed cat-breeding colony. Virology.

[128] Foley J.E., Poland A., Carlson J., Pedersen N.C. (1997). Patterns of feline coronavirus infection and fecal shedding from cats in multiple-cat environments. J. Am. Vet. Med. Assoc..

[129] Vojtkovska V., Lukesova G., Voslarova E., Konvalinova J., Vecerek V., Lobova D. (2022). Direct Detection of Feline Coronavirus by Three Rapid Antigen Immunochromatographic Tests and by Real-Time PCR in Cat Shelters. Vet. Sci..

[130] Bubenikova J., Vrabelova J., Stejskalova K., Futas J., Plasil M., Cerna P., Oppelt J., Lobova D., Molinkova D., Horin P. (2020). Candidate Gene Markers Associated with Fecal Shedding of the Feline Enteric Coronavirus (FECV). Pathogens.

[131] Vogel L., Van der Lubben M., Lintelo E.G.T., Bekker C.P.J., Geerts T., Schuijff L.S., Grinwis G.C.M., Egberink H.F., Rottier P.J.M. (2010). Pathogenic characteristics of persistent feline enteric coronavirus infection in cats. Vet. Res..

[132] Addie D.D., Bellini F., Covell-Ritchie J., Crowe B., Curran S., Fosbery M., Hills S., Johnson E., Johnson C., Lloyd S. (2023). Stopping Feline Coronavirus Shedding Prevented Feline Infectious Peritonitis. Viruses.

[133] Rohner M. (1999). Bestimmung der Ausscheidungskinetik von Felinen Coronaviren unter Feldbedingungen.

[134] Addie D.D., le Poder S., Burr P., Decaro N., Graham E., Hofmann-Lehmann R., Jarrett O., McDonald M., Meli M.L. (2015). Utility of feline coronavirus antibody tests. J. Feline Med. Surg..

[135] Felten S., Klein-Richers U., Hofmann-Lehmann R., Bergmann M., Unterer S., Leutenegger C.M., Hartmann K. (2020). Correlation of Feline Coronavirus Shedding in Feces with Coronavirus Antibody Titer. Pathogens.

[136] Kim Y., Liu H., Galasiti Kankanamalage A.C., Weerasekara S., Hua D.H., Groutas W.C., Chang K.O., Pedersen N.C. (2016). Reversal of the Progression of Fatal Coronavirus Infection in Cats by a Broad-Spectrum Coronavirus Protease Inhibitor. PLoS Pathog..

[137] Benetka V., Kolodziejek J., Walk K., Rennhofer M., Mostl K. (2006). M gene analysis of atypical strains of feline and canine coronavirus circulating in an Austrian animal shelter. Vet. Rec..

[138] Horzinek M.C., Osterhaus A.D. (1979). Feline infectious peritonitis: A worldwide serosurvey. Am. J. Vet. Res..

[139] Levy J.K., Crawford P.C., Lappin M.R., Dubovi E.J., Levy M.G., Alleman R., Tucker S.J., Clifford E.L. (2008). Infectious diseases of dogs and cats on Isabela Island, Galapagos. J. Vet. Intern. Med..

[140] Addie D.D., McDonald M., Audhuy S., Burr P., Hollins J., Kovacic R., Lutz H., Luxton Z., Mazar S., Meli M.L. (2012). Quarantine protects Falkland Islands (Malvinas) cats from feline coronavirus infection. J. Feline Med. Surg..

[141] Sharif S., Arshad S.S., Hair-Bejo M., Omar A.R., Zeenathul N.A., Hafidz M.A. (2009). Prevalence of feline coronavirus in two cat populations in Malaysia. J. Feline Med. Surg..

[142] Herrewegh A.A., de Groot R.J., Cepica A., Egberink H.F., Horzinek M.C., Rottier P.J. (1995). Detection of feline coronavirus RNA in feces, tissues, and body fluids of naturally infected cats by reverse transcriptase PCR. J. Clin. Microbiol..

[143] Cave T.A., Golder M.C., Simpson J., Addie D.D. (2004). Risk factors for feline coronavirus seropositivity in cats relinquished to a UK rescue charity. J. Feline Med. Surg..

[144] Bell E.T., Toribio J.A., White J.D., Malik R., Norris J.M. (2006). Seroprevalence study of feline coronavirus in owned and feral cats in Sydney, Australia. Aust. Vet. J..

[145] Addie D.D. (2000). Clustering of feline coronaviruses in multicat households. Vet. J..

[146] Taharaguchi S., Soma T., Hara M. (2012). Prevalence of feline coronavirus antibodies in Japanese domestic cats during the past decade. J. Vet. Med. Sci..

[147] Spada E., Carrera Nulla A., Perego R., Baggiani L., Proverbio D. (2022). Evaluation of Association between Blood Phenotypes A, B and AB and Feline Coronavirus Infection in Cats. Pathogens.

[148] Kokkinaki K.C.G., Saridomichelakis M.N., Mylonakis M.E., Leontides L., Xenoulis P.G. (2023). Seroprevalence of and risk factors for feline coronavirus infection in cats from Greece. Comp. Immunol. Microbiol. Infect. Dis..

[149] Kennedy M., Citino S., McNabb A.H., Moffatt A.S., Gertz K., Kania S. (2002). Detection of feline coronavirus in captive Felidae in the USA. J. Vet. Diagn. Investig..

[150] Evermann J.F., Heeney J.L., Roelke M.E., McKeirnan A.J., O’Brien S.J. (1988). Biological and pathological consequences of feline infectious peritonitis virus infection in the cheetah. Arch. Virol..

[151] Paul A., Stayt J. (2019). The intestinal microbiome in dogs and cats with diarrhoea as detected by a faecal polymerase chain reaction-based panel in Perth, Western Australia. Aust. Vet. J..

[152] Posch A., Posch U., Kubber-Heiss A., Stur I., Seiser M., Mostl K. (2001). Feline Coronaviren: Differenzierung der Typen I und II mittels RT-PCR und deren Vorkommen in österreichischen Katzenpopulationen. Vet. Med. Austria.

[153] Raukar J. (2021). Prevalence of feline coronavirus, feline leukemia virus, and feline immunodeficiency virus in client-owned cats in Croatia. J. Adv. Nat. Sci..

[154] Felten S., Klein-Richers U., Unterer S., Bergmann M., Leutenegger C.M., Pantchev N., Balzer J., Zablotski Y., Hofmann-Lehmann R., Hartmann K. (2022). Role of Feline Coronavirus as Contributor to Diarrhea in Cats from Breeding Catteries. Viruses.

[155] Avizeh R., Mosallanejad B., Seyfiabad Shapouri M.R. (2012). Antibody detection of feline infectious peritonitis virus (FIPV) in sera of companion cats in Ahvaz, south west of Iran. Arch. Razi Inst..

[156] Baneth G., Kass P.H., Steinfeld D., Besser M. (1999). A seroepidemiological study of feline coronavirus, feline immunodeficiency virus and feline leukemia virus among cats in Israel. Isr. J. Vet. Med..

[157] Giordano A., Spagnolo V., Colombo A., Paltrinieri S. (2004). Changes in some acute phase protein and immunoglobulin concentrations in cats affected by feline infectious peritonitis or exposed to feline coronavirus infection. Vet. J..

[158] Pratelli A. (2008). Comparison of serologic techniques for the detection of antibodies against feline coronaviruses. J. Vet. Diag. Investig..

[159] Osterhaus A.D., Horzinek M.C., Reynolds D.J. (1977). Seroepidemiology of feline infectious peritonitis virus infections using transmissible gastroenteritis virus as antigen. J. Vet. Med. Ser. B.

[160] Ström Holst B., Englund L., Palacios S., Renstrom L., Berndtsson L.T. (2006). Prevalence of antibodies against feline coronavirus and *Chlamydophila felis* in Swedish cats. J. Feline Med. Surg..

[161] Pratelli A., Yesilbag K., Siniscalchi M., Yalcm E., Yilmaz Z. (2009). Prevalence of feline coronavirus antibodies in cats in Bursa province, Turkey, by an enzyme-linked immunosorbent assay. J. Feline Med. Surg..

[162] Tekelioglu B.K., Berriatua E., Turan N., Helps C.R., Kocak M., Yilmaz H. (2015). A retrospective clinical and epidemiological study on feline coronavirus (FCoV) in cats in Istanbul, Turkey. Prev. Vet. Med..

[163] Sparkes A.H., Gruffydd-Jones T.J., Harbour D.A. (1992). Feline coronavirus antibodies in UK cats. Vet. Rec..

[164] Muirden A. (2002). Prevalence of feline leukaemia virus and antibodies to feline immunodeficiency virus and feline coronavirus in stray cats sent to an RSPCA hospital. Vet. Rec..

[165] Paris J.K., Wills S., Balzer H.J., Shaw D.J., Gunn-Moore D.A. (2014). Enteropathogen co-infection in UK cats with diarrhoea. BMC Vet. Res..

[166] Addie D.D., Toth S., Herrewegh A.A., Jarrett O. (1996). Feline coronavirus in the intestinal contents of cats with feline infectious peritonitis. Vet. Rec..

[167] Addie D.D., Toth S., Murray G.D., Jarrett O. (1995). Risk of feline infectious peritonitis in cats naturally infected with feline coronavirus. Am. J. Vet. Res..

[168] Kass P.H., Dent T.H. (1995). The epidemiology of feline infectious peritonitis in catteries. Feline Pract..

[169] Foley J.E., Poland A., Carlson J., Pedersen N.C. (1997). Risk factors for feline infectious peritonitis among cats in multiple-cat environments with endemic feline enteric coronavirus. J. Am. Vet. Med. Assoc..

[170] Pedersen N.C. (1976). Serologic studies of naturally occurring feline infectious peritonitis. Am. J. Vet. Res..

[171] Pedersen N.C., Liu H., Gandolfi B., Lyons L.A. (2014). The influence of age and genetics on natural resistance to experimentally induced feline infectious peritonitis. Vet. Immunol. Immunopathol..

[172] Foley J., Pedersen N. (1996). The inheritence of susceptibility to feline infectious peritonitis in purebred catteries. Feline Pract..

[173] Pedersen N.C., Liu H., Durden M., Lyons L.A. (2016). Natural resistance to experimental feline infectious peritonitis virus infection is decreased rather than increased by positive genetic selection. Vet. Immunol. Immunopathol..

[174] Krentz D., Zwicklbauer K., Felten S., Bergmann M., Dorsch R., Hofmann-Lehmann R., Meli M.L., Spiri A.M., von Both U., Alberer M. (2022). Clinical Follow-Up and Postmortem Findings in a Cat That Was Cured of Feline Infectious Peritonitis with an Oral Antiviral Drug Containing GS-441524. Viruses.

[175] Sabshin S.J., Levy J.K., Tupler T., Tucker S.J., Greiner E.C., Leutenegger C.M. (2012). Enteropathogens identified in cats entering a Florida animal shelter with normal feces or diarrhea. J. Am. Vet. Med. Assoc..

[176] Pedersen N.C. (1987). Virologic and immunologic aspects of feline infectious peritonitis virus infection. Adv. Exp. Med. Biol..

[177] Malbon A.J., Fonfara S., Meli M.L., Hahn S., Egberink H., Kipar A. (2019). Feline Infectious Peritonitis as a Systemic Inflammatory Disease: Contribution of Liver and Heart to the Pathogenesis. Viruses.

[178] Malbon A.J., Michalopoulou E., Meli M.L., Barker E.N., Tasker S., Baptiste K., Kipar A. (2020). Colony Stimulating Factors in Early Feline Infectious Peritonitis Virus Infection of Monocytes and in End Stage Feline Infectious Peritonitis; A Combined In Vivo And In Vitro Approach. Pathogens.

[179] Dewerchin H.L., Cornelissen E., Nauwynck H.J. (2005). Replication of feline coronaviruses in peripheral blood monocytes. Arch. Virol..

[180] Cornelissen E., Dewerchin H.L., Van Hamme E., Nauwynck H.J. (2007). Absence of surface expression of feline infectious peritonitis virus (FIPV) antigens on infected cells isolated from cats with FIP. Vet. Microbiol..

[181] Addie D.D., Jarrett O. (1995). Control of feline coronavirus infections in breeding catteries by serotesting, isolation, and early weaning. Feline Pract..

[182] Rottier P.J., Nakamura K., Schellen P., Volders H., Haijema B.J. (2005). Acquisition of macrophage tropism during the pathogenesis of feline infectious peritonitis is determined by mutations in the feline coronavirus spike protein. J. Virol..

[183] Hsieh L.E., Chueh L.L. (2014). Identification and genotyping of feline infectious peritonitis-associated single nucleotide polymorphisms in the feline interferon-gamma gene. Vet. Res..

[184] Malbon A.J., Russo G., Burgener C., Barker E.N., Meli M.L., Tasker S., Kipar A. (2020). The Effect of Natural Feline Coronavirus Infection on the Host Immune Response: A Whole-Transcriptome Analysis of the Mesenteric Lymph Nodes in Cats with and without Feline Infectious Peritonitis. Pathogens.

[185] Watanabe R., Eckstrand C., Liu H., Pedersen N.C. (2018). Characterization of peritoneal cells from cats with experimentally-induced feline infectious peritonitis (FIP) using RNA-seq. Vet. Res..

[186] Takano T., Hohdatsu T., Toda A., Tanabe M., Koyama H. (2007). TNF-alpha, produced by feline infectious peritonitis virus (FIPV)-infected macrophages, upregulates expression of type II FIPV receptor feline aminopeptidase N in feline macrophages. Virology.

[187] Takano T., Hohdatsu T., Hashida Y., Kaneko Y., Tanabe M., Koyama H. (2007). A “possible” involvement of TNF-alpha in apoptosis induction in peripheral blood lymphocytes of cats with feline infectious peritonitis. Vet. Microbiol..

[188] Takano T., Azuma N., Satoh M., Toda A., Hashida Y., Satoh R., Hohdatsu T. (2009). Neutrophil survival factors (TNF-alpha, GM-CSF, and G-CSF) produced by macrophages in cats infected with feline infectious peritonitis virus contribute to the pathogenesis of granulomatous lesions. Arch. Virol..

[189] Doki T., Takano T., Nishiyama Y., Nakamura M., Hohdatsu T. (2013). Generation, characterization and therapeutic potential of anti-feline TNF-alpha MAbs for feline infectious peritonitis. Res. Vet. Sci..

[190] Doki T., Toda M., Hasegawa N., Hohdatsu T., Takano T. (2020). Therapeutic effect of an anti-human-TNF-alpha antibody and itraconazole on feline infectious peritonitis. Arch. Virol..

[191] Vermeulen B.L., Devriendt B., Olyslaegers D.A., Dedeurwaerder A., Desmarets L.M., Favoreel H.W., Dewerchin H.L., Nauwynck H.J. (2013). Suppression of NK cells and regulatory T lymphocytes in cats naturally infected with feline infectious peritonitis virus. Vet. Microbiol..

[192] de Groot-Mijnes J.D., van Dun J.M., van der Most R.G., de Groot R.J. (2005). Natural history of a recurrent feline coronavirus infection and the role of cellular immunity in survival and disease. J. Virol..

[193] Berg A.L., Ekman K., Belak S., Berg M. (2005). Cellular composition and interferon-gamma expression of the local inflammatory response in feline infectious peritonitis (FIP). Vet. Microbiol..

[194] Dean G.A., Olivry T., Stanton C., Pedersen N.C. (2003). In vivo cytokine response to experimental feline infectious peritonitis virus infection. Vet. Microbiol..

[195] Gelain M.E., Meli M., Paltrinieri S. (2006). Whole blood cytokine profiles in cats infected by feline coronavirus and healthy non-FCoV infected specific pathogen-free cats. J. Feline Med. Surg..

[196] Gunn-Moore D.A., Caney S.M., Gruffydd-Jones T.J., Helps C.R., Harbour D.A. (1998). Antibody and cytokine responses in kittens during the development of feline infectious peritonitis (FIP). Vet. Immunol. Immunopathol..

[197] Kiss I., Poland A.M., Pedersen N.C. (2004). Disease outcome and cytokine responses in cats immunized with an avirulent feline infectious peritonitis virus (FIPV)-UCD1 and challenge-exposed with virulent FIPV-UCD8. J. Feline Med. Surg..

[198] Giordano A., Paltrinieri S. (2009). Interferon-gamma in the serum and effusions of cats with feline coronavirus infection. Vet. J..

[199] Kedward-Dixon H., Barker E.N., Tasker S., Kipar A., Helps C.R. (2020). Evaluation of polymorphisms in inflammatory mediator and cellular adhesion genes as risk factors for feline infectious peritonitis. J. Feline Med. Surg..

[200] Barker E.N., Lait P., Ressel L., Blackwell E.J., Tasker S., Kedward-Dixon H., Kipar A., Helps C.R. (2020). Evaluation of Interferon-Gamma Polymorphisms as a Risk Factor in Feline Infectious Peritonitis Development in Non-Pedigree Cats—A Large Cohort Study. Pathogens.

[201] Wang Y.T., Hsieh L.E., Dai Y.R., Chueh L.L. (2014). Polymorphisms in the feline TNFA and CD209 genes are associated with the outcome of feline coronavirus infection. Vet. Res..

[202] Pearson M., LaVoy A., Evans S., Vilander A., Webb C., Graham B., Musselman E., LeCureux J., VandeWoude S., Dean G.A. (2019). Mucosal Immune Response to Feline Enteric Coronavirus Infection. Viruses.

[203] Stoddart M.E., Gaskell R.M., Harbour D.A., Pearson G.R. (1988). The sites of early viral replication in feline infectious peritonitis. Vet. Microbiol..

[204] Addie D.D., Dennis J.M., Toth S., Callanan J.J., Reid S., Jarrett O. (2000). Long-term impact on a closed household of pet cats of natural infection with feline coronavirus, feline leukaemia virus and feline immunodeficiency virus. Vet. Rec..

[205] Gonon V., Duquesne V., Klonjkowski B., Monteil M., Aubert A., Eloit M. (1999). Clearance of infection in cats naturally infected with feline coronaviruses is associated with an anti-S glycoprotein antibody response. J. Gen. Virol..

[206] Vennema H., de Groot R.J., Harbour D.A., Dalderup M., Gruffydd-Jones T., Horzinek M.C., Spaan W.J. (1990). Early death after feline infectious peritonitis virus challenge due to recombinant vaccinia virus immunization. J. Virol..

[207] De Groot R.J., Horzinek M.C., Siddell S.G. (1995). Feline infectious peritonitis. The Coronaviridae.

[208] Weiss R.C., Scott F.W. (1981). Pathogenesis of feline infetious peritonitis: Pathologic changes and immunofluorescence. Am. J. Vet. Res..

[209] Addie D.D., Toth S., Murray G.D., Jarrett O. (1995). The risk of typical and antibody-enhanced feline infectious peritonitis among cats from feline coronavirus endemic households. Feline Pract..

[210] Andersen L.A., Levy J.K., McManus C.M., McGorray S.P., Leutenegger C.M., Piccione J., Blackwelder L.K., Tucker S.J. (2018). Prevalence of enteropathogens in cats with and without diarrhea in four different management models for unowned cats in the southeast United States. Vet. J..

[211] Tasker S., Addie D.D., Egberink H., Hartmann K., Hofmann-Lehmann R., Hosie M.J., Truyen U., Belak S., Boucraut-Baralon C., Frymus T. (2022). ABCD FIP Diagnostic Tools. http://www.abcdcatsvets.org/wp-content/uploads/2022/02/FIP_diagnostic_tool_Dec21.pdf.

[212] Cerna P., Ayoob A., Baylor C., Champagne E., Hazanow S., Heidel R.E., Wirth K., Legendre A.M., Gunn-Moore D.A. (2022). Retrospective Survival Analysis of Cats with Feline Infectious Peritonitis Treated with Polyprenyl Immunostimulant That Survived over 365 Days. Pathogens.

[213] Spencer S.E., Knowles T., Ramsey I.K., Tasker S. (2017). Pyrexia in cats: Retrospective analysis of signalment, clinical investigations, diagnosis and influence of prior treatment in 106 referred cases. J. Feline Med. Surg..

[214] Beatty J., Barrs V. (2010). Pleural effusion in the cat: A practical approach to determining aetiology. J. Feline Med. Surg..

[215] Konig A., Hartmann K., Mueller R.S., Wess G., Schulz B.S. (2019). Retrospective analysis of pleural effusion in cats. J. Feline Med. Surg..

[216] Fischer Y., Wess G., Hartmann K. (2012). Pericardial effusion in a cat with feline infectious peritonitis. Schweiz. Arch. Tierheilk..

[217] Trulove S.G., Mccahon H.A., Nichols R., Fooshee S.K. (1992). Pyogranulomatous Pneumonia Associated with Generalized Noneffusive Feline Infectious Peritonitis. Feline Pract..

[218] Macdonald E.S., Norris C.R., Berghaus R.B., Griffey S.M. (2003). Clinicopathologic and radiographic features and etiologic agents in cats with histologically confirmed infectious pneumonia: 39 cases (1991–2000). J. Am. Vet. Med. Assoc..

[219] Zwicklbauer K., Krentz D., Bergmann M., Felten S., Dorsch R., Fischer A., Hofmann-Lehmann R., Meli M.L., Spiri A.M., Alberer M. (2023). Long-term follow-up of cats in complete remission after treatment of feline infectious peritonitis with oral GS-441524. J. Feline Med. Surg..

[220] Harvey C.J., Lopez J.W., Hendrick M.J. (1996). An uncommon intestinal manifestation of feline infectious peritonitis: 26 cases (1986–1993). J. Am. Vet. Med. Assoc..

[221] Kipar A., Koehler K., Bellmann S., Reinacher M. (1999). Feline infectious peritonitis presenting as a tumour in the abdominal cavity. Vet. Rec..

[222] Cohen T.M., Blois S., Vince A.R. (2016). Fatal extraintestinal toxoplasmosis in a young male cat with enlarged mesenteric lymph nodes. Can. Vet. J..

[223] O’Halloran C., Gunn-Moore D. (2017). Mycobacteria in cats: An update. Practice.

[224] Hugo T.B., Heading K.L. (2015). Prolonged survival of a cat diagnosed with feline infectious peritonitis by immunohistochemistry. Can. Vet. J..

[225] Bauer B.S., Kerr M.E., Sandmeyer L.S., Grahn B.H. (2013). Positive immunostaining for feline infectious peritonitis (FIP) in a Sphinx cat with cutaneous lesions and bilateral panuveitis. Vet. Ophthalmol..

[226] Cannon M.J., Silkstone M.A., Kipar A.M. (2005). Cutaneous lesions associated with coronavirus-induced vasculitis in a cat with feline infectious peritonitis and concurrent feline immunodeficiency virus infection. J. Feline Med. Surg..

[227] Declercq J., De Bosschere H., Schwarzkopf I., Declercq L. (2008). Papular cutaneous lesions in a cat associated with feline infectious peritonitis. Vet. Dermatol..

[228] Redford T., Al-Dissi A.N. (2019). Feline infectious peritonitis in a cat presented because of papular skin lesions. Can. Vet. J..

[229] Trotman T.K., Mauldin E., Hoffmann V., Del Piero F., Hess R.S. (2007). Skin fragility syndrome in a cat with feline infectious peritonitis and hepatic lipidosis. Vet. Dermatol..

[230] Avila V.A., Rissi D.R. (2020). Ulcerative dermatitis due to feline infectious peritonitis virus infection in a cat. Braz. J. Vet. Pathol..

[231] Bae H., Kim J., Chun D., Jung D.I., Park J., Young Kim D., Yu D. (2021). Idiopathic ulcerative dermatitis in a cat with feline infectious peritonitis. Vet. Med. Sci..

[232] Rota A., Paltrinieri S., Jussich S., Ubertalli G., Appino S. (2008). Priapism in a castrated cat associated with feline infectious peritonitis. J. Feline Med. Surg..

[233] Doenges S.J., Weber K., Dorsch R., Fux R., Fischer A., Matiasek L.A., Matiasek K., Hartmann K. (2016). Detection of feline coronavirus in cerebrospinal fluid for diagnosis of feline infectious peritonitis in cats with and without neurological signs. J. Feline Med. Surg..

[234] Foley J.E., Leutenegger C. (2001). A review of coronavirus infection in the central nervous system of cats and mice. J. Vet. Intern. Med..

[235] Ives E.J., Vanhaesebrouck A.E., Cian F. (2013). Immunocytochemical demonstration of feline infectious peritonitis virus within cerebrospinal fluid macrophages. J. Feline Med. Surg..

[236] Kent M. (2009). The cat with neurological manifestations of systemic disease. Key conditions impacting on the CNS. J. Feline Med. Surg..

[237] Negrin A., Cherubini G.B., Lamb C., Benigni L., Adams V., Platt S. (2010). Clinical signs, magnetic resonance imaging findings and outcome in 77 cats with vestibular disease: A retrospective study. J. Feline Med. Surg..

[238] Negrin A., Lamb C.R., Cappello R., Cherubini G.B. (2007). Results of magnetic resonance imaging in 14 cats with meningoencephalitis. J. Feline Med. Surg..

[239] Rissi D.R. (2018). A retrospective study of the neuropathology and diagnosis of naturally occurring feline infectious peritonitis. J. Vet. Diag. Investig..

[240] Timmann D., Cizinauskas S., Tomek A., Doherr M., Vandevelde M., Jaggy A. (2008). Retrospective analysis of seizures associated with feline infectious peritonitis in cats. J. Feline Med. Surg..

[241] Grapes N.J., Taylor-Brown F.E., Volk H.A., De Decker S. (2021). Clinical reasoning in feline vestibular syndrome: Which presenting features are the most important?. J. Feline Med. Surg..

[242] Mella S.L., Cardy T.J., Volk H.A., De Decker S. (2020). Clinical reasoning in feline spinal disease: Which combination of clinical information is useful?. J. Feline Med. Surg..

[243] Jinks M.R., English R.V., Gilger B.C. (2016). Causes of endogenous uveitis in cats presented to referral clinics in North Carolina. Vet. Ophthalmol..

[244] Wegg M.L., Jeanes E.C., Pollard D., Fleming L., Dawson C. (2021). A multicenter retrospective study into endogenous causes of uveitis in cats in the United Kingdom: Ninety two cases. Vet. Ophthalmol..

[245] Carossino M., Del Piero F., Lee J., Needle D.B., Levine J.M., Riis R.R., Maes R., Wise A.G., Mullaney K., Ferracone J. (2022). Relationship between Uveal Inflammation and Viral Detection in 30 Cats with Feline Infectious Peritonitis. Pathogens.

[246] Ali K.M., Abu-Seida A.M., Abuowarda M. (2021). Feline ocular toxoplasmosis: Seroprevalence, diagnosis and treatment outcome of 60 clinical cases. Pol. J. Vet. Sci.

[247] Ernandes M.A., Cantoni A.M., Armando F., Corradi A., Ressel L., Tamborini A. (2019). Feline coronavirus-associated myocarditis in a domestic longhair cat. J. Feline Med. Surg. Open Rep..

[248] Rohrer C., Suter P.F., Lutz H. (1993). The diagnosis of feline infectious peritonitis (FIP): A retrospective and prospective study. Kleintierpraxis.

[249] Hartmann K., Binder C., Hirschberger J., Cole D., Reinacher M., Schroo S., Frost J., Egberink H., Lutz H., Hermanns W. (2003). Comparison of different tests to diagnose feline infectious peritonitis. J. Vet. Intern. Med..

[250] Pedersen N.C. (2014). An update on feline infectious peritonitis: Diagnostics and therapeutics. Vet. J..

[251] Tasker S. (2018). Diagnosis of feline infectious peritonitis: Update on evidence supporting available tests. J. Feline Med. Surg..

[252] Johnson L.R., Epstein S.E., Reagan K.L. (2023). Etiology and effusion characteristics in 29 cats and 60 dogs with pyothorax (2010–2020). J. Vet. Intern. Med..

[253] Savary K.C., Sellon R.K., Law J.M. (2001). Chylous abdominal effusion in a cat with feline infectious peritonitis. J. Am. Anim. Hosp. Assoc..

[254] Jähne S., Felten S., Bergmann M., Erber K., Matiasek K., Meli M.L., Hofmann-Lehmann R., Hartmann K. (2022). Detection of Feline Coronavirus Variants in Cats without Feline Infectious Peritonitis. Viruses.

[255] Emmler L., Felten S., Matiasek K., Balzer H.J., Pantchev N., Leutenegger C., Hartmann K. (2020). Feline coronavirus with and without spike gene mutations detected by real-time RT-PCRs in cats with feline infectious peritonitis. J. Feline Med. Surg..

[256] Stranieri A., Giordano A., Paltrinieri S., Giudice C., Cannito V., Lauzi S. (2018). Comparison of the performance of laboratory tests in the diagnosis of feline infectious peritonitis. J. Vet. Diag. Investig..

[257] Dunbar D., Addie D., Hosie M., Weir W. A Machine Learning Approach to Enhancing Feline Infectious Peritonitis Diagnosis. Proceedings of the Fifth International Society for Companion Animal Infectious Diseases Symposium (Abstract).

[258] Rohrer C. (1992). Die Diagnostik der Felinen Infektiösen Peritonitis (FIP): Eine Restrospektive Studie.

[259] Sparkes A.H., Gruffydd-Jones T.J., Harbour D.A. (1994). An appraisal of the value of laboratory tests in the diagnosis of feline infectious peritonitis. J. Am. Anim. Hosp. Assoc..

[260] Jeffery U., Deitz K., Hostetter S. (2012). Positive predictive value of albumin: Globulin ratio for feline infectious peritonitis in a mid-western referral hospital population. J. Feline Med. Surg..

[261] Stranieri A., Giordano A., Bo S., Braghiroli C., Paltrinieri S. (2017). Frequency of electrophoretic changes consistent with feline infectious peritonitis in two different time periods (2004–2009 vs 2013–2014). J. Feline Med. Surg..

[262] Taylor S.S., Tappin S.W., Dodkin S.J., Papasouliotis K., Casamian-Sorrosal D., Tasker S. (2010). Serum protein electrophoresis in 155 cats. J. Feline Med. Surg..

[263] Hartmann K., Pennisi M.G., Dorsch R. (2020). Infectious Agents in Feline Chronic Kidney Disease. Adv. Small Anim. Care.

[264] Duthie S., Eckersall P.D., Addie D.D., Lawrence C.E., Jarrett O. (1997). Value of alpha 1-acid glycoprotein in the diagnosis of feline infectious peritonitis. Vet. Rec..

[265] Giori L., Giordano A., Giudice C., Grieco V., Paltrinieri S. (2011). Performances of different diagnostic tests for feline infectious peritonitis in challenging clinical cases. J. Small Anim. Pract..

[266] Hazuchova K., Held S., Neiger R. (2017). Usefulness of acute phase proteins in differentiating between feline infectious peritonitis and other diseases in cats with body cavity effusions. J. Feline Med. Surg..

[267] Paltrinieri S., Giordano A., Tranquillo V., Guazzetti S. (2007). Critical assessment of the diagnostic value of feline alpha1-acid glycoprotein for feline infectious peritonitis using the likelihood ratios approach. J. Vet. Diag. Investig..

[268] Paltrinieri S., Metzger C., Battilani M., Pocacqua V., Gelain M.E., Giordano A. (2007). Serum alpha1-acid glycoprotein (AGP) concentration in non-symptomatic cats with feline coronavirus (FCoV) infection. J. Feline Med. Surg..

[269] Ceciliani F., Grossi C., Giordano A., Pocacqua V., Paltrinieri S. (2004). Decreased sialylation of the acute phase protein alpha1-acid glycoprotein in feline infectious peritonitis (FIP). Vet. Immunol. Immunopathol..

[270] Rossi G., Paltrinieri S. (2009). Total sialic acid: An acute phase reactant in cats with a possible role in feline coronavirus infection. Can. J. Vet. Res..

[271] Thalmeier S., Güssow A., Häuser M.K., Bauer N., Hazuchova K. (2023). Cat alpha-1-acid glycoprotein enzyme-linked immunosorbent assay: Performance characteristics and reference intervals. J. Feline Med. Surg..

[272] Shelly S.M., Scarlettkranz J., Blue J.T. (1988). Protein Electrophoresis on Effusions from Cats as a Diagnostic-Test for Feline Infectious Peritonitis. J. Am. Anim. Hosp. Assoc..

[273] Paltrinieri S., Cammarata M.P., Cammarata G. (1999). In vivo diagnosis of feline infectious peritonitis by comparison of protein content, cytology, and direct immunofluorescence test on peritoneal and pleural effusions. J. Vet. Diag. Investig..

[274] Fischer Y., Sauter-Louis C., Hartmann K. (2012). Diagnostic accuracy of the Rivalta test for feline infectious peritonitis. Vet. Clin. Pathol..

[275] Fischer Y., Weber K., Sauter-Louis C., Hartmann K. (2013). The Rivalta’s test as a diagnostic variable in feline effusions—Evaluation of optimum reaction and storage conditions. Tierärztliche Praxis.

[276] Giordano A., Paltrinieri S., Bertazzolo W., Milesi E., Parodi M. (2005). Sensitivity of Tru-cut and fine needle aspiration biopsies of liver and kidney for diagnosis of feline infectious peritonitis. Vet. Clin. Pathol..

[277] Rusbridge C. (1997). Collection and interpretation of cerebrospinal fluid in cats and dogs. Practice.

[278] Hoey C., Nye G., Fadda A., Bradshaw J., Barker E.N. (2020). Subarachnoid diverticulum associated with feline infectious peritonitis in a Siberian cat. J. Feline Med. Surg. Open Rep..

[279] Penderis J. (2009). The Wobbly Cat. Diagnostic and therapeutic approach to generalised ataxia. J. Feline Med. Surg..

[280] Felten S., Matiasek K., Leutenegger C.M., Sangl L., Herre S., Dorfelt S., Fischer A., Hartmann K. (2021). Diagnostic Value of Detecting Feline Coronavirus RNA and Spike Gene Mutations in Cerebrospinal Fluid to Confirm Feline Infectious Peritonitis. Viruses.

[281] Singh M., Foster D.J., Child G., Lamb W.A. (2005). Inflammatory cerebrospinal fluid analysis in cats: Clinical diagnosis and outcome. J. Feline Med. Surg..

[282] Boettcher I.C., Steinberg T., Matiasek K., Greene C.E., Hartmann K., Fischer A. (2007). Use of anti-coronavirus antibody testing of cerebrospinal fluid for diagnosis of feline infectious peritonitis involving the central nervous system in cats. J. Am. Vet. Med. Assoc..

[283] Linn-Pearl R.N., Powell R.M., Newman H.A., Gould D.J. (2015). Validity of aqueocentesis as a component of anterior uveitis investigation in dogs and cats. Vet. Ophthalmol..

[284] Ferreira A., Marwood R., Batchelor D., Maddox T., Mortier J.R. (2020). Prevalence and clinical significance of the medullary rim sign identified on ultrasound of feline kidneys. Vet. Rec..

[285] Hung L., Hopper B.J., Lenard Z. (2022). Retrospective analysis of radiographic signs in feline pleural effusions to predict disease aetiology. BMC Vet. Res..

[286] Negrin A., Schatzberg S., Platt S.R. (2009). The paralyzed cat. Neuroanatomic diagnosis and specific spinal cord diseases. J. Feline Med. Surg..

[287] Paltrinieri S., Cammarata M.P., Cammarata G., Comazzi S. (1998). Some aspects of humoral and cellular immunity in naturally occuring feline infectious peritonitis. Vet. Immunol. Immunopathol..

[288] Mesquita L.P., Hora A.S., de Siqueira A., Salvagni F.A., Brandao P.E., Maiorka P.C. (2016). Glial response in the central nervous system of cats with feline infectious peritonitis. J. Feline Med. Surg..

[289] Stranieri A., Scavone D., Paltrinieri S., Giordano A., Bonsembiante F., Ferro S., Gelain M.E., Meazzi S., Lauzi S. (2020). Concordance between Histology, Immunohistochemistry, and RT-PCR in the Diagnosis of Feline Infectious Peritonitis. Pathogens.

[290] Felten S., Leutenegger C.M., Balzer H.J., Pantchev N., Matiasek K., Wess G., Egberink H., Hartmann K. (2017). Sensitivity and specificity of a real-time reverse transcriptase polymerase chain reaction detecting feline coronavirus mutations in effusion and serum/plasma of cats to diagnose feline infectious peritonitis. BMC Vet. Res..

[291] Milliron S.M., Seyler Z.G., Myers A.N., Rodrigues Hoffmann A., Hnot M., Wiener D.J. (2021). Pyogranulomatous panniculitis in a domestic cat associated with *Pseudomonas luteola* infection. Vet. Dermatol..

[292] Giuliano A., Watson P., Owen L., Skelly B., Davison L., Dobson J., Costantino-Casas F. (2020). Idiopathic sterile pyogranuloma in three domestic cats. J. Small Anim. Pract..

[293] Kipar A., Meli M.L. (2014). Feline Infectious Peritonitis: Still an Enigma?. Vet. Pathol..

[294] Tammer R., Evensen O., Lutz H., Reinacher M. (1995). Immunohistological demonstration of feline infectious peritonitis virus antigen in paraffin-embedded tissues using feline ascites or murine monoclonal antibodies. Vet. Immunol. Immunopathol..

[295] Paltrinieri S., Grieco V., Comazzi S., Cammarata Parodi M. (2001). Laboratory profiles in cats with different pathological and immunohistochemical findings due to feline infectious peritonitis (FIP). J. Feline Med. Surg..

[296] Tasker S., Dowgray N., Dean R., Roberts M., Stavisky J. (2018). Managing feline coronavirus and feline infectious peritonitis in the multi-cat/shelter environment. BSAVA Manual of Canine and Feline Shelter Medicine: Principles of Health and Welfare in a Multi-Animal Environment.

[297] Felten S., Matiasek K., Gruendl S., Sangl L., Wess G., Hartmann K. (2017). Investigation into the utility of an immunocytochemical assay in body cavity effusions for diagnosis of feline infectious peritonitis. J. Feline Med. Surg..

[298] Hirschberger J., Hartmann K., Wilhelm N., Frost J., Lutz H., Kraft W. (1995). Clinical symptoms and diagnosis of feline infectious peritonitis. Tierarztl. Prax..

[299] Litster A.L., Pogranichniy R., Lin T.L. (2013). Diagnostic utility of a direct immunofluorescence test to detect feline coronavirus antigen in macrophages in effusive feline infectious peritonitis. Vet. J..

[300] Parodi M.C., Cammarata G., Paltrinieri S., Lavazza A., Ape F. (1993). Using Direct Immunofluorescence to Detect Coronaviruses in Peritoneal and Pleural Effusions. J. Small Anim. Pract..

[301] Hellemans A., Acar D.D., Stroobants V.J.E., Theuns S., Desmarets L.M.B., Nauwynck H.J. (2020). A comparative study of techniques used for the diagnosis of effusive feline infectious peritonitis. Vlaams. Diergeneeskd. Tijdschr.

[302] Howell M., Evans S.J.M., Cornwall M., Santangelo K.S. (2020). Multiplex fluorescent immunocytochemistry for the diagnosis of feline infectious peritonitis: Determining optimal storage conditions. Vet. Clin. Pathol..

[303] Gruendl S., Matiasek K., Matiasek L., Fischer A., Felten S., Jurina K., Hartmann K. (2016). Diagnostic utility of cerebrospinal fluid immunocytochemistry for diagnosis of feline infectious peritonitis manifesting in the central nervous system. J. Feline Med. Surg..

[304] Felten S., Matiasek K., Gruendl S., Sangl L., Hartmann K. (2018). Utility of an immunocytochemical assay using aqueous humor in the diagnosis of feline infectious peritonitis. Vet. Ophthalmol..

[305] Sangl L., Felten S., Matiasek K., Dorfelt S., Bergmann M., Balzer H.J., Pantchev N., Leutenegger C., Hartmann K. (2020). Detection of feline coronavirus RNA, spike gene mutations, and feline Coronavirus antigen in macrophages in aqueous humor of cats in the diagnosis of feline infectious peritonitis. J. Vet. Diag. Investig..

[306] Barker E.N., Tasker S. Diagnosing FIP: Has recent research made it any easier? In Proceedings of the Amercian College of Veterinary Internal Medicine Forum.

[307] Desmarets L.M., Vermeulen B.L., Theuns S., Conceicao-Neto N., Zeller M., Roukaerts I.D., Acar D.D., Olyslaegers D.A., Van Ranst M., Matthijnssens J. (2016). Experimental feline enteric coronavirus infection reveals an aberrant infection pattern and shedding of mutants with impaired infectivity in enterocyte cultures. Sci. Rep..

[308] Gunther S., Felten S., Wess G., Hartmann K., Weber K. (2018). Detection of feline Coronavirus in effusions of cats with and without feline infectious peritonitis using loop-mediated isothermal amplification. J. Virol. Methods.

[309] Stranieri A., Lauzi S., Giordano A., Paltrinieri S. (2017). Reverse transcriptase loop-mediated isothermal amplification for the detection of feline coronavirus. J. Virol. Methods.

[310] Rapichai W., Saejung W., Khumtong K., Boonkaewwan C., Tuanthap S., Lieberzeit P.A., Choowongkomon K., Rattanasrisomporn J. (2022). Development of Colorimetric Reverse Transcription Loop-Mediated Isothermal Amplification Assay for Detecting Feline Coronavirus. Animals.

[311] Doenges S.J., Weber K., Dorsch R., Fux R., Hartmann K. (2017). Comparison of real-time reverse transcriptase polymerase chain reaction of peripheral blood mononuclear cells, serum and cell-free body cavity effusion for the diagnosis of feline infectious peritonitis. J. Feline Med. Surg..

[312] Felten S., Weider K., Doenges S., Gruendl S., Matiasek K., Hermanns W., Mueller E., Matiasek L., Fischer A., Weber K. (2017). Detection of feline coronavirus spike gene mutations as a tool to diagnose feline infectious peritonitis. J. Feline Med. Surg..

[313] Pedersen N.C., Eckstrand C., Liu H., Leutenegger C., Murphy B. (2015). Levels of feline infectious peritonitis virus in blood, effusions, and various tissues and the role of lymphopenia in disease outcome following experimental infection. Vet. Microbiol..

[314] Gut M., Leutenegger C.M., Huder J.B., Pedersen N.C., Lutz H. (1999). One-tube fluorogenic reverse transcription-polymerase chain reaction for the quantitation of feline coronaviruses. J. Virol. Methods.

[315] Simons F.A., Vennema H., Rofina J.E., Pol J.M., Horzinek M.C., Rottier P.J., Egberink H.F. (2005). A mRNA PCR for the diagnosis of feline infectious peritonitis. J. Virol. Methods.

[316] Longstaff L., Porter E., Crossley V.J., Hayhow S.E., Helps C.R., Tasker S. (2017). Feline coronavirus quantitative reverse transcriptase polymerase chain reaction on effusion samples in cats with and without feline infectious peritonitis. J. Feline Med. Surg..

[317] Sangl L., Matiasek K., Felten S., Grundl S., Bergmann M., Balzer H.J., Pantchev N., Leutenegger C.M., Hartmann K. (2019). Detection of feline coronavirus mutations in paraffin-embedded tissues in cats with feline infectious peritonitis and controls. J. Feline Med. Surg..

[318] Freiche G.M., Guidez C.L., Duarte M., Le poder Y.B. (2016). Sequencing of 3c and spike genes in feline infectious peritonitis: Which samples are the most relevant for analysis? A retrospective study of 33 cases from 2008 to 2014. J. Vet. Intern. Med..

[319] Soma T., Saito N., Kawaguchi M., Sasai K. (2018). Feline coronavirus antibody titer in cerebrospinal fluid from cats with neurological signs. J. Vet. Med. Sci..

[320] Barker E., Tasker S. (2020). Update on feline infectious peritonitis. Practice.

[321] Lutz M., Steiner A.R., Cattori V., Hofmann-Lehmann R., Lutz H., Kipar A., Meli M.L. (2020). FCoV Viral Sequences of Systemically Infected Healthy Cats Lack Gene Mutations Previously Linked to the Development of FIP. Pathogens.

[322] McKay L.A., Meachem M., Snead E., Brannen T., Mutlow N., Ruelle L., Davies J.L., van der Meer F. (2020). Prevalence and mutation analysis of the spike protein in feline enteric coronavirus and feline infectious peritonitis detected in household and shelter cats in western Canada. Can. J. Vet. Res..

[323] Bell E.T., Malik R., Norris J.M. (2006). The relationship between the feline coronavirus antibody titre and the age, breed, gender and health status of Australian cats. Aust. Vet. J..

[324] Adaszek Ł., Kalinowski M., Rutkowska-Szulczyk M., Mazurek Ł., Szulc D., Staniec M., Pietras-Ożga D., Michalak K., Buczek K., Winiarczyk S. (2023). Comparison of the sensitivity of rapid tests FCoV Ab (Vet Expert) and PCR in the diagnosis of feline infectious peritonitis (FIP) in cats with the effusive form of the disease. Med. Weter..

[325] Meli M.L., Burr P., Decaro N., Graham E., Jarrett O., Lutz H., McDonald M., Addie D.D. (2013). Samples with high virus load cause a trend toward lower signal in feline coronavirus antibody tests. J. Feline Med. Surg..

[326] Lorusso E., Mari V., Losurdo M., Lanave G., Trotta A., Dowgier G., Colaianni M.L., Zatelli A., Elia G., Buonavoglia D. (2019). Discrepancies between feline coronavirus antibody and nucleic acid detection in effusions of cats with suspected feline infectious peritonitis. Res. Vet. Sci..

[327] Dye C., Siddell S.G. (2007). Genomic RNA sequence of feline coronavirus strain FCoV C1Je. J. Feline Med. Surg..

[328] Addie D.D. (2019). Feline infectious peritonitis: Answers to frequently asked questions concerning FIP and coronavirus. Vet. Nurs..

[329] Ishida T., Shibanai A., Tanaka S., Uchida K., Mochizuki M. (2004). Use of recombinant feline interferon and glucocorticoid in the treatment of feline infectious peritonitis. J. Feline Med. Surg..

[330] Legendre A.M., Kuritz T., Galyon G., Baylor V.M., Heidel R.E. (2017). Polyprenyl Immunostimulant Treatment of Cats with Presumptive Non-Effusive Feline Infectious Peritonitis In a Field Study. Front. Vet. Sci..

[331] Murphy B.G., Perron M., Murakami E., Bauer K., Park Y., Eckstrand C., Liepnieks M., Pedersen N.C. (2018). The nucleoside analog GS-441524 strongly inhibits feline infectious peritonitis (FIP) virus in tissue culture and experimental cat infection studies. Vet. Microbiol..

[332] Sorrell S., Pugalendhi S.J., Gunn-Moore D. (2022). Current treatment options for feline infectious peritonitis in the UK. Companion Anim..

[333] Pons-Hernandez M., Wyatt T., Hall A. (2022). Investigating the illicit market in veterinary medicines: An exploratory online study with pet owners in the United Kingdom. Trends Organ. Crime.

[334] Cooper G. (2021). Comments on: Dealing with black market medications. Practice.

[335] Riley N. (2021). Dealing with black market medications. In Pract..

[336] European Union (2023). Commission Implementing Regulation (EU) 2022/1255 of 19 July 2022 designating antimicrobials or groups of antimicrobials reserved for treatment of certain infections in humans, in accordance with Regulation (EU) 2019/6 of the European Parliament and of the Council. Off. J. Eur. Union.

[337] Hughes D., Howard G., Malik R. (2021). Treatment of FIP in cats with remdesivir. Veterinarian.

[338] Pedersen N.C., Kim Y., Liu H., Galasiti Kankanamalage A.C., Eckstrand C., Groutas W.C., Bannasch M., Meadows J.M., Chang K.O. (2018). Efficacy of a 3C-like protease inhibitor in treating various forms of acquired feline infectious peritonitis. J. Feline Med. Surg..

[339] Gil S., Leal R.O., Duarte A., McGahie D., Sepulveda N., Siborro I., Cravo J., Cartaxeiro C., Tavares L.M. (2013). Relevance of feline interferon omega for clinical improvement and reduction of concurrent viral excretion in retrovirus infected cats from a rescue shelter. Res. Vet. Sci..

[340] McDonagh P., Sheehy P.A., Norris J.M. (2014). Identification and characterisation of small molecule inhibitors of feline coronavirus replication. Vet. Microbiol..

[341] Delaplace M., Huet H., Gambino A., Le Poder S. (2021). Feline Coronavirus Antivirals: A Review. Pathogens.

[342] Izes A.M., Kimble B., Norris J.M., Govendir M. (2020). In vitro hepatic metabolism of mefloquine using microsomes from cats, dogs and the common brush-tailed possum (*Trichosurus vulpecula*). PLoS ONE.

[343] Yu J., Kimble B., Norris J.M., Govendir M. (2020). Pharmacokinetic Profile of Oral Administration of Mefloquine to Clinically Normal Cats: A Preliminary In-Vivo Study of a Potential Treatment for Feline Infectious Peritonitis (FIP). Animals.

[344] Izes A.M., Kimble B., Norris J.M., Govendir M. (2020). Assay validation and determination of in vitro binding of mefloquine to plasma proteins from clinically normal and FIP-affected cats. PLoS ONE.

[345] Tanaka Y., Sato Y., Osawa S., Inoue M., Tanaka S., Sasaki T. (2012). Suppression of feline coronavirus replication in vitro by cyclosporin A. Vet. Res..

[346] Tanaka Y., Sato Y., Sasaki T. (2013). Suppression of coronavirus replication by cyclophilin inhibitors. Viruses.

[347] Tanaka Y., Sato Y., Takahashi D., Matsumoto H., Sasaki T. (2015). Treatment of a case of feline infectious peritonitis with cyclosporin A. Vet. Rec. Case Rep..

[348] Ng S.W., Selvarajah G.T., Hussein M.Z., Yeap S.K., Omar A.R. (2020). In Vitro Evaluation of Curcumin-Encapsulated Chitosan Nanoparticles against Feline Infectious Peritonitis Virus and Pharmacokinetics Study in Cats. BioMed Res. Int..

[349] Takano T., Katoh Y., Doki T., Hohdatsu T. (2013). Effect of chloroquine on feline infectious peritonitis virus infection in vitro and in vivo. Antivir. Res..

[350] Takano T., Satoh K., Doki T., Tanabe T., Hohdatsu T. (2020). Antiviral Effects of Hydroxychloroquine and Type I Interferon on In Vitro Fatal Feline Coronavirus Infection. Viruses.

[351] Takano T., Akiyama M., Doki T., Hohdatsu T. (2019). Antiviral activity of itraconazole against type I feline coronavirus infection. Vet. Res..

[352] Doki T., Takahashi K., Hasegawa N., Takano T. (2022). In vitro antiviral effects of GS-441524 and itraconazole combination against feline infectious peritonitis virus. Res. Vet. Sci..

[353] Kameshima S., Kimura Y., Doki T., Takano T., Park C.H., Itoh N. (2020). Clinical efficacy of combination therapy of itraconazole and prednisolone for treating effusive feline infectious peritonitis. J. Vet. Med. Sci..

[354] Mancianti F., Pedonese F., Zullino C. (1998). Efficacy of oral administration of itraconazole to cats with dermatophytosis caused by Microsporum canis. J. Am. Vet. Med. Assoc..

[355] Hsieh L.E., Lin C.N., Su B.L., Jan T.R., Chen C.M., Wang C.H., Lin D.S., Lin C.T., Chueh L.L. (2010). Synergistic antiviral effect of Galanthus nivalis agglutinin and nelfinavir against feline coronavirus. Antivir. Res..

[356] Weiss R.C., Cox N.R., Boudreaux M.K. (1993). Toxicologic effects of ribavirin in cats. J. Vet. Pharmacol. Ther..

[357] Weiss R.C., Cox N.R., Martinez M.L. (1993). Evaluation of free or liposome-encapsulated ribavirin for antiviral therapy of experimentally induced feline infectious peritonitis. Res. Vet. Sci..

[358] Weiss R.C., Cox N.R., Oostrom-Ram T. (1990). Effect of interferon or Propionibacterium acnes on the course of experimentally induced feline infectious peritonitis in specific-pathogen-free and random-source cats. Am. J. Vet. Res..

[359] Barlough J.E., Scott F.W. (1990). Effectiveness of three antiviral agents against FIP virus in vitro. Vet. Rec..

[360] Amici C., Di Caro A., Ciucci A., Chiappa L., Castilletti C., Martella V., Decaro N., Buonavoglia C., Capobianchi M.R., Santoro M.G. (2006). Indomethacin has a potent antiviral activity against SARS coronavirus. Antivir. Ther..

[361] Mateos Gonzalez M., Sierra Gonzalo E., Casado Lopez I., Arnalich Fernandez F., Beato Perez J.L., Monge Monge D., Vargas Nunez J.A., Garcia Fenoll R., Suarez Fernandez C., Freire Castro S.J. (2021). The Prognostic Value of Eosinophil Recovery in COVID-19: A Multicentre, Retrospective Cohort Study on Patients Hospitalised in Spanish Hospitals. J. Clin. Med..

[362] Walton-Clark M., Travail V., Best M. (2022). Phenobarbital-induced autoimmune haemolytic anaemia, thrombocytopenia and peripheral lymphadenomegaly due to reactive lymphoid hyperplasia in a cat. J. Feline Med. Surg. Open Rep..

[363] Ansems K., Grundeis F., Dahms K., Mikolajewska A., Thieme V., Piechotta V., Metzendorf M.I., Stegemann M., Benstoem C., Fichtner F. (2021). Remdesivir for the treatment of COVID-19. Cochrane Database Syst. Rev..

[364] Pham H.T., Mai-Phan T.A., Vu A.K., Truong T.H., Tran M.H. (2023). Clinical use of remdesivir in COVID-19 treatment: A retrospective cohort study. BMJ Open.

[365] Wu B., Luo M., Wu F., He Z., Li Y., Xu T. (2022). Acute Kidney Injury Associated With Remdesivir: A Comprehensive Pharmacovigilance Analysis of COVID-19 Reports in FAERS. Front. Pharmacol..

[366] Brandsma I., Derr R., Zhang G., Moelijker N., Hendriks G., Osterlund T. (2022). Genotoxicity assessment of potentially mutagenic nucleoside analogues using ToxTracker(R). Toxicol. Lett..

[367] COVID-19 Treatment Guidelines Panel Coronavirus Disease 2019 (COVID-19) Treatment Guidelines. https://www.covid19treatmentguidelines.nih.gov/.

[368] European Union (2023). Regulation (EU) 2019/6 of the European Parliament and of the Council of 11 December 2018 on veterinary medicinal products and repealing Directive 2001/82/EC. Off. J. Eur. Union.

[369] Izes A.M., Yu J., Norris J.M., Govendir M. (2020). Current status on treatment options for feline infectious peritonitis and SARS-CoV-2 positive cats. Vet. Q..

[370] Li Y., Cao L., Li G., Cong F., Li Y., Sun J., Luo Y., Chen G., Li G., Wang P. (2022). Remdesivir Metabolite GS-441524 Effectively Inhibits SARS-CoV-2 Infection in Mouse Models. J. Med. Chem..

[371] Eastman R.T., Roth J.S., Brimacombe K.R., Simeonov A., Shen M., Patnaik S., Hall M.D. (2020). Remdesivir: A Review of Its Discovery and Development Leading to Emergency Use Authorization for Treatment of COVID-19. ACS Cent. Sci..

[372] Xie J., Wang Z. (2021). Can remdesivir and its parent nucleoside GS-441524 be potential oral drugs? An in vitro and in vivo DMPK assessment. Acta Pharm. Sin. B.

[373] Cook S., Wittenburg L., Yan V.C., Theil J.H., Castillo D., Reagan K.L., Williams S., Pham C.-D., Li C., Muller F.L. (2022). An Optimized Bioassay for Screening Combined Anticoronaviral Compounds for Efficacy against Feline Infectious Peritonitis Virus with Pharmacokinetic Analyses of GS-441524, Remdesivir, and Molnupiravir in Cats. Viruses.

[374] Painter W.P., Holman W., Bush J.A., Almazedi F., Malik H., Eraut N., Morin M.J., Szewczyk L.J., Painter G.R. (2021). Human Safety, Tolerability, and Pharmacokinetics of Molnupiravir, a Novel Broad-Spectrum Oral Antiviral Agent with Activity Against SARS-CoV-2. Antimicrob. Agents Chemother..

[375] Tian L., Pang Z., Li M., Lou F., An X., Zhu S., Song L., Tong Y., Fan H., Fan J. (2022). Molnupiravir and Its Antiviral Activity Against COVID-19. Front. Immunol..

[376] Cook S.E., Vogel H., Castillo D., Olsen M., Pedersen N., Murphy B.G. (2022). Investigation of monotherapy and combined anticoronaviral therapies against feline coronavirus serotype II in vitro. J. Feline Med. Surg..

[377] Kim Y., Mandadapu S.R., Groutas W.C., Chang K.O. (2013). Potent inhibition of feline coronaviruses with peptidyl compounds targeting coronavirus 3C-like protease. Antivir. Res..

[378] Jiao Z., Yan Y., Chen Y., Wang G., Wang X., Li L., Yang M., Hu X., Guo Y., Shi Y. (2022). Adaptive Mutation in the Main Protease Cleavage Site of Feline Coronavirus Renders the Virus More Resistant to Main Protease Inhibitors. J. Virol..

[379] Perera K.D., Rathnayake A.D., Liu H., Pedersen N.C., Groutas W.C., Chang K.O., Kim Y. (2019). Characterization of amino acid substitutions in feline coronavirus 3C-like protease from a cat with feline infectious peritonitis treated with a protease inhibitor. Vet. Microbiol..

[380] Day M.J., Horzinek M.C., Schultz R.D., Squires R.A. (2016). WSAVA Guidelines for the vaccination of dogs and cats. J. Small Anim. Pract..

[381] Stone A.E., Brummet G.O., Carozza E.M., Kass P.H., Petersen E.P., Sykes J., Westman M.E. (2020). 2020 AAHA/AAFP Feline Vaccination Guidelines. J. Feline Med. Surg..

[382] ABCD European Advisory Board for Cat Diseases Matrix Vaccination Guidelines. https://www.abcdcatsvets.org/wp-content/uploads/2022/11/TOOL_Vaccine-recommendations_Feb_2020_EN.pdf.

[383] Taylor S., St Denis K., Collins S., Dowgray N., Ellis S.L., Heath S., Rodan I., Ryan L. (2022). 2022 ISFM/AAFP Cat Friendly Veterinary Environment Guidelines. J. Feline Med. Surg..

[384] Chen S., Tian J., Li Z., Kang H., Zhang J., Huang J., Yin H., Hu X., Qu L. (2019). Feline Infectious Peritonitis Virus Nsp5 Inhibits Type I Interferon Production by Cleaving NEMO at Multiple Sites. Viruses.

[385] Dedeurwaerder A., Olyslaegers D.A.J., Desmarets L.M.B., Roukaerts I.D.M., Theuns S., Nauwynck H.J. (2014). ORF7-encoded accessory protein 7a of feline infectious peritonitis virus as a counteragent against IFN-alpha-induced antiviral response. J. Gen. Virol..

[386] Doki T., Yabe M., Takano T., Hohdatsu T. (2018). Differential induction of type I interferon by type I and type II feline coronaviruses in vitro. Res. Vet. Sci..

[387] Addie D.D., Ishida T., Bonagura J.D., Twedt D.C. (2008). Feline Infectious Peritonitis: Therapy and Prevention. Kirk’s Current Veterinary Therapy XIV.

[388] Mochizuki M., Nakatani H., Yoshida M. (1994). Inhibitory effects of recombinant feline interferon on the replication of feline enteropathogenic viruses in vitro. Vet. Microbiol..

[389] Weiss R.C., Toivio-Kinnucan M. (1988). Inhibition of feline infectious peritonitis virus replication by recombinant human leukocyte (alpha) interferon and feline fibroblastic (beta) interferon. Am. J. Vet. Res..

[390] Weiss R.C., Oostrom-Ram T. (1989). Inhibitory effects of ribavirin alone or combined with human alpha interferon on feline infectious peritonitis virus replication in vitro. Vet. Microbiol..

[391] Nishijima R., Endo T., Gankhuyag E., Khin S., Jafar S.M., Shinohara Y., Tanaka Y., Sawakami K., Yohda M., Furuya T. (2023). Detection of anti-feline infectious peritonitis virus activity of a Chinese herb extract using geneLEAD VIII, a fully automated nucleic acid extraction/quantitative PCR testing system. J. Vet. Med. Sci..

[392] van der Meer F.J., de Haan C.A., Schuurman N.M., Haijema B.J., Verheije M.H., Bosch B.J., Balzarini J., Egberink H.F. (2007). The carbohydrate-binding plant lectins and the non-peptidic antibiotic pradimicin A target the glycans of the coronavirus envelope glycoproteins. J. Antimicrob. Chemother..

[393] Mohseni N., Royster A., Ren S., Ma Y., Pintado M., Mir M., Mir S. (2023). A novel compound targets the feline infectious peritonitis virus nucleocapsid protein and inhibits viral replication in cell culture. J. Biol. Chem..

[394] Triratapiban C., Lueangaramkul V., Phecharat N., Pantanam A., Lekcharoensuk P., Theerawatanasirikul S. (2023). First study on in vitro antiviral and virucidal effects of flavonoids against feline infectious peritonitis virus at the early stage of infection. Vet. World.

[395] Camero M., Lanave G., Catella C., Lucente M.S., Sposato A., Mari V., Tempesta M., Martella V., Buonavoglia A. (2022). ERDRP-0519 inhibits feline coronavirus in vitro. BMC Vet. Res..

[396] Liu I.J., Tsai W.T., Hsieh L.E., Chueh L.L. (2013). Peptides corresponding to the predicted heptad repeat 2 domain of the feline coronavirus spike protein are potent inhibitors of viral infection. PLoS ONE.

[397] Choong O.K., Mehrbod P., Tejo B.A., Omar A.R. (2014). In vitro antiviral activity of circular triple helix forming oligonucleotide RNA towards Feline Infectious Peritonitis virus replication. BioMed Res. Int..

[398] Takano T., Endoh M., Fukatsu H., Sakurada H., Doki T., Hohdatsu T. (2017). The cholesterol transport inhibitor U18666A inhibits type I feline coronavirus infection. Antivir. Res..

[399] Doki T., Tarusawa T., Hohdatsu T., Takano T. (2020). In Vivo Antiviral Effects of U18666A Against Type I Feline Infectious Peritonitis Virus. Pathogens.

[400] McDonagh P., Sheehy P.A., Norris J.M. (2011). In vitro inhibition of feline coronavirus replication by small interfering RNAs. Vet. Microbiol..

[401] McDonagh P., Sheehy P.A., Norris J.M. (2015). Combination siRNA therapy against feline coronavirus can delay the emergence of antiviral resistance in vitro. Vet. Microbiol..

[402] Hartmann K., Ritz S. (2008). Treatment of cats with feline infectious peritonitis. Vet. Immunol. Immunopathol..

[403] Hartmann K., Ettinger S.J., Feldman E.C., Côté E. (2016). Coronavirus Infections (Canine and Feline), including Feline Infectious Peritonitis. Textbook of Veterinary Internal Medicine.

[404] Bolcskei A., Bilkei G. (1995). Langzeitstudie über behandelte FIP-verdächtige Katzen. Die Auswirkung verschiedener Therapieversuche auf das Überleben FIP-verdächtiger Katzen. Tierarztl. Umsch..

[405] Bolcskei A., Bilkei G. (1995). Langzeitstudie über behandelte FIP-verdächtige Katzen. Überlebensrate der FIP-verdächtigen Katzen nach Behandlung mit Ampicillin, Prednisolon und Cyclophosphamid. Tierarztl. Umsch..

[406] Colgrove D.J., Parker A.J. (1971). Feline infectious peritonitis. J. Small Anim. Pract..

[407] Ford R.B. (1986). Biological response modifiers in the management of viral infection. Vet. Clin. N. Am. Small Anim. Pract..

[408] Rodan I., Dowgray N., Carney H.C., Carozza E., Ellis S.L., Heath S., Niel L., St Denis K., Taylor S. (2022). 2022 AAFP/ISFM Cat Friendly Veterinary Interaction Guidelines: Approach and Handling Techniques. J. Feline Med. Surg..

[409] Taylor S., Chan D.L., Villaverde C., Ryan L., Peron F., Quimby J., O’Brien C., Chalhoub S. (2022). 2022 ISFM Consensus Guidelines on Management of the Inappetent Hospitalised Cat. J. Feline Med. Surg..

[410] Legendre A.M., Bartges J.W. (2009). Effect of Polyprenyl Immunostimulant on the survival times of three cats with the dry form of feline infectious peritonitis. J. Feline Med. Surg..

[411] Fischer Y., Ritz S., Weber K., Sauter-Louis C., Hartmann K. (2011). Randomized, Placebo Controlled Study of the Effect of Propentofylline on Survival Time and Quality of Life of Cats with Feline Infectious Peritonitis. J. Vet. Intern. Med..

[412] Doki T., Takano T., Kawagoe K., Kito A., Hohdatsu T. (2016). Therapeutic effect of anti-feline TNF-alpha monoclonal antibody for feline infectious peritonitis. Res. Vet. Sci..

[413] Watari T., Kaneshima T., Tsujimoto H., Ono K., Hasegawa A. (1998). Effect of thromboxane synthetase inhibitor on feline infectious peritonitis in cats. J. Vet. Med. Sci..

[414] Kuritz T. (2008). Methods and Compositions for Modulation of Innate Immunity. US Patent.

[415] Legendre A.M., Kuritz T., Heidel R.E., Baylor V.M. (2017). Polyprenyl Immunostimulant in Feline Rhinotracheitis: Randomized Placebo-Controlled Experimental and Field Safety Studies. Front. Vet. Sci..

[416] Tasker S., Dietrich U. Feline uveitis. Proceedings of the British Small Animal Association (BSAVA) Congress Proceedings 2022.

[417] Hartmann K., Ritz S., de Mari K. (2008). Feline Infectious Peritonitis. Clinical Case Veterinary Interferon Handbook.

[418] Gerber J.D. (1995). Overview of the Development of a Modified Live Temperature-Sensitive FIP Virus Vaccine. Feline Pract..

[419] Gerber J.D., Ingersoll J.D., Gast A.M., Christianson K.K., Selzer N.L., Landon R.M., Pfeiffer N.E., Sharpee R.L., Beckenhauer W.H. (1990). Protection against feline infectious peritonitis by intranasal inoculation of a temperature-sensitive FIPV vaccine. Vaccine.

[420] Hoskins J.D. (1995). The Potential Use of a Modified Live FIPV Vaccine to Prevent Experimental FECV Infection. Feline Pract..

[421] Hoskins J.D. (1995). Independent Evaluation of a Modified Live Feline Infectious Peritonitis Virus Vaccine Under Experimental Conditions (Louisiana Experience). Feline Pract..

[422] McArdle F., Tennant B., Bennett M., Kelly D.F., Gaskell C.J., Gaskell R.M. (1995). Independent Evaluation of a Modified Live FIPV Vaccine Under Experimental Conditions (University of Liverpool Experience). Feline Pract..

[423] Scott F.W., Corapi W.V., Olsen C.W. (1995). Independent Evaluation of a Modified Live FIPV Vaccine Under Experimental Conditions (Cornell Experience). Feline Pract..

[424] Scott F.W., Olsen C.W., Corapi W.V. (1995). Antibody-Dependent Enhancement of Feline Infectious Peritonitis Virus Infection. Feline Pract..

[425] Fehr D., Holznagel E., Bolla S., Hauser B., Herrewegh A.A.P.M., Horzinek M.C., Lutz H. (1995). Evaluation of the Safety and Efficacy of a Modified-Live FIPV Vaccine under Field Conditions. Feline Pract..

[426] Fehr D., Holznagel E., Bolla S., Hauser B., Herrewegh A.A., Horzinek M.C., Lutz H. (1997). Placebo-controlled evaluation of a modified life virus vaccine against feline infectious peritonitis: Safety and efficacy under field conditions. Vaccine.

[427] Postorino Reeves N. (1995). Vaccination against naturally occurring FIP in a single large cat shelter. Feline Pract..

[428] Addie D., Houe L., Maitland K., Passantino G., Decaro N. (2020). Effect of cat litters on feline coronavirus infection of cell culture and cats. J. Feline Med. Surg..

[429] Pedersen N.C. (2019). Fifty years’ fascination with FIP culminates in a promising new antiviral. J. Feline Med. Surg..

[430] Hickman M.A., Morris J.G., Rogers Q.R. (1995). Elimination of Feline Coronavirus Infection from a Large Experimental Specific Pathogen-Free Cat Breeding Colony by Serologic Testing and Isolation. Feline Pract..

[431] Pedersen N.C., Sato R., Foley J.E., Poland A.M. (2004). Common virus infections in cats, before and after being placed in shelters, with emphasis on feline enteric coronavirus. J. Feline Med. Surg..

[432] Addie D.D., Curran S., Bellini F., Crowe B., Sheehan E., Ukrainchuk L., Decaro N. (2020). Oral Mutian(R)X stopped faecal feline coronavirus shedding by naturally infected cats. Res. Vet. Sci..

[433] Strasfeld L., Chou S. (2010). Antiviral drug resistance: Mechanisms and clinical implications. Infect. Dis. Clin. N. Am..

[434] Cave T.A., Thompson H., Reid S.W., Hodgson D.R., Addie D.D. (2002). Kitten mortality in the United Kingdom: A retrospective analysis of 274 histopathological examinations (1986 to 2000). Vet. Rec..

[435] Bateson P., Immelmann K., Barlow G., Main M., Petrinovich L. (1981). Discontinuities in development and changes in the organisation of play in cats. Behavioural Development.

[436] Guyot G.W., Cross H.A., Bennett T.L. (1980). Early social isolation of the domestic cat: Responses to separation from social and nonsocial rearing stimuli. Dev. Psychobiol..

[437] Philip F., Seitz D. (1959). Infantile Experience and Adult Behavior in Animal Subjects. Psychosom. Med..

[438] Möstl K., Egberink H., Addie D., Frymus T., Boucraut-Baralon C., Truyen U., Hartmann K., Lutz H., Gruffydd-Jones T., Radford A.D. (2013). Prevention of infectious diseases in cat shelters: ABCD guidelines. J. Feline Med. Surg..

[439] Wagner D., Hurley K., Stavisky J. (2018). Shelter housing for cats: Practical aspects of design and construction, and adaptation of existing accommodation. J. Feline Med. Surg..

[440] Wagner D., Hurley K., Stavisky J. (2018). Shelter housing for cats: Principles of design for health, welfare and rehoming. J. Feline Med. Surg..

[441] Möstl K., Addie D.D., Boucraut-Baralon C., Egberink H., Frymus T., Gruffydd-Jones T., Hartmann K., Hosie M.J., Lloret A., Lutz H. (2015). Something old, something new: Update of the 2009 and 2013 ABCD guidelines on prevention and management of feline infectious diseases. J. Feline Med. Surg..

[442] Möstl K., Addie D.D., Belak S., Boucraut-Baralon C., Egberink H., Frymus T., Hartmann K., Hofmann-Lehmann R., Frymus T., Lloret A. (2021). Infectious Diseases in Shelter Situations and Their Management. http://www.abcdcatsvets.org/infectious-diseases-in-shelter-situations-and-their-management/.

[443] Cotter S.M., Gilmore C.E., Rollins C. (1973). Multiple cases of feline leukemia and feline infectious peritonitis in a household. J. Am. Vet. Med. Assoc..

[444] Tasker S., Addie D., Egberink H., Hartmann K., Hofmann-Lehmann R., Hosie M.J., Truyen U., Belak S., Boucraut-Baralon C., Frymus T. (2023). ABCD Guidelines Feline Infectious Peritonitis. http://www.abcdcatsvets.org/feline-infectious-peritonitis/.

